# Effect of sustained high buprenorphine plasma concentrations on fentanyl-induced respiratory depression: A placebo-controlled crossover study in healthy volunteers and opioid-tolerant patients

**DOI:** 10.1371/journal.pone.0256752

**Published:** 2022-01-27

**Authors:** Laurence M. Moss, Marijke Hyke Algera, Robert Dobbins, Frank Gray, Stephanie Strafford, Amy Heath, Monique van Velzen, Jules A. A. C. Heuberger, Marieke Niesters, Erik Olofsen, Celine M. Laffont, Albert Dahan, Geert Jan Groeneveld

**Affiliations:** 1 Centre for Human Drug Research (CHDR), Leiden, The Netherlands; 2 Department of Anesthesiology, Leiden University Medical Centre (LUMC), Leiden, The Netherlands; 3 Indivior Inc., North Chesterfield, Virginia, United States of America; Centre for Addiction and Mental Health, CANADA

## Abstract

**Background:**

Opioid-induced respiratory depression driven by ligand binding to mu-opioid receptors is a leading cause of opioid-related fatalities. Buprenorphine, a partial agonist, binds with high affinity to mu-opioid receptors but displays partial respiratory depression effects. The authors examined whether sustained buprenorphine plasma concentrations similar to those achieved with some extended-release injections used to treat opioid use disorder could reduce the frequency and magnitude of fentanyl-induced respiratory depression.

**Methods:**

In this two-period crossover, single-centre study, 14 healthy volunteers (single-blind, randomized) and eight opioid-tolerant patients taking daily opioid doses ≥90 mg oral morphine equivalents (open-label) received continuous intravenous buprenorphine or placebo for 360 minutes, targeting buprenorphine plasma concentrations of 0.2 or 0.5 ng/mL in healthy volunteers and 1.0, 2.0 or 5.0 ng/mL in opioid-tolerant patients. Upon reaching target concentrations, participants received up to four escalating intravenous doses of fentanyl. The primary endpoint was change in isohypercapnic minute ventilation (V_E_). Additionally, occurrence of apnea was recorded.

**Results:**

Fentanyl-induced changes in V_E_ were smaller at higher buprenorphine plasma concentrations. In healthy volunteers, at target buprenorphine concentration of 0.5 ng/mL, the first and second fentanyl boluses reduced V_E_ by [LSmean (95% CI)] 26% (13–40%) and 47% (37–59%) compared to 51% (38–64%) and 79% (69–89%) during placebo infusion (*p* = 0.001 and < .001, respectively). Discontinuations for apnea limited treatment comparisons beyond the second fentanyl injection. In opioid-tolerant patients, fentanyl reduced V_E_ up to 49% (21–76%) during buprenorphine infusion (all concentration groups combined) versus up to 100% (68–132%) during placebo infusion (*p* = 0.006). In opioid-tolerant patients, the risk of experiencing apnea requiring verbal stimulation following fentanyl boluses was lower with buprenorphine than with placebo (odds ratio: 0.07; 95% CI: 0.0 to 0.3; *p* = 0.001).

**Interpretation:**

Results from this proof-of-principle study provide the first clinical evidence that high sustained plasma concentrations of buprenorphine may protect against respiratory depression induced by potent opioids like fentanyl.

## Introduction

Opioid use disorder (OUD) is a major source of morbidity and mortality [1]. The opioid epidemic has been fuelled in recent years by increasingly widespread prescription and illicit opioid consumption for many indications [2–5], including the treatment of non-cancer pain [6]. Fatalities attributable to opioid misuse and overdose in the USA increased six-fold between 1999 and 2017 to an estimated 47,600 [7]. The alarming increase in mortality has been observed in other countries and is largely driven by the increasing use of fentanyl and fentanyl analogues, often surreptitiously mixed with heroin [8–10].

Potentially fatal respiratory depression is the main hazard associated with opioid use and abuse [11]. Opioid-induced respiratory depression (OIRD) is driven by ligand binding to mu-opioid receptors (MORs) expressed on neurons in brainstem respiratory centres [12]. Binding to MORs induces complex changes in respiratory regulation that result in increased arterial carbon dioxide concentrations and reduced tidal volume and minute ventilation [13]. Breathing slows and becomes irregular, potentially culminating in fatal apnea, the major cause of death in opioid overdose [14]. As an additional complication, development of tolerance to opioid analgesic/euphoric effects often precedes the development of tolerance to OIRD, which may lead to dangerous self-regulated dose escalation [15].

Buprenorphine has been proven as an effective medication for the treatment of OUD [16]. Buprenorphine is a semi-synthetic MOR partial agonist that binds to MORs with high affinity and slowly dissociates from the receptors, enabling it to displace MOR full agonists such as fentanyl and mitigate their physiological effects [17, 18]. Buprenorphine itself is associated with OIRD, but a study in healthy volunteers at intravenous bolus doses ranging from 0.05 to 0.60 mg/70 kg demonstrated an apparent maximum, or ceiling, effect on respiratory depression [19, 20]. Based on a pharmacokinetic-pharmacodynamic model of OIRD reversal, the authors previously proposed that at maximum buprenorphine MOR occupancy (S1 Fig), the effect of fentanyl on respiration would be limited, even at high fentanyl doses [12, 21]. The present study aimed to provide proof of principle for this hypothesis. The results of this study confirm that high sustained buprenorphine plasma concentrations can reduce the respiratory depression caused by injection of a potent, short-acting MOR full agonist such as fentanyl.

## Methods

### Trial design

This was a two-part, placebo-controlled crossover study. Both Parts A and B included two study periods, during which participants received continuous intravenous infusion of buprenorphine or placebo co-administered with up to four escalating fentanyl doses. For healthy volunteers in Part A, treatment sequence was randomly assigned so participants received placebo or buprenorphine infusion during Period 1 and the alternate infusion during Period 2. Because tolerance to opioid effects is poorly characterized in patients receiving long-term opioids, opioid-tolerant patients in Part B had a fixed treatment sequence, receiving placebo infusion plus fentanyl challenges in Period 1 to optimize the fentanyl dose escalation before buprenorphine and fentanyl were co-administered in Period 2.

There were no major changes to trial design after commencement of each study part, other than an amendment of the eligibility criteria for Part B to enable recruitment of a broader group of patients. Changes regarded concurrent use of CNS depressants (e.g. benzodiazepines), inclusion of smokers (measurements not affected), and exclusion of patients with clinically significant risks of Torsades de Pointes instead of a history of risk factors. There were no changes to trial endpoints after the trial commenced.

### Participants

The study enrolled healthy volunteers (Part A) and opioid-tolerant patients (Part B). All participants provided written informed consent prior to any study-related procedure and screening was completed within 30 days of the first study drug administration. In Part A, male and female healthy volunteers, aged 18 to 45 years with a body mass index of 18 to 30 kg/m^2^, were eligible. Exclusion criteria included history of any clinically relevant medical, psychiatric, or neurologic condition; positive pregnancy test; history of current substance use disorder according to the criteria of the Diagnostic and Statistical Manual of Mental Disorders, 5th edition; [22] smoking or having smoked in the last 6 months; alcohol consumption >20 units/week (men) or >13 units/week (women); use of any medication within 14 days or 5 half-lives before dosing; opioid use (including opioid antagonists) within 30 days before dosing; use of medication that induces/inhibits relevant cytochrome P450 enzymes; history of suicidal ideation within 30 days or suicide attempt within 6 months prior to informed consent; or any other condition that, in the opinion of the investigators, could interfere with the ability to participate in the study.

For Part B, male and female opioid-tolerant patients, aged 18 to 55 years, with a body mass index of 18 to 32 kg/m^2^ using daily doses of opioids ≥ 90 mg oral morphine equivalents [23], and who were in stable condition based on their medical evaluation were eligible. All exclusion criteria were similar to Part A, except for modified alcohol consumption limits to >27 units/week (men) or >20 units/week (women); broadened nicotine permissions to no smoking on dosing days; and specifically no use of buprenorphine within 10 days of the first study drug administration. Opioid-tolerant patients were recruited through national advertisements, out-patient clinics with expertise in the treatment of pain, and in collaboration with specialized opioid-abuse treatment clinics.

All eligibility criteria are provided in the study protocol, which is available as S1 File.

#### Setting and location of data collection

This study was conducted in Leiden, The Netherlands. Dosing day procedures were performed at the department of anaesthesiology of the Leiden University Medical Centre (LUMC) and all other activities regarding trial execution were performed at the Centre for Human Drug Research (CHDR). The study was conducted in accordance with the principles of the Declaration of Helsinki, the International Conference on Harmonisation Good Clinical Practice (ICH GCP), and ethical principles as referenced in EU Directive 2001/20/EC. The protocol (EudraCT 2017‐004858‐42) was approved by the Medical Review and Ethics Committee of the BEBO foundation (Assen, The Netherlands).

### Interventions

Healthy volunteers in Part A were admitted the day prior to the experiment for each study period, with a washout of two weeks between periods. Opioid-tolerant patients in Part B were admitted to the clinic 2–5 days before the first study period and remained in the clinic until completion of the both study periods. To ensure washout of each patient’s usual opioids, tailored substitution schedules with oxycodone began a minimum of 48 hours before Period 1, and the last dose of oxycodone was administered at least 15 hours before study drug administration. Due to the short half-life of fentanyl, Period 2 was separated from Period 1 by 40 hours. During this washout period, patients again received oxycodone for opioid substitution.

On the morning of each study period, an intravenous line was placed for administration of study medication and an arterial line was placed for blood sampling in the opposite arm. Isohypercapnic ventilation was measured during buprenorphine/placebo infusion for approximately 6 hours using the dynamic end-tidal forcing technique, as described elsewhere [20, 21], allowing the investigator to direct ventilation towards pre-defined end-tidal PCO_2_ (7 kPa) and PO_2_ (14.5 kPa) values. A combination of oxygen, carbon dioxide, and nitrogen was delivered to the participants through a face mask and inspired minute ventilation was measured by pneumotachography. A finger probe with pulse oximeter was used for continuous surveillance of arterial oxygen saturation (SpO_2_). These ventilation parameters were captured as one-minute breath-to-breath averages.

Intravenous infusion with buprenorphine (Indivior UK Ltd., UK) or placebo started once baseline minute ventilation (V_E_) had stabilized at 20 ± 2 L/min (about 4-fold above normal resting V_E_). In healthy volunteers, an infusion rate of 0.02 or 0.05 mg/70 kg/h buprenorphine was selected to target plasma concentrations of 0.2 or 0.5 ng/mL, respectively. In opioid-tolerant patients, higher buprenorphine infusion rates were administered: 0.1, 0.2 or 0.5 mg/70 kg/h targeting plasma concentrations of 1.0, 2.0 or 5.0 ng/mL, respectively. In both healthy volunteers and opioid-tolerant patients, a 10-fold higher infusion rate was used over the first 15 minutes to speed attainment of steady-state buprenorphine concentrations at the site of action. In order to manage possible gastrointestinal side effects, all participants received 4 mg of ondansetron prior to infusion.

At 120, 180, 240, and 300 minutes after the start of the buprenorphine or placebo infusion, escalating intravenous fentanyl doses (Hameln Pharmaceuticals Ltd., UK) were administered over 90 seconds. The planned fentanyl doses in healthy volunteers were 0.075, 0.15, 0.25 and 0.35 mg/70 kg. In opioid-tolerant patients, the planned fentanyl doses were 0.25, 0.35, 0.50 and 0.70 mg/70 kg.

Arterial blood samples for analysis of buprenorphine and fentanyl plasma concentrations were collected at multiple timepoints over 540 minutes after the start of buprenorphine or placebo infusion. Buprenorphine and fentanyl plasma concentrations were assessed using liquid chromatography with tandem mass spectrometry (LC-MS/MS) methods validated over a range of 0.02 to 10.0 ng/mL for buprenorphine and 0.1 to 50.0 ng/mL for fentanyl.

### Pharmacodynamic and pharmacokinetic outcomes

The primary study endpoint was maximum decrease in minute ventilation, defined as the minimum value of isohypercapnic V_E_ observed during each fentanyl dosing period compared to pre-fentanyl baseline. The pre-fentanyl baseline value was defined as the average of the last 5 minutes prior to the first fentanyl dose. Secondary endpoints included the number and percentage of participants who experienced apnea (defined as ≥20 s loss of respiratory activity) and required verbal stimulation to breath after a fentanyl dose. Any subject who desaturated below 92% without spontaneous recovery within seconds after, was verbally stimulated to breathe, regardless of intervention.

Buprenorphine average plasma concentration (C_avg_) at steady-state was calculated as the area under the plasma concentration-time curve between 120 and 360 minutes after the start of buprenorphine infusion divided by the time interval. Treatment-emergent adverse events (TEAEs) were recorded from time of first screening visit through the end of the last visit. Fentanyl dose escalation was halted if a participant did not breathe for a prolonged period or SpO_2_ dropped below 85% despite active verbal stimulation by the investigator, or if the investigators deemed necessary (i.e. other TEAEs). Drug plasma concentrations and safety measures (SpO2, TEAEs) were exploratory endpoints.

### Sample size

In the absence of informed priors for the interaction between fentanyl and buprenorphine, no a priori sample size calculation was performed, and statistical testing was descriptive. A post-hoc power analysis, calculated by paired sample t-test for the primary endpoint, showed that a sample size of 8 yields >96% power, when the treatment difference is 50.8%, standard deviation is 32.7% and alpha is set to 0.05 two-sided.

### Randomisation

#### Sequence generation

Healthy volunteers in Part A were randomly assigned to one of two treatment sequences (buprenorphine-placebo or placebo-buprenorphine). A blocked randomisation schedule was generated by an independent statistician using SAS version 9.4. A block size of 2 was chosen to ensure the best possible balancing if the study would be prematurely halted.

Part B was an open-label, single-sequence crossover study where participants received placebo treatment and then buprenorphine treatment.

#### Allocation concealment and implementation

For Part A, participants were single-blinded. The independent statistician who generated the random allocation sequence was not involved in recruiting nor randomising participants. To prevent selection bias, CHDR staff not involved in generating the random allocation sequence assigned the randomisation numbers to participants sequentially, in the order of completed medical screenings. An independent LUMC study pharmacist prepared masked infusion syringes for administration by LUMC staff.

Treatment sequence for Part B was not randomised. Dose group allocation in Part B was performed by the investigators within dose ranges specified per protocol.

### Statistical methods

To reduce the impact of technical artifacts that could introduce measurement noise on V_E_ measures, analysis of V_E_ endpoints was conducted after post-hoc adjustment of data sets. The adjusted data sets reflect imputations based on clinical notes to account for the impact of concurrent clinical events such as facemask removal, urinating with facemask on and severe itching. Stimulated, nonspontaneous breathing data were set at zero (apnea) for analyses on ventilation data.

Maximum percent decreases in V_E_ relative to baseline were compared between treatment groups using a mixed effects model with treatment as a fixed effect. For Part B, all buprenorphine concentration groups were combined to perform the treatment comparison. Maximum percent decreases in V_E_ were assessed within the first 10 minutes after each fentanyl bolus to accurately characterise the peak pharmacodynamic effect of fentanyl by minimizing the impact of random variation evident over the full 60-minute intervals. Secondary endpoints were compared between treatment groups by exact conditional logistic regression and Fisher’s exact test. The exploratory safety endpoint (SpO_2_) was analysed in a similar manner to changes in V_E_.

The primary and secondary endpoint analyses were performed on participants who received at least 1 dose of fentanyl and had at least 1 post-dose assessment, excluding one participant who received the wrong buprenorphine infusion rate. TEAEs were summarized for participants who received at least one dose of study medication. The buprenorphine and fentanyl plasma concentrations were summarized for all participants who received at least 1 dose of the medication and had an adequate number of pharmacokinetic samples collected.

Statistical analyses were performed using SAS version 9.4.

## Results

**Fig 1:**
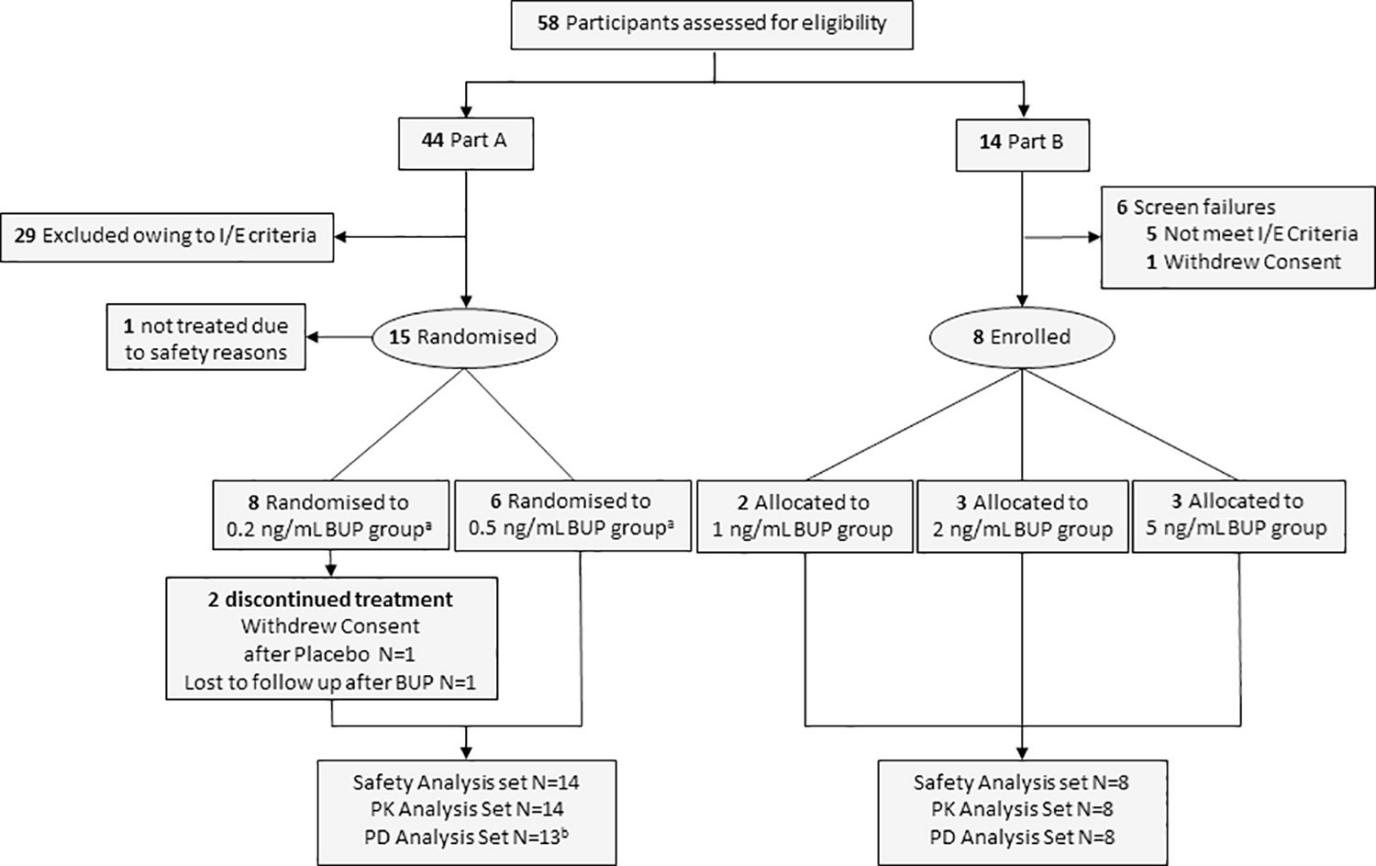
CONSORT flow diagram. BUP, buprenorphine; PD, pharmacodynamic; PK, pharmacokinetic. ^a^Randomised sequences for Part A were Placebo:BUP N = 5, BUP:Placebo N = 3 for the 0.2 ng/mL group and Placebo:BUP N = 2, BUP:Placebo N = 4 for the 0.5 ng/mL group. ^b^One volunteer in the lower dose group received the incorrect buprenorphine dose and was excluded from the PD analyses. Data were available for six healthy volunteers in each treatment (placebo and buprenorphine) for the PD analyses in the lower dose group due to two volunteers in the lower dose group who completed only one study period.

In total, 58 participants were screened for the study, which commenced on 22 March 2018 and completed on 04 January 2019, enrolling a total of 22 participants. Fourteen healthy volunteers and eight opioid-tolerant patients who used high-dose opioids for at least three months (range 0.25–29 years; see Table 1 for baseline characteristics) were included in the study. The CONSORT diagram summarizes participant disposition (Fig 1).

**Table 1:** Participant demographic and clinical characteristics.

	Part A: Healthy Volunteers		Part B: Opioid-tolerant Patients			
Buprenorphine concentration	0.2 ng/mL	0.5 ng/mL	1 ng/mL	2 ng/mL	5 ng/mL	Grouped
	(n = 8)	(n = 6)	(n = 2)	(n = 3)	(n = 3)	(n = 8)
Sex, N (%)						
Male	4 (50)	3 (50)	1 (50)	1 (33)	1 (33)	3 (38)
Female	4 (50)	3 (50)	1 (50)	2 (67)	2 (67)	5 (63)
Age, mean (SD) or range, y	23.8 ± 4.6	24.5 ± 2.4	44–46	31–43	34–52	42 ± 8
Ethnicity, N (%)						
White	8 (100)	5 (83)	2 (100)	3 (100)	3 (100)	8 (100)
Native Hawaiian		1 (17)				
Weight, mean (SD) or range, kg	74.2 ± 6.9	67.9 ± 6.6	70–93	70–87	65–89	78 ± 10
BMI, mean (SD) or range, kg/m^2^	23.5 ± 2.2	22.4 ± 1.6	23.6–29.6	22.0–30.8	21.0–31.5	25.9 ± 4.2
Daily MME, mean (SD) or range, mg	NA	NA	90–150	90–480	90–270	203 ± 135
Drug Usage per Participant^a^	NA	NA	• Oxycodone 60 mg/d	• Fentanyl patch 75 mcg/h; oxycodone 90 mg/d; tapentadol 50 mg/d	• Heroin 250 mg/d (smoke); cocaine; marijuana	
			• Fentanyl patch 25 mcg/h; oxycodone 60 mg/d; marijuana	• Buprenorphine 16 mg/d; cocaine; marijuana	• Fentanyl patch 50 mcg/h	
				• Oxycodone 60 mg/d; marijuana	• Fentanyl patch 75 mcg/h; oxycodone 60 mg/d; marijuana	

BMI, body mass index; MME, Morphine Milligram Equivalents; N, sample size; NA, not applicable; SD: standard deviation.

^a^Tailored substitution schedules with oxycodone began a minimum of 48 hours before the first experiment to ensure washout of each patient’s usual opioids at baseline.

**Fig 2:**
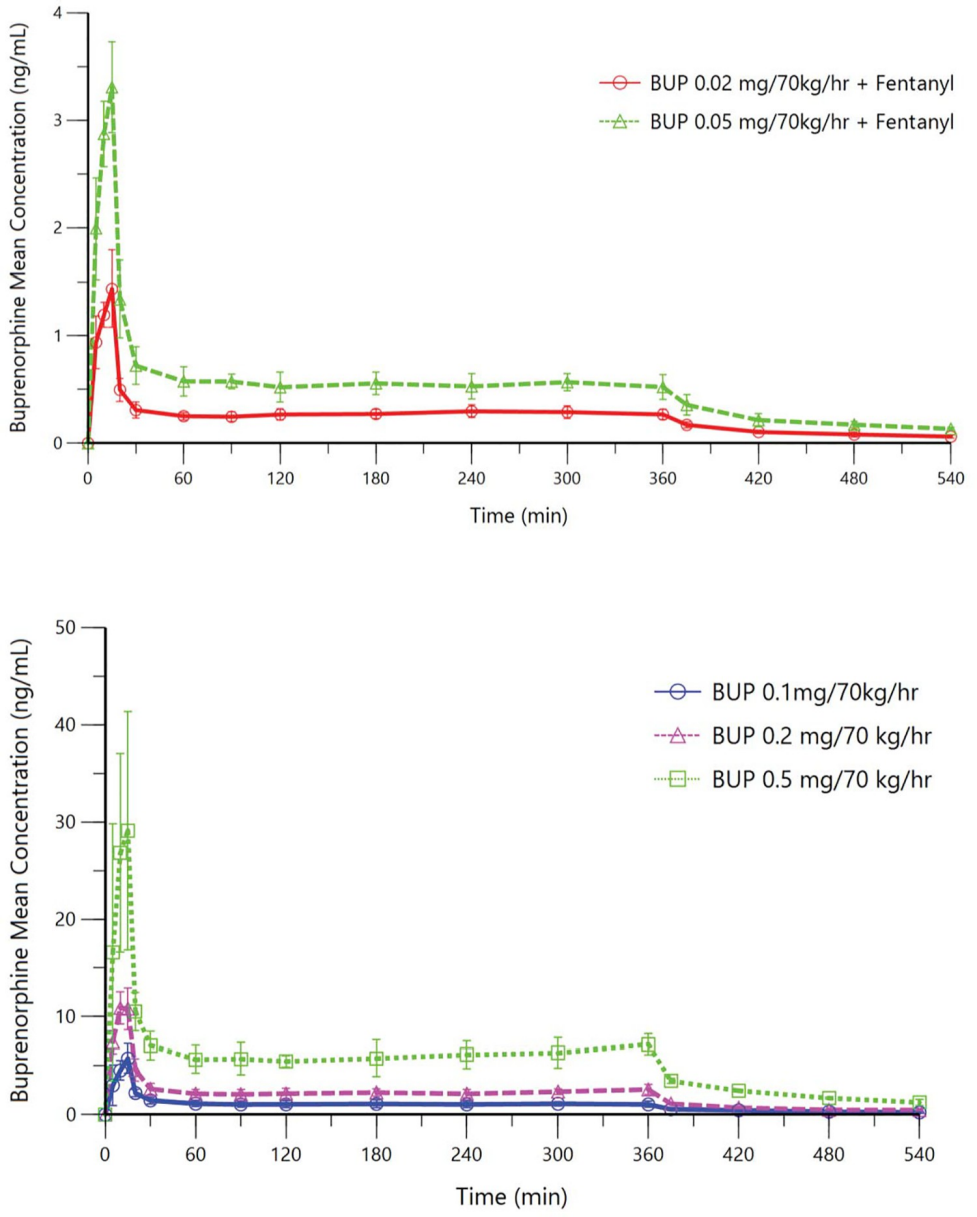
Mean buprenorphine plasma concentration-time curves. Upper panel: Part A, healthy volunteers; Lower panel: Part B, opioid-tolerant patients. In both healthy volunteers and opioid-tolerant patients, a 10-fold higher infusion rate was used over the first 15 minutes to speed attainment of steady-state buprenorphine concentrations at the site of action. Infusions were stopped at 360 min. Steady-state buprenorphine infusion rates are labelled in the graphs.

**Fig 3:**
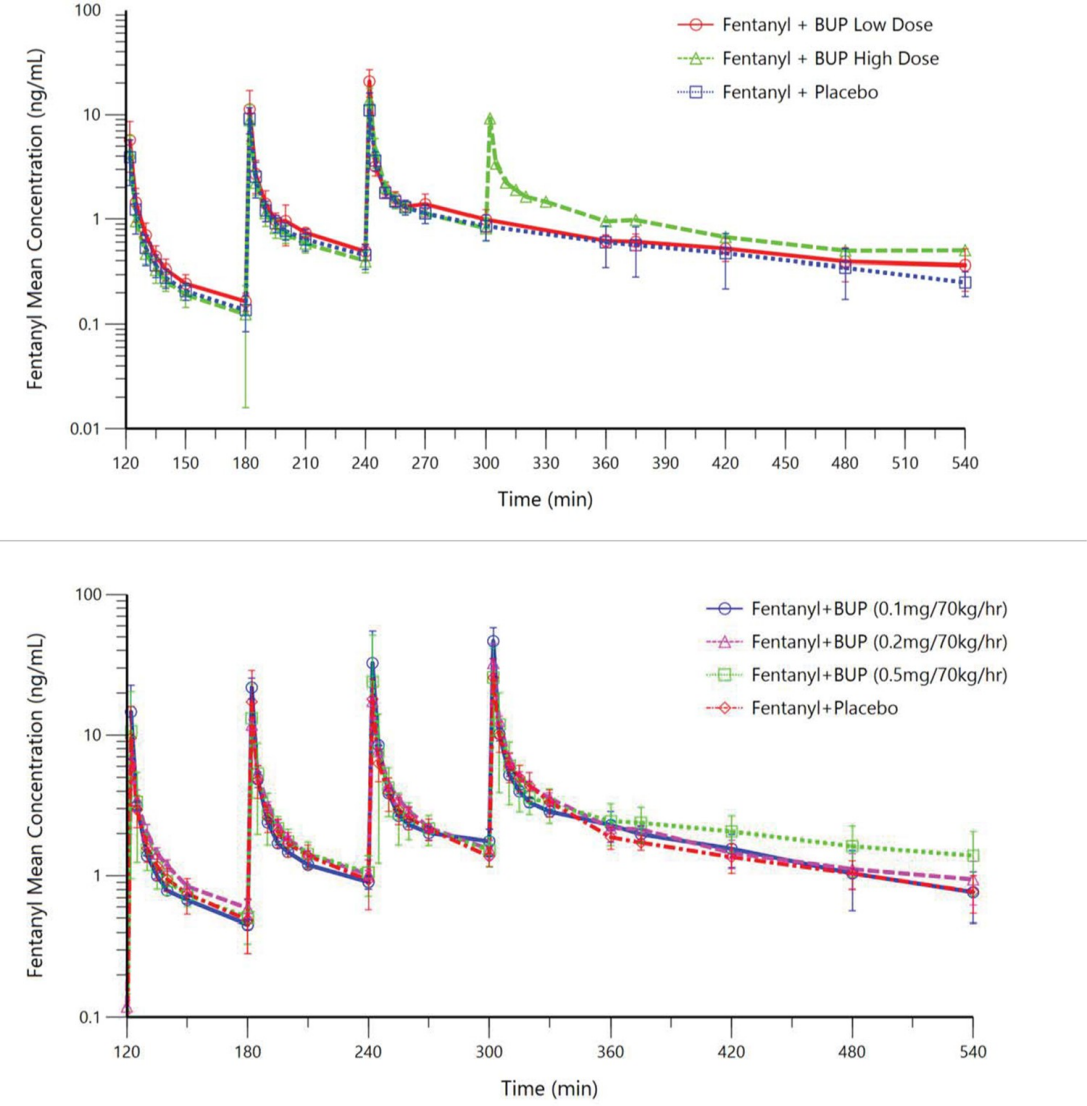
Mean fentanyl plasma concentration-time curves. Upper panel: Part A, healthy volunteers; Lower panel: Part B, opioid-tolerant patients. At 120, 180, 240, and 300 minutes after the start of the buprenorphine or placebo infusion, escalating intravenous fentanyl doses were administered over 90 seconds. Planned fentanyl bolus doses are labelled in the graphs. Higher doses were not administered to participants if they did not tolerate lower fentanyl doses.

In healthy volunteers, steady-state buprenorphine plasma concentrations (mean ± SD) were 0.28 ± 0.05 and 0.54 ± 0.08 ng/mL, respectively (Fig 2), consistent with the target concentrations of 0.2 and 0.5 ng/mL. In opioid-tolerant patients, steady-state buprenorphine plasma concentrations were 1.08 ± 0.33, 2.28 ± 0.40, and 6.12 ± 1.26 ng/mL, respectively (Fig 2), all consistent with the targeted concentrations. Mean fentanyl plasma concentrations are shown for both participant populations in Fig 3. Table 2 lists fentanyl doses administered to healthy volunteers and opioid-tolerant patients and results for the number of participants who experienced persistent apnea that required verbal stimulation.

**Table 2:** Number and percentage of participants who experienced apnea that required stimulation (i.e. persistent apnea).

	Part A: Healthy Volunteers					Part B: Opioid-tolerant Patients		
Fentanyl Dose	Fentanyl Dose Number	Placebo for 0.2 ng/mL (N = 6)	Buprenorphine 0.2 ng/mL (N = 6)	Placebo for 0.5 ng/mL (N = 6)	Buprenorphine 0.5 ng/mL (N = 6)	Fentanyl Dose Number	Placebo (N = 8)	Buprenorphine^a^ (N = 8)
0.075 mg/70 kg	1	0/6 (0)	0/6 (0)	0/6 (0)	0/6 (0)			
0.15 mg/70 kg	2	1/6 (17)	0/4 (0) ^b^	0/6 (0)	0/6 (0)			
0.25 mg/70 kg	3	2/2 (100)^b^	2/4 (50)	3/4 (75)^b^	1/6 (17)	1	0/8 (0)	0/8 (0)
0.35 mg/70 kg	4	0/0	0/0^b^	0/0^b^	0/1 (0)^b^	2	2/8 (25)	0/8 (0)
0.50 mg/70 kg						3	1/6 (17)	0/8 (0)
0.70 mg/70 kg						4	3/4 (75)^b^	0/8 (0)

^a^The three buprenorphine dose groups in opioid-tolerant patients (target plasma concentrations of 1, 2 and 5 ng/mL) were grouped for this analysis.

^b^Some participants did not receive some fentanyl doses due to adverse events, apnea events that did not require stimulation or abnormalities in other ventilatory parameters (i.e. unstable breathing, drop in ventilation or saturation and high end-tidal CO_2_).

During the placebo study periods, five of the six healthy volunteers (83%) who progressed to the third fentanyl dose had persistent apnea versus only three out of ten (30%) during the buprenorphine study period. Four opioid-tolerant patients progressed to the fourth fentanyl bolus during the placebo period, three of which (75%) experienced persistent apnea. In contrast, all eight opioid-tolerant patients progressed to the fourth bolus during the buprenorphine study period, and none of them (0%) experienced persistent apnea. In opioid-tolerant patients, the risk of experiencing apnea requiring verbal stimulation following fentanyl boluses was significantly lower when receiving buprenorphine than when receiving placebo, with an odds ratio of 0.07 (95% CI, 0.0 to 0.3; *p* = 0.001).

**Fig 4:**
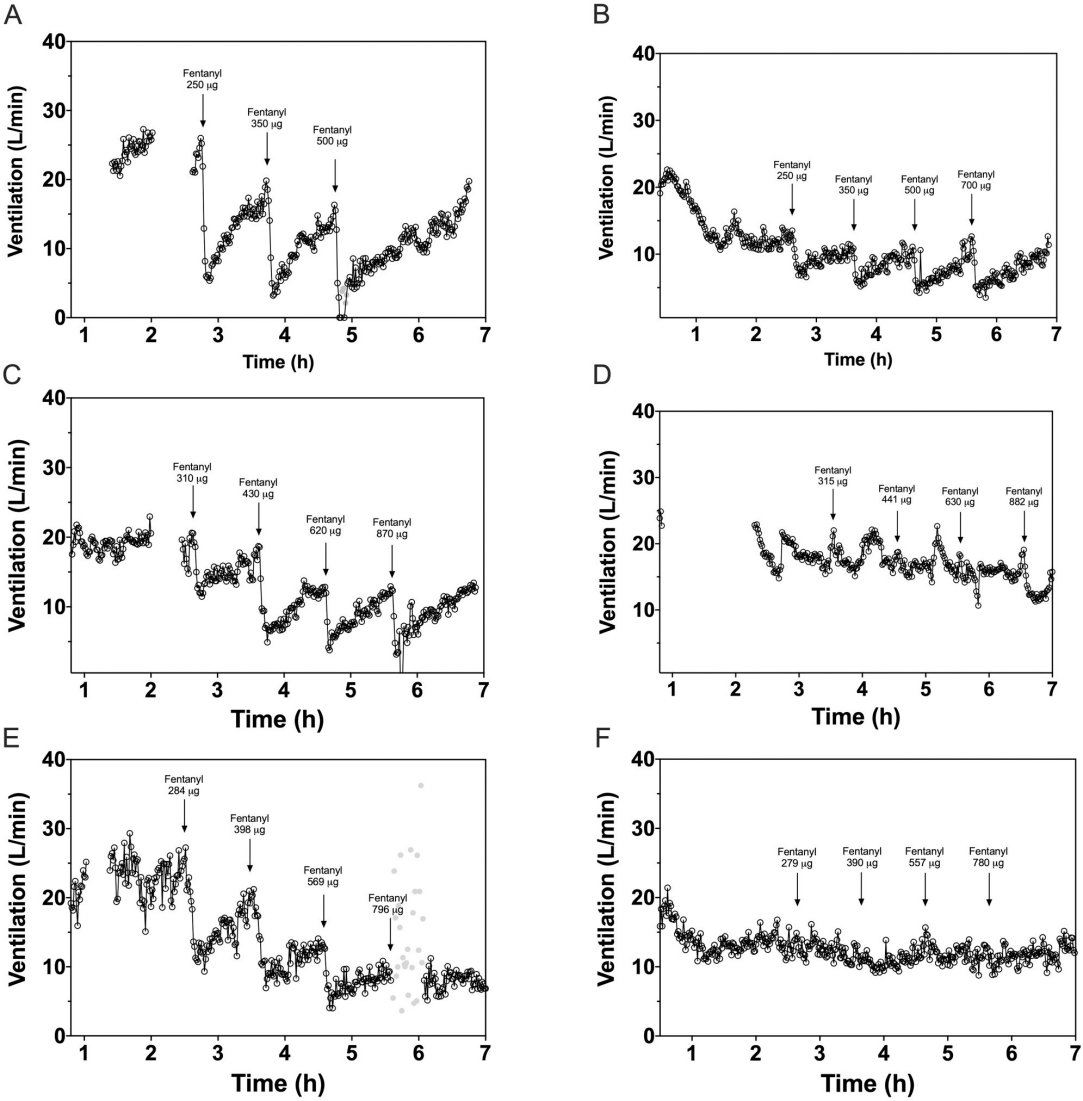
Example graphs showing the effect of fentanyl on minute ventilation in three opioid-tolerant patients during placebo infusion and buprenorphine infusion. (1) Placebo infusion (A, C and E) and buprenorphine infusion (B, D, F) at target plasma concentrations of 1 ng/mL (top row), 2 ng/mL (middle row) and 5 ng/mL (lower row). (2) Open spaces in the beginning of graphs A, C, D and E relate to concurrent clinical events such as temporary removal of the facemask. (3) Grey dots are stimulated breaths in case of an apnea episode. (4) The time on the x-axis in the graphs is related to the start time of the ventilation experiment, not the timing of the buprenorphine/placebo infusion and fentanyl injections.

In opioid-tolerant patients, fentanyl reduced V_E_ up to 49% (21–76%) during buprenorphine infusion (all concentration groups combined) versus up to 100% (68–132%) during placebo infusion (*p* = 0.006). Example tracings for representative opioid-tolerant patients in the 1, 2 and 5 ng/mL concentration groups, show V_E_ during the placebo and buprenorphine infusion study periods (Fig 4) and graphs depicting individual V_E_ per concentration level are provided as S2 Fig. The tracings indicate that buprenorphine itself decreased V_E_ compared to placebo; in healthy volunteers, the decrease in ventilation caused by buprenorphine was more pronounced. After fentanyl injections, significant treatment differences for healthy volunteers in the 0.5 ng/mL buprenorphine versus placebo groups were observed, with lower decreases in V_E_ [least squares mean difference (95% CI), *p*-value] following the first [25.1% (13.4–36.8%), 0.001] and second [31.6%, (19.3–43.8%), < .001] fentanyl bolus compared to pre-fentanyl baseline (Table 3). For the combined group of opioid-tolerant patients, significantly smaller reductions in V_E_ after fentanyl bolus 1 [29.9% (19.6–40.3%), < .001], 2 [42.8%, (23.8–61.8%), 0.001], 3 [39.4%, (15.7–63.1%), 0.008], and 4 [50.8%, (27.7–73.9%), 0.006] were measured when patients received buprenorphine infusion compared to placebo (Table 3). When the three buprenorphine concentration groups were compared in opioid-tolerant patients, fentanyl effects on V_E_ appeared greater for the 1 ng/mL group than for the 2 and 5 ng/mL groups.

**Table 3:** Maximum decreases in minute ventilation after fentanyl bolus administration (%).

		Healthy volunteers						Opioid-tolerant patients						
		0.2 ng/mL group			0.5 ng/mL group			1 ng/mL group		2 ng/mL group		5 ng/mL group		
		Placebo	BUP	Treatment Difference	Placebo	BUP	Treatment Difference	Placebo	BUP	Placebo	BUP	Placebo	BUP	Treatment Difference^b^
**Fentanyl Dose 1** ^a^	N	6	6		6	6		2	2	3	3	3	3	
	LSM (95% CI)	-60.9 (-73.5, -48.2)	-53.1 (-65.8, -40.5)	7.7 (-3.8, 19.3)	-51.3 (-64.3, -38.3)	-26.2 (-40.0, -12.5)	25.1 (13.4, 36.8)	-82.3 (-105.2, -59.3)	-49.2 (-72.1, -26.3)	-44.3 (-63.0, -25.6)	-22.2 (-40.9, -3.5)	-62.5 (-81.2, -43.8)	-26.8 (-45.5, -8.1)	29.9 (19.6, 40.3)
	*p*-value			0.1590			**0.001**							**< .001**
**Fentanyl Dose 2** ^a^	N	6	4		6	6		2	2	3	3	3	3	
	LSM (95% CI)	-82.4 (-92.7, -72.0)	-70.3 (-82.8, -57.8)	12.1 (-2.5, 26.7)	-79.0 (-89.4, -68.6)	-47.4 (-57.8, -37.0)	31.6 (19.3, 43.8)	-93.5 (-122.3, -64.6)	-57.3 (-86.1, -28.4)	-68.4 (-91.9, -44.8)	-35.9 (-59.5, -12.4)	-87.5 (-111.0, -63.9)	-30.0 (-53.5, -6.5)	42.8 (23.8, 61.8)
	*p*-value			0.0916			**< .001**							**0.001**
**Fentanyl Dose 3** ^a^	N	2	4		4	6		1	2	3	3	2	3	
	LSM (95% CI)	-100.0 (-142.3, -57.7)	-83.2 (-113.2, -53.3)	16.8 (-35.1, 68.7)	-93.6 (-123.6, -63.7)	-71.9 (-96.4, -47.5)	21.7 (-16.9, 60.4)	-100.0 (-145.3, -54.7)	-71.8 (-103.8, -39.8)	-79.3 (-105.5, -53.2)	-46.1 (72.3, -20.0)	-88.1 (-120.1, -56.1)	-30.7 (-56.9, -4.6)	39.4 (15.7, 63.1)
	*p*-value			0.3788			0.1716							**0.008**
**Fentanyl Dose 4** ^a^	N	0	0		0	1		0	2	3	3	1	3	
	LSM (95% CI)	NA	NA	NA	NA	NA	NA	NA	-68.7 (-140.2, 2.8)	-82.3 (-140.7, -23.9)	-33.7 (-92.1, 24.7)	-116.3 (-189.3, -42.2)	-50.5 (-108.9, 7.8)	50.8 (27.7, 73.9)
	*p*-value			NA			NA							**0.006**

BUP, buprenorphine; CI, confidence interval; LSM, least square mean; NA, not applicable. *p*<0.05 are presented in bold. Differences are LSM estimated treatment differences between buprenorphine and placebo.

^a^Maximum changes (%) in minute ventilation during first 10 minutes after each fentanyl administration compared to pre-fentanyl baseline.

^b^ The three buprenorphine concentration level groups in patients were grouped for this analysis.

All participants in each treatment period reported at least one TEAE and at least one TEAE-related to buprenorphine/placebo treatment. Overall, most events were mild or moderate in severity. In healthy volunteers, the most frequent TEAEs were nausea, apnea, and somnolence in both periods. The most frequent TEAEs in opioid-tolerant patients were apnea, dizziness, and somnolence. In the placebo period, 88% of opioid-tolerant patients experienced apnea compared to 13% during the buprenorphine period. Apneas reported as TEAEs did not necessarily require verbal stimulation. All the TEAEs were expected for administration of opioid agonists, including a high incidence of nausea among healthy volunteers who were opioid-naive.

In opioid-tolerant patients, SpO_2_ levels were significantly decreased after placebo treatment relative to buprenorphine after the first, third and fourth fentanyl boluses (Table 4). No other consistent differences in safety parameters were observed between treatment groups.

**Table 4:** Maximum change from pre-fentanyl baseline in oxygen saturation (%) in opioid-tolerant patients.

	Placebo	Buprenorphine	Treatment Difference (BUP-Placebo)
**Fentanyl Dose 1**			
N	8	8	
LS mean	-2.9	-1.5	1.4
95% CI	-3.8, -1.9	-2.4, -0.6	0.1, 2.7
*P*-value			**0.041**
**Fentanyl Dose 2**			
N	8	8	
LS mean	-5.2	-3.5	1.8
95% CI	-8.2, -2.3	-6.4, -0.5	-2.4, 5.9
*P*-value			0.353
**Fentanyl Dose 3**			
N	6	8	
LS mean	-8.1	-2.7	5.4
95% CI	-12.1, -4.2	-6.2, -0.7	0.8, 10.0
*P*-value			**0.030**
**Fentanyl Dose 4**			
N	4	8	
LS mean	-11.2	-2.6	8.6
95% CI	-14.7, -7.6	-5.0, -0.1	4.3, 12.9
*P*-value			**0.008**

CI, confidence interval; LS, least squares. (p<0.05) are presented in bold.

## Discussion

The present study is, to the best of our knowledge, the first to provide clinical evidence for the protective effects of buprenorphine in limiting fentanyl-induced respiratory depression. Previous studies in animal models and in healthy volunteers have shown that respiratory depression induced by buprenorphine is characterized by a ceiling effect at higher concentrations [19–21]. It was demonstrated that, unlike some other opioids, respiratory depression associated with buprenorphine is relatively resistant to naloxone reversal, likely because of high receptor affinity and slow dissociation from the receptor [17, 21]. The authors hypothesized that because of its special properties, high concentrations of buprenorphine that sustain maximum MOR occupancy could limit the extent of OIRD induced by fentanyl, a potent MOR full agonist.

The results demonstrate that in patients with higher tolerance to the effects of opioids, sustained high plasma concentrations of buprenorphine significantly reduced the magnitude of fentanyl-induced respiratory depression relative to placebo. This effect was observed with escalating fentanyl doses up to 0.70 mg/70 kg (total administered dose 1.8 mg/70kg over 180 minutes). Each fentanyl bolus was infused over 90 seconds, resulting in an immediate ventilatory response. This pharmacodynamic effect was well defined within the first 10 minutes of each fentanyl bolus; the ventilatory response slowly decreased thereafter (i.e. breathing recovered) and became more susceptible to random variation the longer after a bolus was administered. Apneic periods directly following a drug injection can be fatal in real-life situations. Therefore, it was regarded justified to only include the ventilatory response during the first 10 minutes after each bolus in the analysis. Buprenorphine administration was itself associated with a decrease in V_E_, but at the highest dose there was little or no additional decrease after subsequent fentanyl administration. The numbers in each buprenorphine dose group of opioid-tolerant patients were small, but there was a trend consistent with a buprenorphine concentration-response with highest levels of buprenorphine achieving greater suppression of V_E_ as evidenced by the tracings in Fig 4 (suggesting greater MOR occupancy). The impact of the fourth fentanyl bolus on V_E_ appears to be greater in the highest buprenorphine dose group than in the middle dose group of opioid-tolerant patients. This is due to a few isolated low values directly following fentanyl administration in two thirds of patients in the high-dose group. These data points were not excluded from the analysis but might be considered outliers. Because dose level groups were small, the statistical analysis was performed by grouping data across the buprenorphine dose levels.

Apnea events were less frequent and less severe following fentanyl administration during buprenorphine infusion than during placebo infusion. Opioid-tolerant patients treated with the highest dose of buprenorphine had no meaningful apnea events or changes in SpO_2_ after fentanyl boluses. At lower buprenorphine doses, fentanyl had an appreciable effect on ventilation. These results are consistent with the expected greater MOR occupancy at higher buprenorphine plasma levels [24]. Inhibition of fentanyl-induced respiratory depression by buprenorphine in healthy volunteers was observed only to a limited extent in this study. Although fentanyl did cause respiratory depression during buprenorphine infusion in healthy volunteers, especially at high-dose buprenorphine infusion, the decrease in V_E_ was significantly lower compared to the fentanyl effect during placebo infusion. Comparisons were difficult for the third and fourth fentanyl boluses, as only six of 12 healthy volunteers progressed to the third bolus during the placebo period compared to ten during the buprenorphine treatment. The only healthy volunteer who tolerated all four fentanyl boluses received buprenorphine at the highest dose. Collectively, the results suggest that buprenorphine at high concentrations reduces respiratory depression induced by fentanyl administration and suggest that sustained high concentrations of buprenorphine, such as those achieved with some extended-release injections used to treat OUD [25], may protect against inadvertent fentanyl overdose.

A possible limitation of this study is the relatively small number of participants with limited racial diversity. Moreover, the opioid-tolerant patient group is somewhat heterogeneous, including six patients chronically using opioids for pain, and two chronic drug abusers, and might not fully represent the real-world population of patients with OUD. However, ventilatory responses to buprenorphine and fentanyl were consistent between all opioid-tolerant patients with relatively low inter-subject variability. In addition, the observed effects of buprenorphine on fentanyl-induced respiratory depression were substantial and significant, so the authors regard these results as clinically relevant despite the small sample size, and valid from the perspective of a single-centre trial.

In conclusion, data from this study provide clinical evidence that buprenorphine reduces the harmful effects of fentanyl on ventilation and protects against fentanyl-induced respiratory depression in a concentration-dependent manner. Future research, including studies with larger sample sizes and combining other populations in clinical practice, designed to confirm the potential protective effect of buprenorphine against this fatal consequence of opioid misuse, is warranted.

## Supporting information

S1 Checklist CONSORT 2010 checklist of information to include when reporting a randomised trial*. (PDF)

S1 Fig Schematic representation of competitive binding at the MOR by fentanyl and buprenorphine, resulting in a ventilatory response. (TIF)

S2 Fig Individual V_E_ per concentration level. (PDF)

S1 File Clinical study protocol. (PDF)
