# Supplementary figures and images for: Effect of sustained high buprenorphine plasma concentrations on fentanyl-induced respiratory depression: A placebo-controlled crossover study in healthy volunteers and opioid-tolerant patients
Source: PLoS One. 2022 Jan 27;17(1):e0256752. doi: 10.1371/journal.pone.0256752 (PMC8794186; doi:10.1371/journal.pone.0256752)

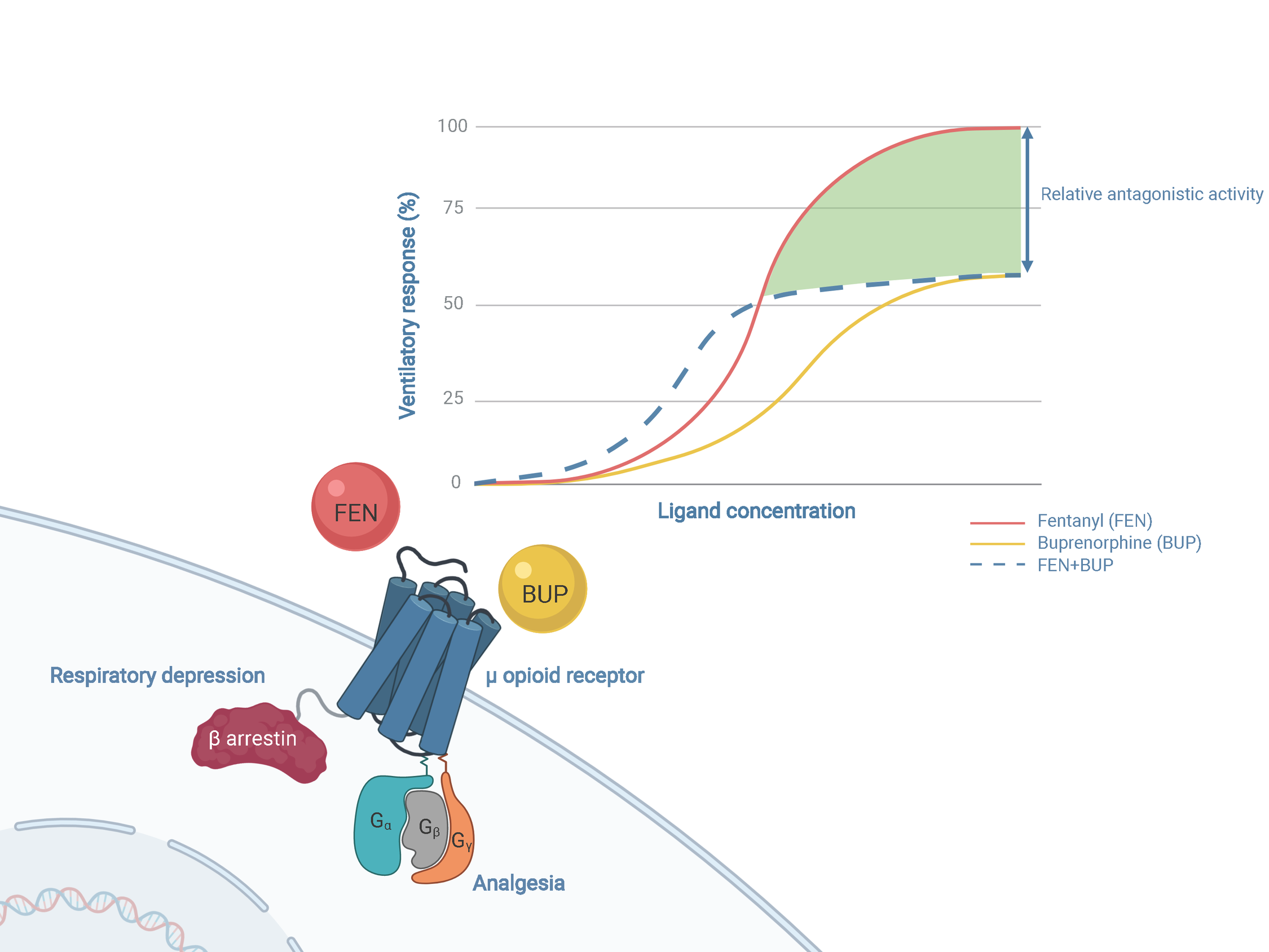

Supplement: S1 Fig — (TIF) [file pone.0256752.s002.tif]
