# Supplementary material for: Effect of sustained high buprenorphine plasma concentrations on fentanyl-induced respiratory depression: A placebo-controlled crossover study in healthy volunteers and opioid-tolerant patients
Source: PLoS One. 2022 Jan 27;17(1):e0256752. doi: 10.1371/journal.pone.0256752 (PMC8794186; doi:10.1371/journal.pone.0256752)

Supplemental Figures to the manuscript titled:

Effect of sustained high buprenorphine plasma concentrations on fentanyl-induced respiratory depression: a placebo-controlled crossover study in healthy volunteers and opioid-tolerant patients

Individual V_E per concentration level

Reader information:

The below graphs show adjudicated data. The data analysed and described in the manuscript regard adjudicated data based on clinical notes, as mentioned in the methods section of the main text.

Each colour represents a different participant. Vertical grey lines indicate the start of placebo/buprenorphine administration and the timing of the scheduled fentanyl boluses. The continuous coloured line of a particular participant transforms into a dotted line from the timepoint that no scheduled fentanyl bolus is administered, onwards. As detailed in the description of Fig 4 of the manuscript, there may be breaks in the continuous line of a particular subject due to brief periods of mask-removal for various reasons.

HEALTHY VOLUNTEERS

0.02mg/70kg/hr Placebo visit

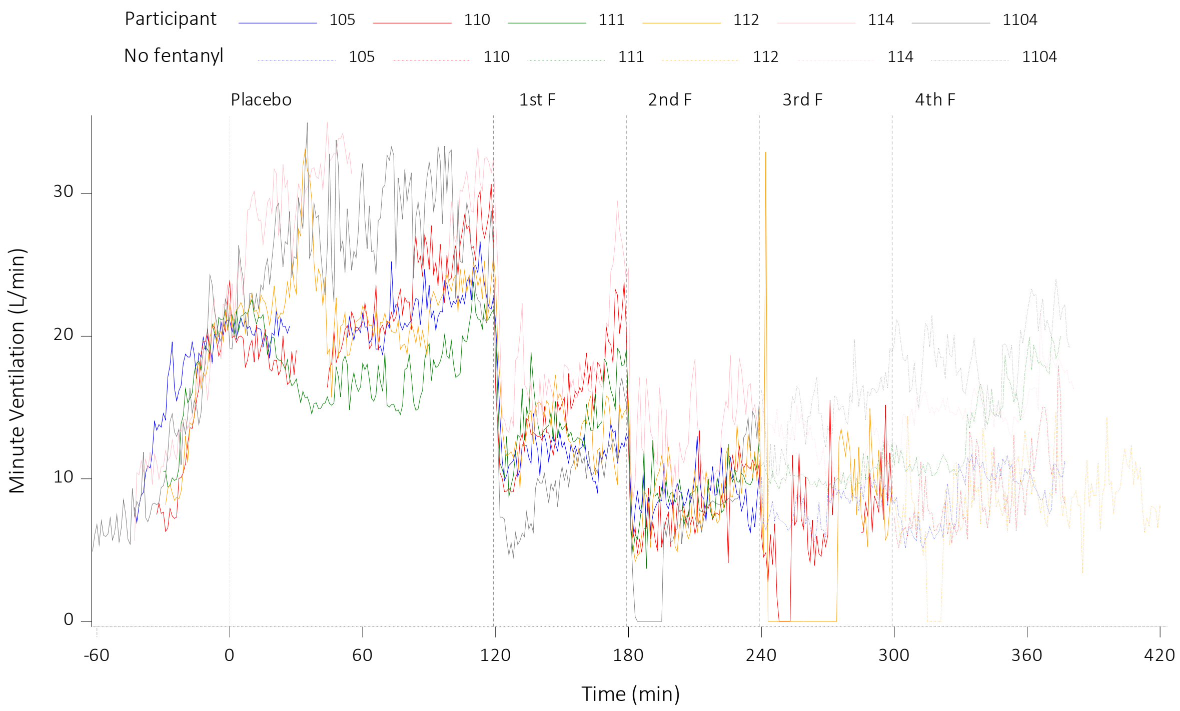

0.02mg/70kg/hr Buprenorphine visit

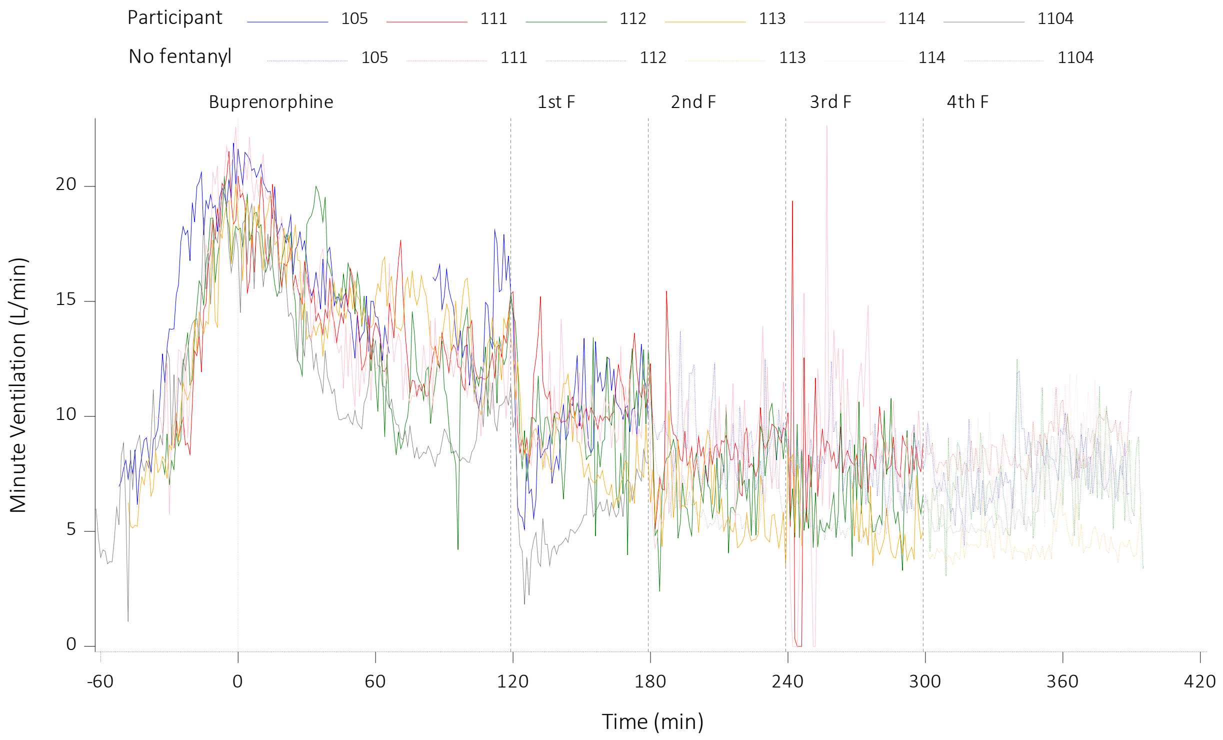

0.05mg/70kg/hr Placebo visit

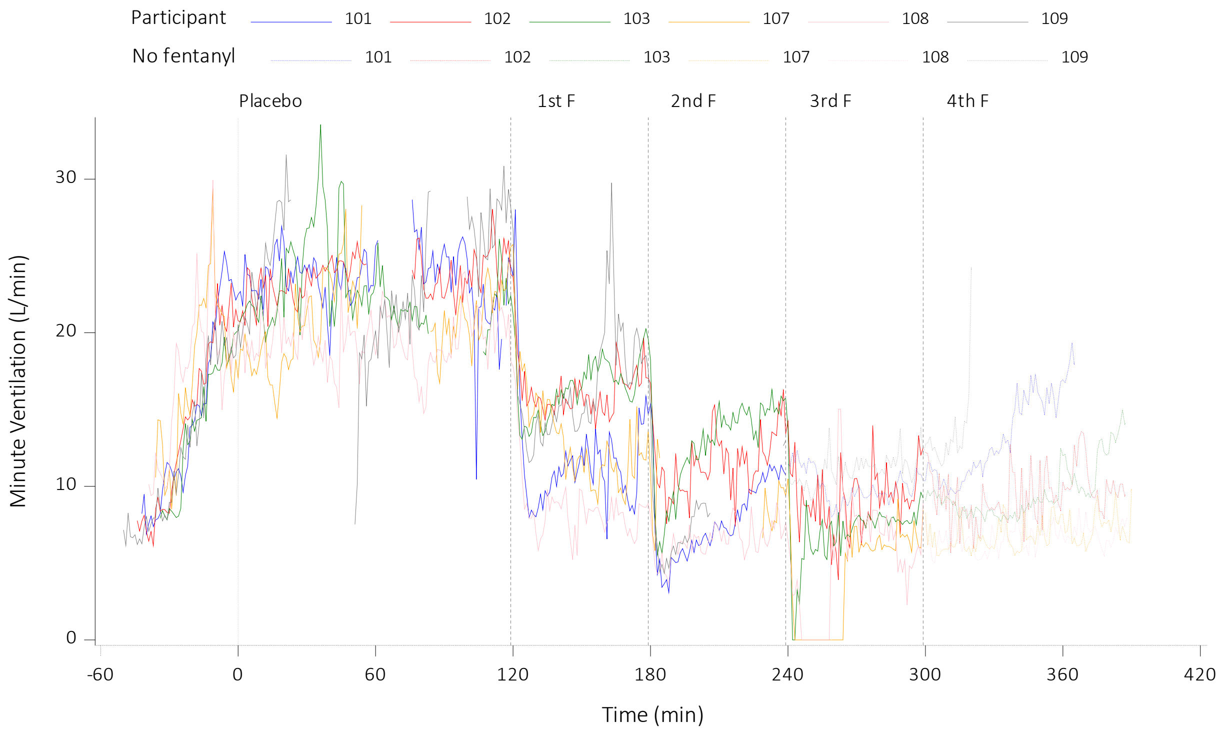

0.05mg/70kg/hr Buprenorphine visit

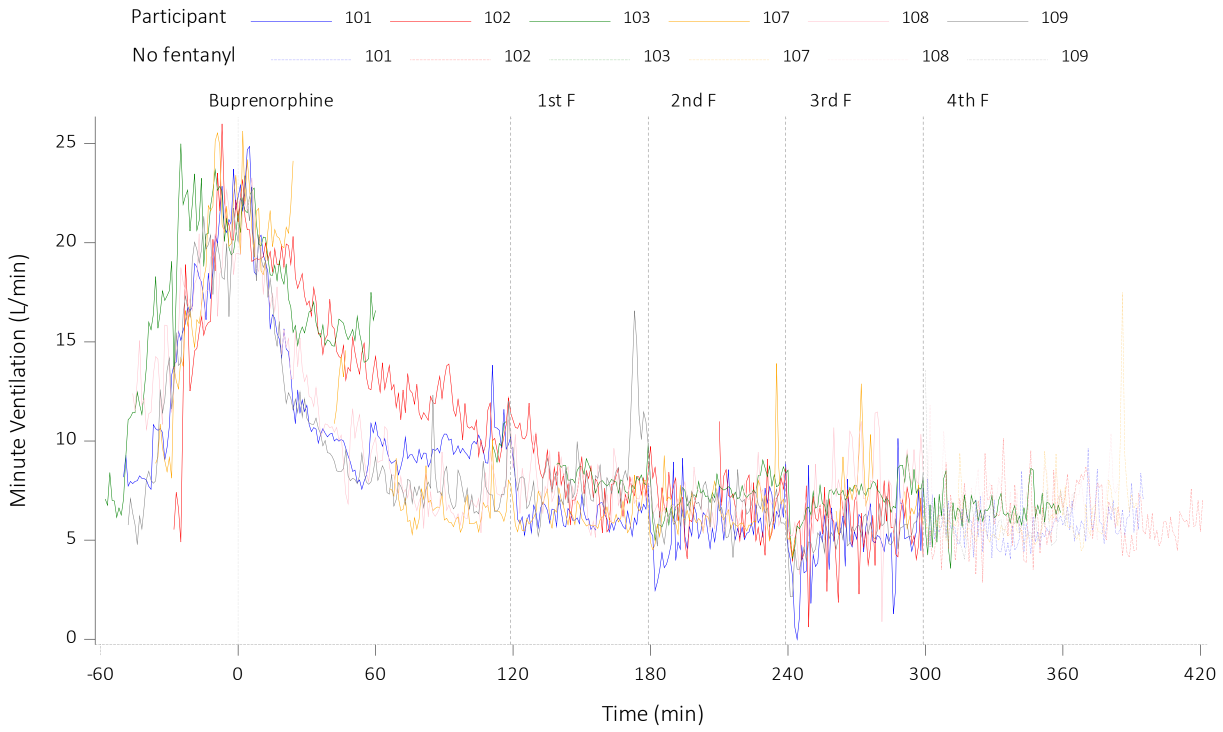

OPIOID TOLERANT PATIENTS

0.1mg/70kg/hr Placebo visit

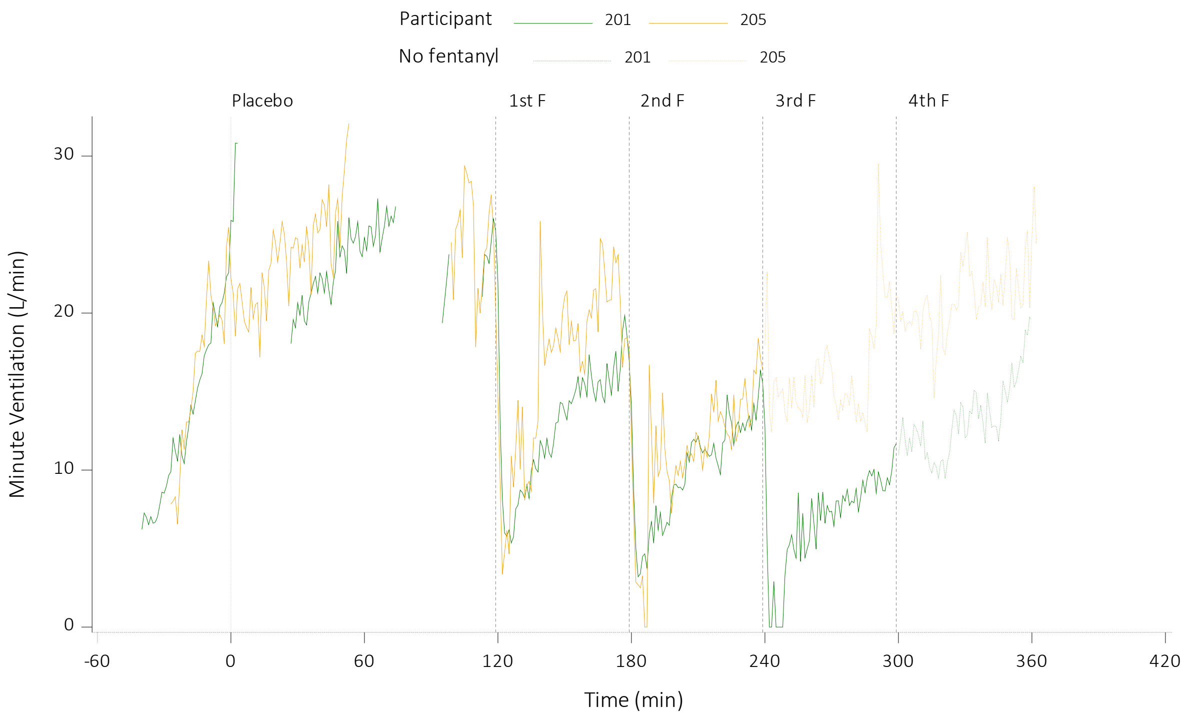

0.1mg/70kg/hr Buprenorphine visit

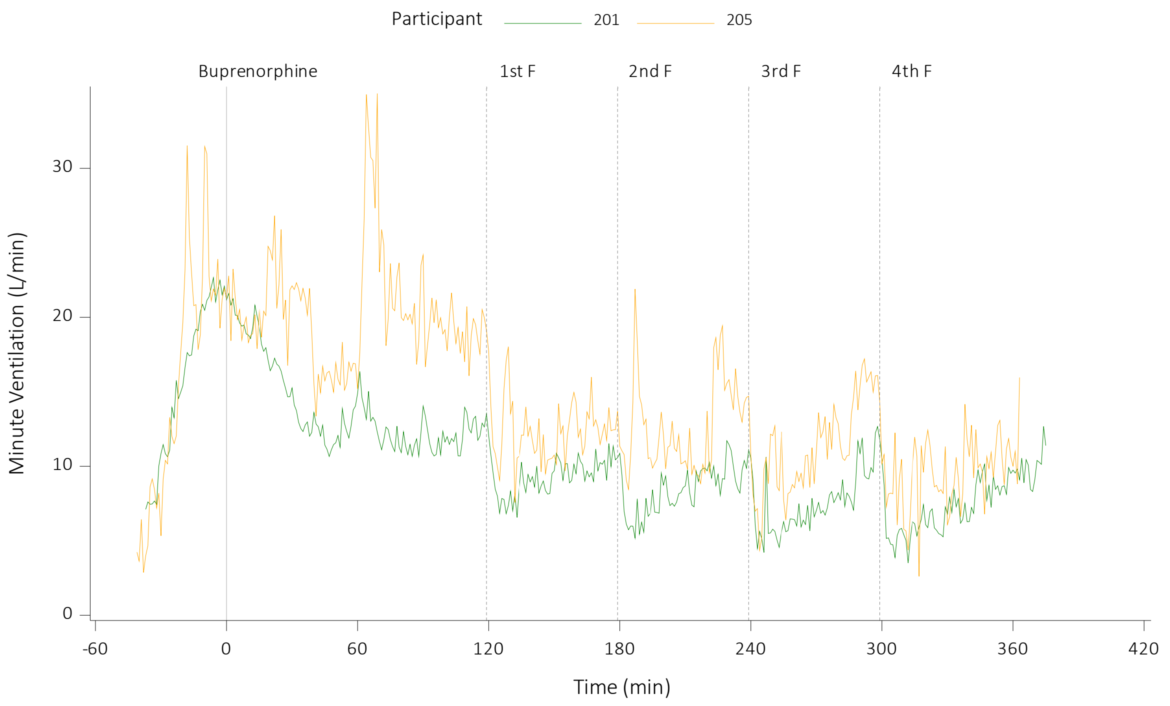

0.2mg/70kg/hr Placebo visit

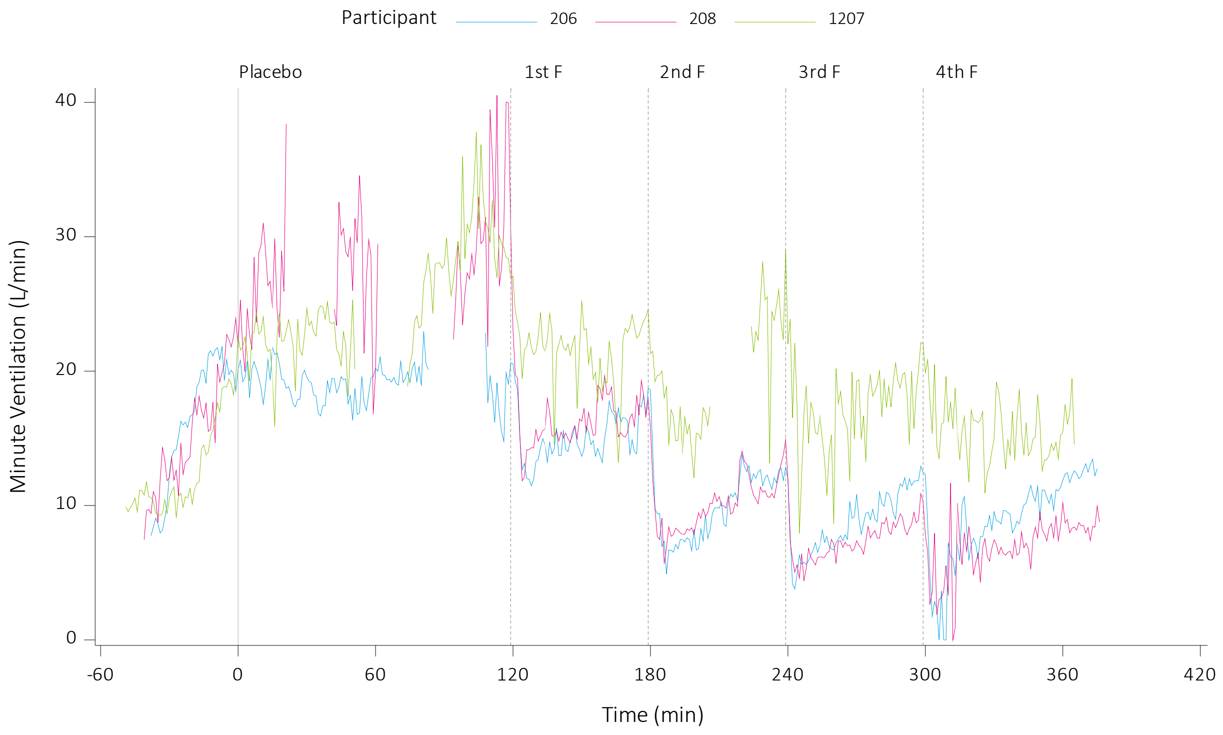

0.2mg/70kg/hr Buprenorphine visit

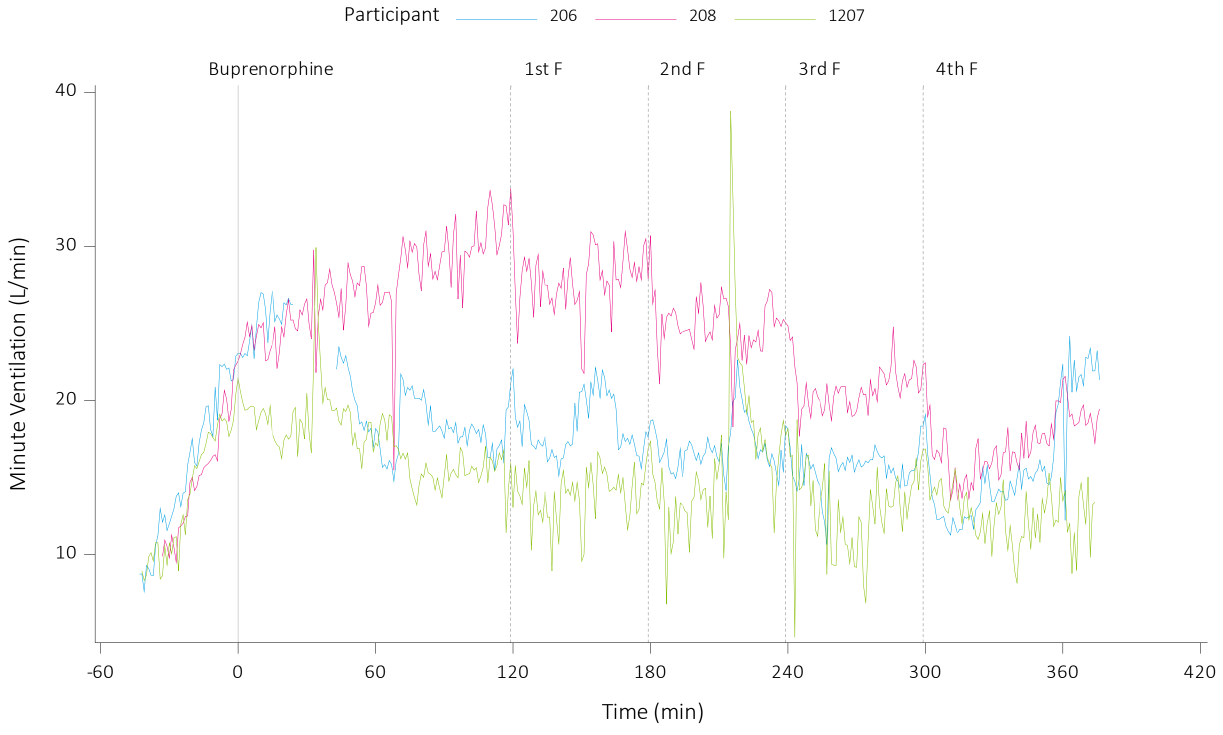

0.5mg/70kg/hr Placebo visit

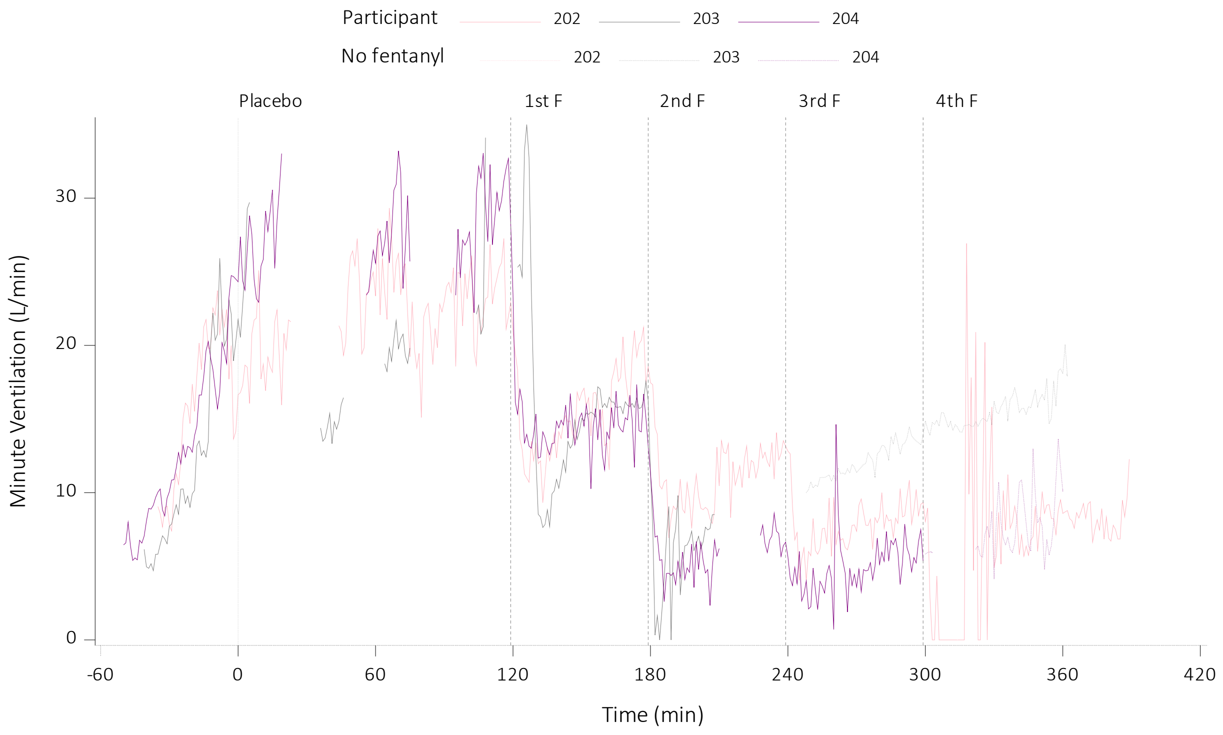

0.5mg/70kg/hr Buprenorphine visit

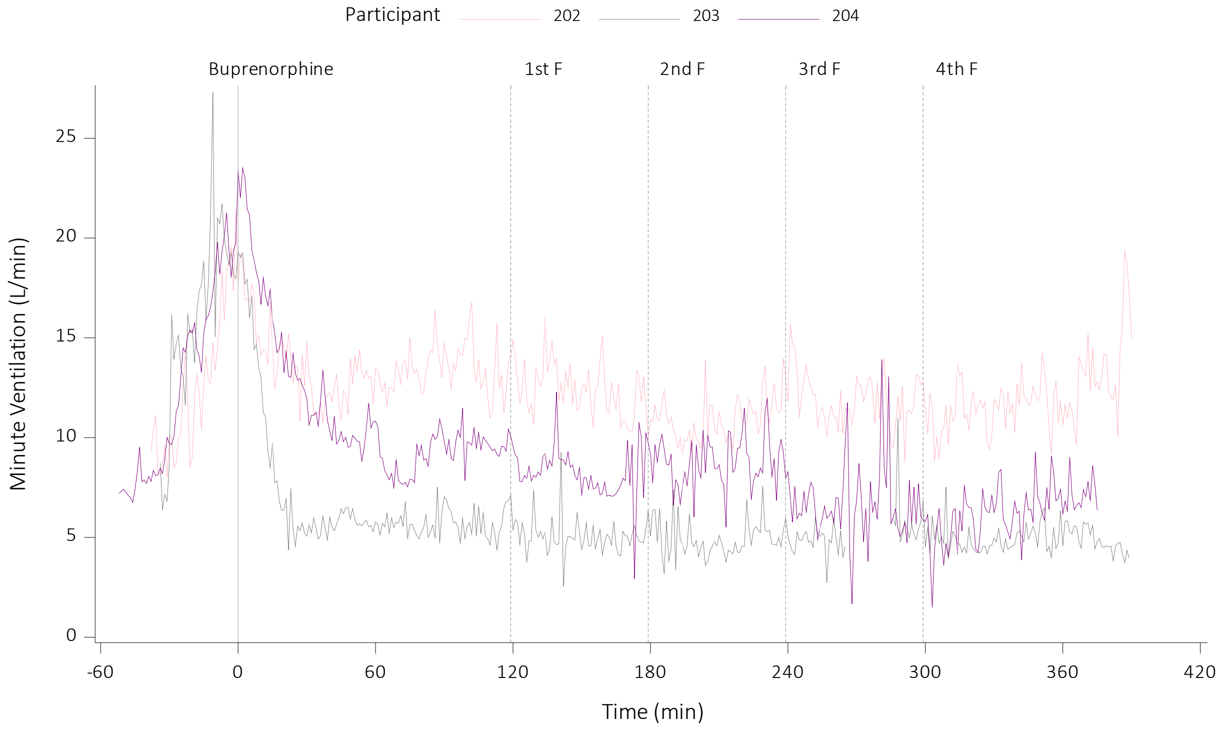

Supplement: S2 Fig — (PDF) [file pone.0256752.s003.pdf]
