# Supplementary material for: Effect of sustained high buprenorphine plasma concentrations on fentanyl-induced respiratory depression: A placebo-controlled crossover study in healthy volunteers and opioid-tolerant patients
Source: PLoS One. 2022 Jan 27;17(1):e0256752. doi: 10.1371/journal.pone.0256752 (PMC8794186; doi:10.1371/journal.pone.0256752)
Supplement: S1 File — (PDF) [file pone.0256752.s004.pdf]

[redacted]

CLINICAL STUDY PROTOCOL

A study examining the pharmacodynamic interaction between buprenorphine and fentanyl

Short Title:	Buprenorphine-Fentanyl Interaction Study
Version:	4
Original Protocol Date:	14-Feb-2018
Amendment Number:	3
Amendment Date:	12-Sep-2018
CHDR number:	CHDR1754
Sponsor number:	INDV-6000-101
Toetsing Online number:	NL64260.056.17
EudraCT number:	2017-004858-42

SUMMARY OF CHANGES

Study Title

A study examining the pharmacodynamic interaction between buprenorphine and fentanyl

The following revisions were made to the protocol, which are also reflected in the synopsis and other documents:

**PROTOCOL VERSION 2, AMENDMENT 1: 23-May-2018**			
**Change**	**Rationale**	**Justification & Classification**	**Changed Document(s), Section**
Addition of a buprenorphine-only dosing period to Part A of the study	To understand what portion of the respiratory depression observed during Periods 1 and 2 reflects buprenorphine-only or a cumulative effect of the fentanyl and buprenorphine.	Change in study design: Substantial	- Protocol, Section 1.3.3, 1.3.4, 1.3.7, 3, 3.1, 3.1.2, 5.4.1, 5.4.2, 7.3, 7.5
Protocol visit windows may be extended if pre-approved by the medical monitor, the principal investigator and co-investigator from the LUMC.	This permits flexibility for potential subject scheduling difficulties which may be experienced with the additional dosing period. Subjects will complete a full safety re-evaluation at admission; therefore, this is not a safety concern.	Change in visit window: Non-substantial	- Protocol, Section 1.3.7, 3.1
Change in study population to include a minimum equal number of female and male subjects in the study	The potential tolerability differences between males and females is yet unknown; therefore, both will be required.	Change in study design/study population: Substantial	- Protocol, Section 1.3.3, 9
Change in the required duration in which the subjects have to wear the ventilation mask	The ventilation mask was required to be worn up to 900 minutes, however, this is has shown to be unnecessary.	Change in study conduct: Non-substantial	- Protocol, Section 7.1.2
Safety measure for treatment of chest wall rigidity added to protocol	Chest wall rigidity is one of the most serious adverse events associated with fentanyl use and its onset is unpredictable. A protocol was put in place in case of this event.	Addition of safety measure: Non-substantial	- Protocol, Section 1.3.6
Increase in the upper limits of the systolic and diastolic blood pressure values considered to be an adverse event	This change was made to better reflect the range of systolic and diastolic blood pressure values expected during the study.	Change in definition of an adverse event: Non-substantial	- Protocol, Section 7.2.6

PROTOCOL VERSION 2, AMENDMENT 1: 23-May-2018

Change	Rationale	Justification & Classification	Changed Document(s), Section
The '900 minutes after dosing' time point in the table of assessments changed from a fixed time point into a flexible time point	The intent of the 900-minutes after dosing time point assessments were to ensure subjects are stable, however subjects may be already stable before this time.	Change in time point: Non-substantial	-Protocol, schedule of assessments.
Clarification on the timing of the sedation VAS	The timing of this assessment was not consistent throughout the protocol.	Change in time point: Non-substantial	- Protocol, Section 7.1.3, schedule of assessments
Change in definition of when a pregnancy outcome must be reported as an SAE	The original protocol inadvertently specified that spontaneous abortion should be reported as an SAE	Change in safety reporting: Substantial	- Protocol, Section 8.3.1
Change in inclusion criterion #5 for Part B (Opioid-tolerant patients)	An oral morphine equivalent daily dose of ≥ 90 mg was more appropriate to represent this population than the former ≥ 60 mg stated.	Change in study population: Substantial	- Protocol, Section 1.3.4, 3.1.1, 4.3,
Addition of venous pharmacokinetic (PK) samples for the buprenorphine-only part	Indivior has previously collected PK data for opioid-tolerant patients using venous plasma collection. In order to use this study's PK data with previously created PK/PD models, additional venous PK samples for buprenorphine will be collected in Part B.	Addition of subject burden: Substantial	- Protocol, Section 7.3, 7.5
Change of co-investigator	Substitution of a co-investigator for an absent co-investigator.	Administrative change: Non-substantial	-Protocol, contact details
General editing for grammar and typographical errors	Minor, non-content text changes were made throughout the protocol, which are not specifically noted.	Non-content text changes: Non-substantial	-Protocol, Sections 1-11

PROTOCOL VERSION 3, AMENDMENT 2: 19-Jul-2018			
**Change**	**Rationale**	**Justification & Classification**	**Changed Document(s), Section**
Change in visit schedule for Part B (Opioid-tolerant patients)	A longer wash-out period is required for several opioids with long half-lives. Patients will return to CHDR between study periods.	Change in visit schedule: Substantial	- Protocol, Section 1.3.3, 3.1, 4.4.3, 7.2.5, schedule of assessments
Removal of exclusion criterion #7 for Part B (Opioid-tolerant patients)	In/exclusion criteria updated to include a broader group of OT patients, including patients receiving IV opioid treatment	Change in study population: Substantial	- Protocol, Section 4.3, 4.4.2
Update of bridging medication	Two dosing periods per day is not sufficient for all bridging schedules, flexibility is needed to find an adequate scheme for each individual.	Change in visit schedule: Non-substantial	- Protocol, Section 4.4.3
Change in assessment schedule for Part B (Opioid-tolerant patients) for haematology, safety chemistry and measurement of weight	The results of the haematology and safety chemistry assessments for period 2 will be acquired before study drug administration.<br>The weight measured on Day -1 and Day 2 will be used for the dosage of study medication during Period 1 and Period 2, respectively.	Change in visit schedule: Substantial	- Protocol, Section 7.2.2, schedule of assessments
Correction in definition of when a apnoea must be recorded as an AE	The length of an apnoea that needs to be recorded as AE was inconsistent in the original protocol.	Change in safety assessment: Substantial	- Protocol, synopsis and section 1.3.6, 4.6.1, 7.1.2, 7.2.6, 8.1
Definition of a serious apnoeic event added	To ensure seriousness of apnoea is well defined.	Change in safety assessment: Substantial	- Protocol, Section 8.1.4
General editing for grammar and typographical errors	Minor, non-content text changes were made throughout the protocol.	Non-content text changes: Non-substantial	-Protocol, Sections 9.5, list of abbreviations

PROTOCOL VERSION 4, AMENDMENT 3: 12-Sept-2018			
**Change**	**Rationale**	**Justification & Classification**	**Changed Document(s), Section**
Update of inclusion criterion #7 for Part B (Opioid-tolerant patients)	Certain CNS-depressants can be safely administered in combination with the study drug.	Change in study population: Substantial	- Protocol, Section 4.2, 4.3 and 4.4.2
Update of exclusion criterion #1 for Part B (Opioid-tolerant patients)	Exclusion criterion updated to include a broader group of OT patients, if assessed as safe by the principal investigator.	Change in study population: Substantial	- Protocol, Section 4.2 and 4.3
Update of exclusion criterion #4 for Part B (Opioid-tolerant patients)	The impact of smoking on the respiratory measurements is not as significant as previously thought.	Change in study population: Substantial	- Protocol, Section 4.2, 4.3 and 4.4.2

CONTACT DETAILS

**SPONSOR**	Indivior UK Ltd<br>[redacted]
Medical Monitor	[redacted]
Back-up Medical Monitor	[redacted]
Senior Vice President, Global Medicines Development	[redacted]
**Trial Site 1**	Centre for Human Drug Research<br>[redacted]
Principal Investigator	[redacted]
Co-investigator	[redacted]
Co-investigator	[redacted]
Co-investigator	[redacted]
Manager Operations Unit	[redacted]
Manager Clinical Unit	[redacted]
Statistician	[redacted]

**Trial Site 2**	Leiden University Medical Centre<br>[redacted]
Contact person	[redacted]
**INDEPENDENT PHYSICIAN**	[redacted]
**LABORATORY – CHEMISTRY AND HAEMATOLOGY**	[redacted]<br>AKCL LUMC, [redacted]
Contact person	[redacted]
**LABORATORY – MICROBIOLOGY**	[redacted]<br>KML LUMC, [redacted]
Contact person	[redacted]
**LABORATORY – PK BIOANALYTICAL**	Keystone Bioanalytical Inc.<br>[redacted]
Contact person	[redacted]
**DATA MANAGEMENT AND STATISTICAL PROGRAMMING**	eClinical Solutions<br>[redacted]
Contact person	[redacted]
**PHARMACY**<br>**(visit & delivery address)**	Apotheek LUMC, L0-P30<br>[redacted]
Pharmacist/Trial Manager	[redacted]

SIGNATURE PAGE - PRINCIPAL INVESTIGATOR

Study Title

A study examining the pharmacodynamic interaction between buprenorphine and fentanyl

I acknowledge accountability for this protocol in accordance with CHDR's current procedures.

[redacted]

SIGNATURE PAGE - TRIAL SITE STAFF
Centre for Human Drug Research

Study Title
A study examining the pharmacodynamic interaction between buprenorphine and fentanyl

I acknowledge responsibility for this protocol in accordance with CHDR's current procedures.

[redacted]

SIGNATURE PAGE - SPONSOR

Indivior UK Ltd.

Study Title

A study examining the pharmacodynamic interaction between buprenorphine and fentanyl

I approve this protocol on behalf of the sponsor.

[redacted]

TABLE OF CONTENTS

SUMMARY OF CHANGES 2
CONTACT DETAILS 6
SIGNATURE PAGE - PRINCIPAL INVESTIGATOR 8
SIGNATURE PAGE - TRIAL SITE STAFF 9
SIGNATURE PAGE - SPONSOR 10
TABLE OF CONTENTS 11
LIST OF ABBREVIATIONS 16
PROTOCOL SYNOPSIS 19
Primary Endpoints: 27
Secondary Endpoints 27
Exploratory Endpoints (Safety and tolerability) 27
Exploratory Endpoints (Pharmacokinetic) 27
Exploratory endpoints (Pharmacodynamic) 28
TABLE 1: SCHEDULE OF ASSESSMENTS – PART A OVERVIEW OF STUDY 29
TABLE 2: SCHEDULE OF ASSESSMENTS – PART B OVERVIEW OF STUDY 31
TABLE 3: SCHEDULE OF ASSESSMENTS – PART A PERIOD ASSESSMENTS AT LUMC 33
TABLE 4: SCHEDULE OF ASSESSMENTS – PART B PERIOD ASSESSMENTS AT LUMC 35
1 BACKGROUND AND RATIONALE 37
1.1 Context 37
1.2 Clinical pharmacology information 37
1.3 Study rationale 38
1.3.1 Benefit and risk assessment 38
1.3.2 Medical and regulatory background 38
1.3.3 Study population 39
1.3.4 Study design 40
1.3.5 Comparative drug(s) and/or placebo 40
1.3.6 Dose rationale, dose determination and stopping criteria 40
1.3.7 Treatment duration 42
2 STUDY OBJECTIVES 43
2.1 Primary objective 43
2.2 Secondary objective 43
2.3 Exploratory objectives 43
3 STUDY DESIGN 44

3.1 Overall study design and plan ... 44
3.1.1 Screening (Location: CHDR) ... 45
3.1.2 Treatment and observation period (Location: CHDR/LUMC) ... 46
3.1.3 End of Study/Early Termination Visit (Location: CHDR) ... 48
4 STUDY POPULATION ... 49
4.1 Subject population ... 49
4.2 Inclusion criteria ... 49
4.3 Exclusion criteria ... 49
4.4 Concomitant medications ... 51
4.4.1 Allowed concomitant medications ... 51
4.4.2 Prohibited concomitant medications ... 51
4.4.3 Bridging medication ... 52
4.5 Lifestyle restrictions ... 52
4.5.1 Contraception requirements ... 53
4.6 Study drug discontinuation and withdrawal ... 53
4.6.1 Study drug interruption or discontinuation ... 53
4.6.2 Subject withdrawal ... 53
4.6.3 Replacement policy ... 54
5 INVESTIGATIONAL MEDICINAL PRODUCT ... 55
5.1 Investigational drug and matching placebo ... 55
5.2 Study drug packaging and labelling ... 55
5.3 Drug accountability ... 55
5.4 Treatment assignment and blinding ... 55
5.4.1 Randomisation and treatment assignment ... 55
5.4.2 Blinding ... 56
6 STUDY ENDPOINTS ... 57
6.1 Primary endpoint ... 57
6.2 Secondary endpoints ... 57
6.3 Exploratory endpoints ... 57
6.3.1 Safety and tolerability ... 57
6.3.2 Pharmacokinetic ... 58
6.3.3 Pharmacodynamic ... 58
7 STUDY ASSESSMENTS ... 60
7.1 Pharmacodynamic assessments and questionnaires ... 60

7.1.1 Concomitant medications 60
7.1.2 Ventilation Monitoring 60
7.1.3 Sedation Visual Analog Scale 61
7.2 Safety and tolerability assessments 61
7.2.1 Vital signs 61
7.2.2 Weight and height 61
7.2.3 Physical examination 61
7.2.4 Standard 12-lead electrocardiograms 61
7.2.5 Columbia Suicide Severity Rating Scale (C-SSRS) 62
7.2.6 Continuous monitoring 62
7.2.7 Laboratory assessments 62
7.3 Pharmacokinetic assessments 64
7.3.1 Labelling 65
7.3.2 Shipping Procedures 65
7.4 Sequence of assessments and time windows 65
7.5 Total blood volume 66
8 SAFETY REPORTING 67
8.1 Definitions of adverse events 67
8.1.1 Intensity of adverse events 67
8.1.2 Relationship to study drug 67
8.1.3 Action 67
8.1.4 Serious adverse events 67
8.1.5 Suspected unexpected serious adverse reactions 68
8.1.6 Reporting of serious adverse events 68
8.1.7 Follow-up of adverse events 69
8.2 Temporary halt for reasons of subject safety 69
8.3 Pregnancy 69
8.3.1 Reporting of pregnancy 69
9 STATISTICAL METHODOLOGY AND ANALYSES 70
9.1 Statistical analysis plan 70
9.2 Power calculation 70
9.3 Missing, unused and spurious data 70
9.4 Analysis sets 70
9.4.1 Safety set 70

9.4.2 Pharmacokinetic analysis set ... 71
9.4.3 Pharmacodynamic analysis set ... 71
9.5 Subject disposition ... 71
9.6 Baseline parameters and concomitant medications ... 71
9.6.1 Demographics and baseline variables ... 71
9.6.2 Medical history ... 71
9.6.3 Concomitant Medications ... 71
9.6.4 Treatment compliance/exposure ... 71
9.7 Safety and tolerability endpoints ... 72
9.7.1 Adverse events ... 72
9.7.2 Vital signs ... 72
9.7.3 ECG ... 72
9.7.4 Clinical laboratory tests ... 72
9.8 Pharmacokinetic and pharmacodynamic endpoints ... 72
9.8.1 Pharmacokinetics ... 72
9.8.2 Pharmacodynamics ... 73
9.8.3 Inferential methods ... 73
9.8.4 Pharmacokinetic/Pharmacodynamic modelling ... 73
9.9 Exploratory analyses and deviations ... 74
9.10 Interim analyses ... 74
10 GOOD CLINICAL PRACTICE, ETHICS AND ADMINISTRATIVE PROCEDURES ... 75
10.1 Good clinical practice ... 75
10.1.1 Ethics and good clinical practice ... 75
10.1.2 Ethics committee ... 75
10.1.3 Informed consent ... 75
10.1.4 Insurance ... 75
10.2 Study funding ... 75
10.3 Data handling and record keeping ... 76
10.3.1 Source data collection ... 76
10.3.2 Database management ... 76
10.4 Access to source data and documents ... 77
10.5 Protocol deviations ... 77
10.6 Quality control and quality assurance ... 77
10.6.1 Monitoring ... 77

10.7 Protocol amendments 77
10.7.1 Substantial amendment 77
10.7.2 Non-substantial amendment 78
10.7.3 Urgent amendment 78
10.8 End of study report 78
10.9 Public disclosure and publication policy 79
11 REFERENCES 80
APPENDIX A: MORPHINE EQUIVALENT DOSING FOR ORAL OPIOID DRUGS 82
APPENDIX B: COLUMBIA SUICIDE SEVERITY RATING SCALE (C-SSRS) - SCREENING/BASELINE 84
APPENDIX C: COLUMBIA SUICIDE SEVERITY RATING SCALE (C-SSRS) – SINCE LAST VISIT 86
APPENDIX D: SEDATION VISUAL ANALOG SCALE 88

LIST OF ABBREVIATIONS

AE	Adverse event
ABR	ABR form, General Assessment and Registration form, is the application form that is required for submission to the accredited Ethics Committee; in Dutch, ABR = Algemene Beoordeling en Registratie
AKCL	Clinical Chemistry Laboratory at Leiden University Medical Centre
ALT	Alanine aminotransferase (ALT)/serum glutamic pyruvic transaminase (SGPT)
AST	Aspartate aminotransferase (AST)/serum glutamic oxaloacetic transaminase (SGOT)
AUC	Area under the curve
BID	Bis in diem/twice a day
BLQ	Below limit of quantification
BMI	Body mass index
bpm	Beats per minute
CA	Competent authority (also CCMO)
C_{avg}	Average plasma concentration
CHDR	Centre for Human Drug Research
CKD-EPI	Chronic Kidney Disease Epidemiology Collaboration
CL/F	Apparent clearance
C_{max}	Maximum plasma concentration
CNS	Central nervous system
CRF	Case Report Form
C-SSRS	Columbia Suicide Severity Rating Scale
DEF	Dynamic end-tidal forcing
DSM	Diagnostic and Statistical Manual of Mental Disorders
EC	Ethics Committee (also Medical Research Ethics Committee [MREC]); in Dutch: Medisch Ethische Toetsing Commissie (METC)
EC_{50}	Concentration yielding 50% of maximum effect
ECG	Electrocardiogram
EDC	Electronic data capture
EDTA	Ethylenediaminetetraacetic acid
EENT	Eyes/ears/nose/throat
E_{max}	Maximum effect
EOS	End of Study
ET	Early termination
EU	European Union
EudraCT	European Clinical Trials Database
FSH	Follicle stimulating hormone
GCP	Good Clinical Practice
HBsAg	Hepatitis B surface antigen
HepC	Hepatitis C
HR	Heart rate
ICF	Informed consent form

ICH	International Conference on Harmonisation
IM	Intramuscular(ly)
IMP	Investigational medicinal product
INR	International Normalised Ratio
IV	Intravenous(ly)
LUMC	Leiden University Medical Centre
MAP	Mean arterial pressure
MAT	Medication-assisted therapy
MDMA	3,4-methylenedioxymethamphetamine
MedDRA	Medical Dictionary for Regulatory Activities
MOR	μ (mu) opioid receptor
OT	Opioid tolerant
OTC	Over-the-counter
OUD	Opioid use disorder
PACU	Post-Anaesthesia Care Unit
PAP	Pharmacometric analysis plan
PCO_2	Partial pressure of carbon dioxide
PD	Pharmacodynamic(s)
PK	Pharmacokinetic(s)
PO_2	Partial pressure of oxygen
PR	Pulse rate
PT	Prothrombin time
PV	Pharmacovigilance
QTc/QTcF	Corrected QT interval/ corrected QT interval (Fridericia's)
SAE	Serious adverse event
SAP	Statistical analysis plan
SD	Standard deviation
SE	Standard error
SEM	Standard error of the mean
SOC	System Organ Class
SOP	Standard Operating Procedure
SpO_2	Oxygen saturation
SST	Serum Separator Tube
SUSAR	Suspected unexpected serious adverse reaction
TEAE	Treatment-emergent adverse event
T_{max}	Time to maximum plasma concentration
TSH	Thyroid stimulating hormone
VAS	Visual Analogue Scale
V_E	Minute ventilation
Vz/F	Apparent clearance
UDS	Urine drug screen

ULN	Upper limit of normal
USA	United States of America
WHODD	World Health Organisation Drug Dictionary
WMO	Medical Research Involving Human Subjects Act; in Dutch: Wet Medisch-wetenschappelijk Onderzoek met Mensen

PROTOCOL SYNOPSIS

Sponsor:
Indivior UK Limited

Name of Investigational Medicinal Products:
Buprenorphine hydrochloride
Fentanyl citrate

Name of Active Ingredients:
Buprenorphine hydrochloride: (2S)-2-[17-Cyclopropylmethyl-4,5α-epoxy-3-hydroxy-6-methoxy-6α,14-ethano-14α-morphinan-7α-yl]-3,3-dimethylbutan-2-ol hydrochloride
Fentanyl citrate: N-(1-phenethyl-4-piperidyl) propionanilide citrate (1:1)

Protocol Title:
A study examining the pharmacodynamic interaction between buprenorphine and fentanyl

Short Title:
Buprenorphine-Fentanyl Interaction Study

Protocol Number:
INDV-6000-101

Protocol Phase:
Phase 1

Background and Rationale:

According to the 2016 National Survey on Drug Use and Health, in the United States of America (USA) there were 20.1 million people 12 years or older who suffered from substance use disorder [1], but many were not aware that addiction is a disease that can be medically treated [2]. The goals of medication-assisted treatment (MAT) are to reduce substance use and risk of relapse or overdose, to reduce harm from sequelae of substance abuse and to help patients return to a healthy, functional life [3].

In 2015 alone, the harm caused by drug use translated into an estimated 28 million years of healthy life lost worldwide due to premature death and disability [4]. In the USA, the misuse of opioids accounted for approximately 25% of the estimated number of drug-related deaths worldwide, and drug-related lethal overdoses increased from 16,849 to 52,404 annually during the 1999-2015 period [5, 6]. In Europe, the use of opioid analgesics has also increased, but this change has not been associated with a marked increase in overdose deaths [7]. Still, some regions have reported dramatic increases in overdose deaths and the common thread among these reports has been widespread access to highly potent fentanyl and carfentanil [8 - 11]. The risk of overdose deaths is particularly high for problem drug users after periods of relative abstinence, most notably soon after prison release, but also after hospital discharge. The most probable reason is loss of tolerance to the effects of opioids [8], but the changes in tolerance have not been well-characterised.

Buprenorphine, a partial agonist at the μ-opioid receptor (MOR) is used for the MAT of opioid use disorder (OUD). Buprenorphine has high affinity for the MOR and therapeutic plasma concentrations achieve ≥ 70% receptor occupancy. As a partial agonist, buprenorphine has a ceiling effect on respiratory depression such that it does not cause apnoea when administered alone and minute ventilation is not suppressed beyond 50 to 60% [12]. It is hypothesised that buprenorphine will competitively inhibit the effects of potent, short-acting MOR agonists like fentanyl and carfentanil that can result in apnoea and death. The objective of this trial is to determine if buprenorphine action at the MOR can shift the respiratory depression response to intravenous

(IV) fentanyl injection to the right, thereby reducing the potential of fentanyl to cause respiratory depression, which is the usual fatal precipitant associated with IV fentanyl/heroin overdose.

Objective(s):

Primary Objectives:

- To determine if buprenorphine action at the MOR can inhibit the respiratory depression response to IV fentanyl injection in healthy subjects and opioid-tolerant (OT) patients;
- To determine if therapeutic concentrations achieved with administration of buprenorphine in OT patients protect against respiratory depression associated with high concentrations of fentanyl.

Secondary Objectives:

- To assess safety as determined by adverse event (AE) reporting.

Exploratory Objectives:

- To assess safety as determined by concomitant medications, laboratory test results, vital signs, physical examination findings, ECG parameters and Columbia-Suicide Severity Rating Scale (C-SSRS) responses;
- To evaluate pharmacokinetics of buprenorphine during primed-continuous IV infusion;
- To evaluate pharmacokinetics of fentanyl with repeated IV bolus injections;
- To explore changes in ventilation parameters during the IV administration of buprenorphine and fentanyl; and
- To build a model describing the pharmacokinetic (PK)/pharmacodynamic (PD) interaction between buprenorphine and fentanyl concentrations and their effect on ventilatory parameters.

Design:

This study will be performed in 2 parts (Part A and Part B) at 2 clinical study sites. Dosing day procedures will be performed at Leiden University Medical Centre (LUMC); all other study procedures will be performed at the Centre for Human Drug Research (CHDR).

Part A is a 3-period study in approximately 18 healthy subjects. A minimum of 5 subjects of each sex will be included to address any potential differences between sexes. The first 2 periods (Period 1 and Period 2) have a single-blind, placebo-controlled, cross-over design, where subjects will be randomised in a 1 to 1 ratio to 2 treatment sequences determined by the order in which they receive buprenorphine or matching placebo. Period 3 is an open-label design, where the same subjects will receive buprenorphine only. Period 3 is optional, and not all subjects are required to participate; the sponsor and Investigator collectively determine if Period 3 is required for each subject. In total, about 6 subjects will participate in a third investigational period.

Per 3 subjects, the buprenorphine dose will be determined based on safety and pharmacodynamic results, leading to approximately 6 dose cohorts and a minimum of 3 subjects assigned to any buprenorphine dose level. Every effort will be made to represent both sexes in each buprenorphine dose level.

Part B is an open label study in approximately 8 OT patients. All OT patients will receive placebo plus fentanyl during Period 1 and buprenorphine plus fentanyl during Period 2. A minimum of 3 subjects of each sex will be included in the population in order to address any potential differences due to sex.

Healthy subjects' (Part A) participation is a maximum of 13 weeks; OT patients' (Part B) participation is approximately 8 weeks.

All healthy subjects (Part A) and OT patients (Part B) will be screened at CHDR up to 31 days prior to study drug administration. Subjects who sign informed consent and meet all entry criteria may be enrolled.

All healthy subjects in Part A will be studied in 2 or 3 periods, with 10-17 days between the periods. All subjects will receive ondansetron before dosing with buprenorphine or placebo in order to minimise the nausea effect of opioids. Subjects will receive the same doses of fentanyl challenges in Periods 1 and 2; however, fentanyl will not be dosed in Period 3. Buprenorphine doses will be set per dosing cohort. All subjects will be admitted to the CHDR the evening before each study period, and they will be escorted to LUMC the following morning. After study periods are completed, subjects will be transferred to the Post-Anaesthesia Care Unit (PACU) for overnight monitoring.

Part A: Overview of study design in healthy subjects

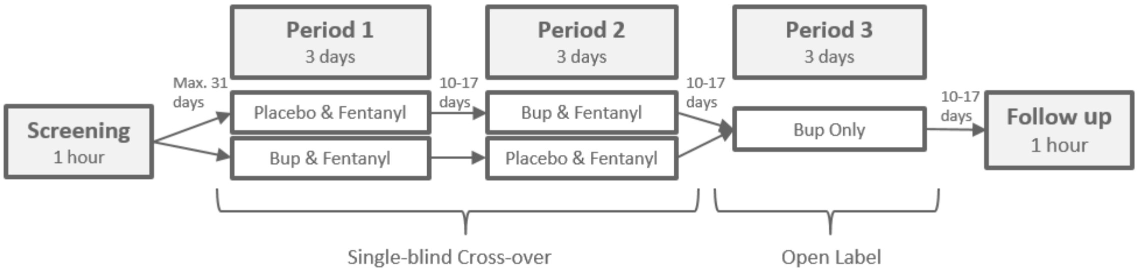

All OT patients in Part B will be studied in 2 separate periods, with at least 40 hours washout in between, while returning to CHDR between the two periods. All OT patients will be transitioned to oral oxycodone before Period 1, and they will be admitted to the CHDR 2 – 5 days before Period 1 in order to ensure wash-out of the patients' usual opioids and a stable dose with an adequate bridging schedule has been reached. Tolerance to opioid effects is poorly characterised in subjects receiving long-term opioids; therefore, OT patients in Part B will receive placebo plus fentanyl challenges in Period 1 in order to optimise the fentanyl dose escalation before buprenorphine and fentanyl are co-administered in Period 2. Due to the short half-life of fentanyl, OT patients will return to CHDR and will continue onto Period 2 after a washout of at least 40 hours. Opioid-tolerant patients will be escorted to LUMC on the morning of Day 1 and Day 3. In the event that a washout period significantly longer than 40 hours is required between Periods 1 and 2, OT patients may stay at CHDR for a longer period until Period 2 begins.

Part B: Overview of study design in opioid-tolerant patients

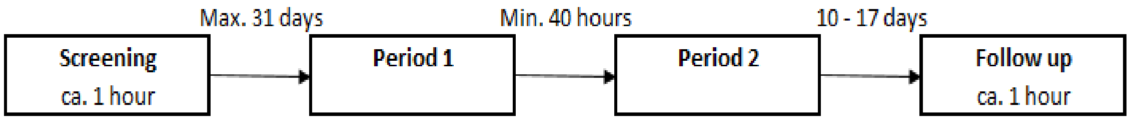

The End of Study/Early Termination (EOS/ET) visit will be completed 10-17 days after the final study period dosing.

To study ventilation, the dynamic end-tidal forcing technique will be used [12, 13]. This technique enables the Investigator to force end-tidal PCO_2 and end-tidal PO_2 to follow a specific pattern in time. End-tidal PCO_2

and PO_2 will be clamped to approximately 7 and 14.5 kPa, respectively, until minute ventilation (VE) reaches 20 to 24 L/min. Subjects breathe through a face mask and receive fresh gas (45 L/min) with oxygen, carbon dioxide and nitrogen adjusted to obtain the desired end-tidal concentrations. The inspired and expired gas flows are measured using a pneumotachograph and the oxygen and carbon dioxide concentrations are measured using a gas monitor; a pulse oximeter continuously measures the oxygen saturation of arterial haemoglobin with a finger probe. All relevant variables are available for online analysis and stored on a breath-to-breath basis for further analysis.

After baseline ventilation stabilises (30 to 45 minutes), subjects (all healthy subjects and OT patients as needed) will receive ondansetron 4 mg IV and a primed-continuous IV buprenorphine (or placebo) infusion will be initiated at doses expected to achieve target concentrations resulting in approximately 25% to 50% suppression of baseline minute ventilation. Buprenorphine infusion will continue for 6 hours (360 minutes) and fentanyl boluses will be administered at 120, 180, 240 and 300 minutes (in Periods 1 and 2) to complete a 4-step IV bolus dose escalation. Study drug administration concludes at 360 minutes, and study subjects will be monitored for a minimum of 3 hours before transfer to the PACU. If a subject indicates that he or she wants to discontinue the experiment or in case of an AE, all infusions will be discontinued for that period. Subjects with a procedure-related AE will be treated according to established ventilatory support and opioid reversal protocols. A procedure-related AE is defined by loss of respiratory activity for 60 seconds or longer, despite active stimulation of the subject; end-tidal partial pressure of carbon dioxide greater than 67.5 mmHg, O_2 saturation less than 85% for at least 2 minutes, or any other situation or condition that may interfere with the health of the participant. If an Investigator stimulates a subject to breathe or gives supplemental oxygen as needed to prevent an AE, the subject will not proceed to the next fentanyl dose and the period will terminate early.

Investigational Drug:

- Buprenorphine (Temgesic®) 0.3 mg/mL for intravenous injection
- Fentanyl 0.05 mg/mL for intravenous injection
- Placebo 0.9% normal saline for intravenous injection

Non-Investigational Drug:

- Ondansetron 2 mg/mL for intravenous injection
- Oxycodone tablets (for Part B only)

<u>Dose and Duration of Treatment:</u>

Part A: Healthy subjects

All healthy subjects will be dosed with 4 mg ondansetron at least 15 minutes before dosing with buprenorphine or placebo. Buprenorphine dosing is flexible and infusion rates will be selected to target approximately 25 to 50% respiratory depression. The potential buprenorphine continuous infusion rates, based on published reports providing a dose-response for buprenorphine effects on analgesia and ventilation [12, 13, 27], include 0.005, 0.01, 0.02, 0.05, 0.1 mg and 0.2 mg/70 kg/h. The initial dose cohort of 3 subjects will receive the starting dose of buprenorphine listed below and subsequent doses will be selected to explore the full range of effects on ventilation. The starting dose of buprenorphine is expected to yield significant receptor occupancy and produce differential effects on respiratory depression relative to placebo. Doses for subsequent dose cohorts will be selected from the potential doses listed based on subject tolerability and respiratory effects from earlier dose cohorts.

Starting buprenorphine dose: target concentration 0.5 ng/mL

- 0.125 mg/70 kg bolus
- 0.05 mg/70 kg/h for 360 minutes

Subjects will be dosed with 90-second bolus injections of fentanyl using the following escalation:

- Bolus 1: 0.075 mg/70 kg
- Bolus 2: 0.15 mg/70 kg (hold if apnoea observed in earlier steps)
- Bolus 3: 0.25 mg/70 kg (hold if apnoea observed in earlier steps)
- Bolus 4: 0.35 mg/70 kg (hold if apnoea observed in earlier steps)

Apnoea is defined as a 20-second respiratory pause.

Part B: Opioid-tolerant patients

Opioid-tolerant patients in Part B will undergo a washout of their own opioids during which time these will be replaced by oral oxycodone; they will continue to receive stable doses of oxycodone from 2 – 5 days before Period 1 until discharge at the end of Period 2. Not less than 15 hours prior to the start of each dose administration of buprenorphine/placebo, the last oxycodone dose will be administered.

Placebo and fentanyl will be administered during Period 1 to permit dose escalation to full respiratory effects of fentanyl before assessing the interaction with buprenorphine during the second study period. The low and high doses of buprenorphine listed below have been shown to achieve 50% and > 80% receptor occupancy measured by positron emission tomography with $[^{11}C]$carfentanil radioligand [14, 15]. Buprenorphine doses can be adjusted as needed up to a maximum infusion rate of 0.75 mg/70 kg/h based on experimental observations in Part A.

Buprenorphine:

Low Dose: target concentration 1.0 ng/mL

- 0.25 mg/70 kg bolus
- 0.1 mg/70 kg/h for 360 minutes

High Dose: target concentration 5.0 ng/mL

- 1.25 mg/70 kg bolus
- 0.5 mg/70 kg/h for 360 minutes

Because chronic opioid administration via prescription opioids can elicit marked tolerance to opioid effects, fentanyl bolus dose escalation will be performed on an individual basis. If the initial fentanyl boluses have no visible impact on ventilation, then the escalation will be pushed beyond the doses listed below. The maximum proposed dose is 2.0 mg/70 kg depending on observations during fentanyl dose escalation. If minute ventilation does not fall below 2/3 of baseline at the maximum dose of fentanyl, then this patient will not be continued into the second period and will be replaced.

Fentanyl:

- Bolus 1: 0.25 mg/70 kg
- Bolus 2: 0.35 mg/70 kg (hold if apnoea observed in earlier steps)
- Bolus 3: 0.5 mg/70 kg (hold if apnoea observed in earlier steps)
- Bolus 4: 0.7 mg/70 kg (hold if apnoea observed in earlier steps)

Subjects:

Part A: Healthy subjects

It is anticipated that approximately 18 healthy subjects will be randomised into this part of the study. A minimum of 5 subjects of each sex will be included in the population in order to address any potential differences between sexes. All subjects will be administered buprenorphine and placebo during different periods as part of the cross-over design in Periods 1 and 2; some subjects will also be included in Period 3 and receive buprenorphine. Drop-out subjects may be replaced based on an assessment by the Investigator and sponsor and depending on the proportion of the study already performed by the subject.

Because the dose-response for the buprenorphine-fentanyl interaction has not been previously explored, it may be necessary to include up to 6 additional subjects to fully characterise the range of buprenorphine doses and/or the buprenorphine-fentanyl drug-drug interaction.

Part B: Opioid-tolerant patients

It is anticipated that up to 8 OT patients will be enrolled into the study. A minimum of 3 patients of each sex will be included in the population in order to address any potential differences due to sex. All enrolled patients will be administered placebo and fentanyl in Period 1, and buprenorphine and fentanyl in Period 2. Drop-out patients may be replaced based on an assessment by the Investigator and sponsor and depending on the proportion of the study already performed by the patient.

If healthy subjects cannot tolerate the dose levels of buprenorphine required to sufficiently bind receptors and antagonise fentanyl effects, efforts will be made to recruit up to 24 OT patients to understand the buprenorphine-fentanyl interaction at higher doses of buprenorphine (which are expected to be tolerated by OT patients).

Inclusion Criteria:

Part A: Healthy subjects

1. Signed the informed consent form (ICF) and able to comply with the study requirements and restrictions listed therein;
2. Male and female subjects, age 18 to 45 years, inclusive;
3. Women of childbearing potential (defined as all women who are not surgically sterile or postmenopausal for at least 1 year prior to informed consent) must have a negative serum pregnancy test prior to enrolment and must agree to use a medically acceptable means of contraception from screening through at least 3 months after the last dose of study drug;
4. Body Mass Index (BMI) 18 to 30 kg/m^2, inclusive;
5. Healthy as defined by the Investigator, based on a medical evaluation that includes the subject's medical and surgical history, physical examination, vital signs, 12-lead electrocardiogram (ECG), haematology, blood chemistry, and urinalysis;
6. No history of substance use disorder;
7. No current use of any central nervous system (CNS) depressants prescribed or otherwise.

Part B: Opioid-tolerant patients

1. Signed the ICF and able to comply with the requirements and restrictions listed therein;
2. Male and female, age 18 to 55 years, inclusive;
3. Women of childbearing potential (defined as all women who are not surgically sterile or postmenopausal for at least 1 year prior to informed consent) must have a negative serum pregnancy test prior to enrolment and must agree to use a medically acceptable means of contraception from screening through at least 3 months after the last dose of study drug.
4. BMI 18 to 32 kg/m^2, inclusive;

5. Opioid-tolerant patients administered opioids at daily doses ≥ 90 mg oral morphine equivalents (See Appendix A);
6. Stable as defined by the Investigator, based on a medical evaluation that includes the patient's medical and surgical history, physical examination, vital signs, 12-lead ECG, haematology, blood chemistry, and urinalysis;
7. No current use of any CNS depressants, besides opioids, prescribed or otherwise for 5 half-lives of the product before first study drug administration unless assessed as safe by the principal investigator.

Exclusion Criteria:

Part A: Healthy subjects

1. History of risk factors of Torsades de Pointes (e.g. heart failure, hypokalaemia, family history of Long QT Syndrome) or an ECG demonstrating a Fridericia's corrected QT interval (QTcF) > 450 msec in males and QTcF > 470 msec in females at screening;
2. Currently meet the criteria for diagnosis of substance use disorder according to the Diagnostic and Statistical Manual of Mental Disorders (DSM)-5 criteria on any substance;
3. Any other active medical condition, organ disease or concurrent medication or treatment that may either compromise subject safety or interfere with study endpoints (including sleep apnoea, other significant respiratory illness, history or risk of difficult intubation, limited cervical spine mobility or limited oral excursion);
4. Current smokers and those who have smoked within the last 6 months;
5. Current users of e-cigarettes and nicotine replacement products and those who have used these products within the last 6 months;
6. Consume, on average, > 20 units/week of alcohol in men and > 13 units/week of alcohol in women (1 unit = 1 glass (250 mL) beer, 125 mL glass of wine or 25 mL of 40% spirit);
7. Previous treatment with any prescribed medications (including all type of vaccines) or over-the-counter (OTC) medications (including homeopathic preparations, vitamins, and minerals) within 14 days or 5 half-lives (whichever is longer) prior to first study treatment administration;
8. Previous or current treatment with opioid agonist, partial agonist, or antagonist treatment within 30 days prior to the first study drug administration;
9. Require ongoing prescription or OTC medications that are clinically relevant CYP P450 3A4 or CYP P450 2C8 inducers or inhibitors (e.g., rifampicin, azole antifungals [e.g., ketoconazole], macrolide antibiotics [e.g., erythromycin]);
10. Significant traumatic injury, major surgery, or open biopsy within the prior 4 weeks of informed consent;
11. History of suicidal ideation within 30 days prior to informed consent or history of a suicide attempt in the 6 months prior to informed consent;
12. Measured systolic blood pressure greater than 160 or less than 95 mmHg or diastolic pressure greater than 95 mmHg prior to Day 1;
13. History or presence of allergic response to buprenorphine or fentanyl;
14. Subjects who have demonstrated allergic reactions (e.g., food, drug, atopic reactions or asthmatic episodes) which, in the opinion of the Investigator and sponsor, interfere with their ability to participate in the trial;
15. Estimated glomerular filtration rate <60 mL/min as estimated by the Chronic Kidney Disease Epidemiology Collaboration (CKD-EPI) equation;
16. Anaemia at screening or donation of > 250 mL of blood or plasma within the last 3 months;
17. Positive serology tests for human immunodeficiency virus (HIV), hepatitis B (HBsAg), or hepatitis C (HepC);
18. Aspartate transaminase (AST) or alanine transaminase (ALT) levels >1.5 times the upper limit of normal at screening;

19. Treatment with another investigational drug within 3 months prior to dosing or having participated in more than 4 investigational drug studies within 1 year prior to screening;
20. Site staff or subjects affiliated with, or a family member of, site staff directly involved in the study.

Part B: Opioid-tolerant patients

1. Clinically significant risk factors of Torsades de Pointes (e.g., heart failure, hypokalaemia, family history of Long QT Syndrome) or an ECG demonstrating a Fridericia's corrected QT interval (QTcF) > 450 msec in males and QTcF > 470 msec in females at screening;
2. Currently meet the criteria for diagnosis of moderate or severe substance use disorder according to the DSM-5 criteria on any substances other than opioids, caffeine, or nicotine;
3. Any active medical condition, organ disease or concurrent medication or treatment that may either compromise subject safety or interfere with study endpoints (including sleep apnoea, other significant respiratory illness, history or risk of difficult intubation, limited cervical spine mobility or limited oral excursion);
4. Not able to abstain from smoking cigarettes during each dose administration day;
5. Consume, on average, >27 units/week of alcohol in men and >20 units/week of alcohol in women (1 unit = 1 glass (250 mL) beer, 125 mL glass of wine or 25 mL of 40% spirit);
6. Use of buprenorphine within 10 days of the first study drug administration;
7. Require ongoing prescription or OTC medications that are clinically relevant CYP P450 3A4 or CYP P450 2C8 inducers or inhibitors (e.g., rifampicin, azole antifungals [e.g., ketoconazole], macrolide antibiotics [e.g., erythromycin]);
8. Significant traumatic injury, major surgery, or open biopsy within the prior 4 weeks of informed consent;
9. History of suicidal ideation within 30 days prior to informed consent or history of a suicide attempt in the 6 months prior to informed consent;
10. Measured systolic blood pressure greater than 160 or less than 95 mmHg or diastolic pressure greater than 95 mmHg prior to Day 1;
11. History or presence of allergic response to buprenorphine or fentanyl;
12. Opioid-tolerant patients who have demonstrated allergic reactions (e.g., food, drug, atopic reactions or asthmatic episodes) which, in the opinion of the Investigator and sponsor, interfere with their ability to participate in the trial.
13. Estimated glomerular filtration rate <60 mL/min as estimated by the CKD-EPI equation;
14. Anaemia at screening or donation of > 250 mL of blood or plasma within the last 3 months;
15. Positive serology tests for HIV, acute hepatitis B, or acute hepatitis C (OT patients with asymptomatic hepatitis B or C infection may be enrolled);
16. AST or ALT levels >3.0 times the upper limit of normal at screening;
17. Treatment with another investigational drug within 3 months prior to dosing or having participated in more than 4 investigational drug studies within 1 year prior to screening;
18. Site staff or subjects affiliated with, or a family member of, site staff directly involved in the study.

Primary Endpoints:
Minute ventilation (L/min), respiratory rate (/min), oxygen saturation (SpO2), tidal volume (L), end-tidal P_{CO2} (kPa; PE^{i}_{CO2}) and end-tidal P_{O2} (kPa; PE^{i}_{O2}) are measured for each breath during the baseline period and during infusion of study drugs.

- Peak ventilatory depression (change in minute ventilation) will be calculated based on a 1-minute average of the ventilation data of each individual subject/patient. For buprenorphine or placebo, absolute changes and percentage changes are calculated from the baseline value. For fentanyl, absolute changes and percentage changes for each bolus are calculated from the baseline value and from the pre-fentanyl baseline value immediately fentanyl bolus.

Secondary Endpoints
For Part A (Healthy subjects):

- Number (percentage) of subjects who experience apnoea for each fentanyl dose during the placebo treatment vs. the buprenorphine treatment.
- Number (percentage) of subjects who require stimulation for breathing for each fentanyl dose during the placebo treatment vs. the buprenorphine treatment.

For Part B (Opioid-tolerant patients):

- Whether the subject experiences apnoea during buprenorphine treatment at the fentanyl dose, at which the subject had apnoea during the placebo treatment.
- Fentanyl dose corresponding to the occurrence of apnoea during placebo and buprenorphine infusion periods (if applicable),

Exploratory Endpoints (Safety and tolerability)
- To assess safety as determined by reporting of treatment-emergent (serious) adverse events ((S)AEs), concomitant medications, laboratory test results, vital signs, ECG parameters, physical examination findings, and Columbia-Suicide Severity Rating Scale (C-SSRS) responses;

Exploratory Endpoints (Pharmacokinetic)
The following endpoints will be determined for buprenorphine following each treatment. They will be derived by non-compartmental analysis of the plasma concentration-time data:

- The maximum plasma concentration at the end of the bolus (C_{max});
- The area under the plasma concentration-time curve from zero to t of the last measured concentration above the limit of quantification ($AUC_{0\text{-last}}$);
- The average concentration during the fentanyl dose escalation C_{avg} [2-6h], which is calculated as AUC[2-6h]/4h;

The following endpoints will be determined for fentanyl following each bolus (as appropriate). They will be derived by non-compartmental analysis of the plasma concentration-time data:

- The area under the plasma concentration-time curve for each dosing interval ($AUC_{0\text{-tau}}$);
- The maximum plasma concentration for each dosing interval (C_{max});
- The time to reach maximum plasma concentration for each dosing interval (t_{max});
- Other parameters, including apparent volume of distribution (Vz/F), apparent clearance (CL/F), and other parameters as appropriate, as well as dose adjusted parameters, may be determined.

Exploratory endpoints (Pharmacodynamic)

Minute ventilation (L/min), respiratory rate (/min), tidal volume (L), oxygen saturation (SpO_2), end-tidal P_{CO2} (kPa; PE^{i}_{CO2}) and end-tidal P_{O2} (kPa; PE^{i}_{O2}) are measured for each breath during the baseline period and during infusion of study drugs. The following parameters will be calculated:

- Peak changes in other ventilation parameters will be calculated for buprenorphine or placebo, absolute changes and percentage changes are calculated from the baseline value. For fentanyl, absolute changes and percentage changes for each bolus are calculated from the baseline value and from the pre-fentanyl baseline value immediately before the first fentanyl bolus.
- Area under the curve in ventilation parameters will be calculated based on a 1-minute average of the ventilation data of each individual subject/patient. For buprenorphine or placebo, changes are calculated from the baseline value. For fentanyl, changes are calculated for each bolus are calculated from the baseline value and from the pre-fentanyl baseline value immediately before the first fentanyl bolus.
- When possible, time to peak effect (min) and time to end of effect (i.e., return to baseline in minutes) will be calculated for each for the initial buprenorphine/placebo period and each fentanyl bolus.
- EC_{50} and E_{max} for buprenorphine and fentanyl effects on minute ventilation as determined by PK/PD models.
- Sedation Visual Analogue Scale (VAS) administered before the first fentanyl bolus and at the conclusion of each bolus period

Sample Size Justification:

The study is designed to evaluate the exposure-response relationship of fentanyl on (suppression of) ventilation when placebo is administered and in the presence of various concentrations of buprenorphine. Dose-cohort sizes of 3 to 6 subjects with 4 doses of fentanyl and at 2 to 3 doses of buprenorphine have been shown to provide adequate ranges of drug concentrations to construct PK/PD models of drug exposure and response [16, 20 and 21].

Statistical Methodology:

Statistical analysis will consist of summary statistics and graphical representation, which will be detailed in a Statistical Analysis Plan (SAP). Further exploratory statistical analysis may be attempted with statistical testing; e.g., Fisher's exact test on number of subjects with apnoea.

Pharmacokinetic and PK/PD modelling will be described in a detailed Pharmacometric Analysis Plan (PAP).

TABLE 1: SCHEDULE OF ASSESSMENTS – PART A OVERVIEW OF STUDY

Visit	Screening	Period 1		Period 2		Period 3[l]		EOS/ET[m]
**Location**	**CHDR**	**CHDR**	**LUMC**	**CHDR**	**LUMC**	**CHDR**	**LUMC**	**CHDR**
**Day**	**Day -31 to -2**	**Day -1 [a]**	**Day 1[b]**	**Day 10 [a]**	**Day 11 [b]**	**Day 20 [a]**	**Day 21 [b]**	**Day 31**
**Visit Window**				**+7 Days**	**+7 Days**	**+7 Days**	**+7 Days**	**+7 days**
Informed Consent	X							
Demography	X							
Medical History [c]	X							
Height	X							
Adverse Events	X	X	X	X	X	X	X	X
Concomitant Medications	X	X	X	X	X	X	X	X
12-lead Electrocardiogram	X	X	X	X	X	X	X	X
Physical Examination [d]	X	X		X		X		X
Weight	X	X	X	X	X	X	X	X
BMI	X							X
Vital Signs	X	X	X	X	X	X	X	X
Screening Laboratories [e]	X							
Safety Chemistries [f]	X	X		X		X		X
Haematology [g]	X	X		X		X		X
Serum Pregnancy	X							
Urine Pregnancy [h]		X		X		X		X
Urine Drug Screen [h]	X	X		X		X		
Alcohol Breath Test	X	X		X		X		
Urinalysis	X							
C-SSRS	X [k]	X		X		X		
Inclusion/Exclusion Criteria Assessment	X	X						

Visit	Screening	Period 1		Period 2		Period 3[l]		EOS/ET[m]
**Location**	**CHDR**	**CHDR**	**LUMC**	**CHDR**	**LUMC**	**CHDR**	**LUMC**	**CHDR**
**Day**	**Day -31 to -2**	**Day -1 [a]**	**Day 1[b]**	**Day 10 [a]**	**Day 11 [b]**	**Day 20 [a]**	**Day 21 [b]**	**Day 31**
**Visit Window**				**+7 Days**	**+7 Days**	**+7 Days**	**+7 Days**	**+7 days**
Clinical Unit Admission		X		X		X		
Anaesthesia Suite Admission [i]			X		X		X	
Ventilation Monitoring [i]			X		X		X	
Ondansetron dosing [j]			X		X		X	
Investigation Drug Dosing			X		X		X	
Pharmacokinetics			X		X		X	
Sedation VAS			X		X		X	

EOS=end of study; ET=early termination; BMI = Body Mass Index; CHDR=Centre for Human Drug Research; C-SSRS= Columbia Suicide Severity Rating Scale; LUMC=Leiden University Medical Centre; PACU =Post-Anaesthesia Care Unit

a. Healthy subjects (Part A) are admitted to CHDR 1 day before transfer to LUMC for infusion studies and OT patients for both Periods 1 and 2. Pre-randomisation procedures will be done on Day -1.

b. After scheduled assessments are completed, subjects will be transferred to the PACU for overnight monitoring.

c. Comprehensive medical and psychiatric history including use of nicotine and tobacco, drugs of abuse and alcohol.

d. Complete physical examinations at Screening and EOS/ET; brief at interim time points.

e. Blood specimens will be collected following at least a 4-hour fast. Screening laboratories to include albumin, haemoglobin A1C, calcium, cholesterol, triglycerides, TSH, protein, amylase, lipase, PT/INR, urine analysis (Leukocytes, blood, nitrite, protein, urobilinogen, bilirubin, pH, specific gravity, ketones, glucose), HIV1 and HIV2 antigen and antibodies, hepatitis B surface antigen, hepatitis B antibodies and hepatitis C antibodies.

f. Blood specimens will be collected following at least a 4-hour fast. Safety chemistries to include sodium, potassium, chloride, CO_2, glucose, blood urea nitrogen, creatinine, alanine aminotransferase, aspartate aminotransferase, alkaline phosphatase and bilirubin

g. Haematology to include haemoglobin (including MCV, MCH, MCHC, haematocrit, red cell count, total white cell count and platelet count). Differential blood count, including: basophils, eosinophils, neutrophils, lymphocytes and monocytes.

h. Urine pregnancy tests and drug screens to be performed in the clinic via dipstick.

i. Subjects are admitted to the anaesthesia suite for placement of facemask, continuous monitoring of ventilation and haemodynamics and intravenous infusion of opioid drugs.

j. Once ventilation is stable for 30-45 mins, healthy subjects will receive 4 mg ondansetron before infusion of buprenorphine or placebo is started. Pre-dose vitals should be performed before ondansetron dosing.

k. The baseline/screening version of the C-SSRS will be performed at the screening visit. The 'Since last Visit' version of the C-SSRS will be used for all other visits.

l. Period 3 is optional, and not all subjects are required to participate; the sponsor and Investigator collectively determine if Period 3 is required for each subject. Subjects will perform EOS/ET 10-17 days after their last study period.

TABLE 2: SCHEDULE OF ASSESSMENTS – PART B OVERVIEW OF STUDY

Visit	Screening	Period 1			Period 2		EOS/ET
Location	CHDR	CHDR	CHDR	LUMC	CHDR	LUMC	CHDR
Day	Day -31 to -3	Day -2 [a]	Day -1	Day 1 [b]	<u>Day 2</u>	Day 3	Day 13
Visit Window							+7 days
Informed Consent	X						
Demography	X						
Medical History [c]	X						
Height	X						
Adverse Events	X	X	X	X	X	X	X
Concomitant Medications	X	X	X	X	X	X	X
12-lead Electrocardiogram	X	X		X		X	X
Physical Examination [d]	X	X					X
Weight	X		X		X		X
BMI	X						X
Vital Signs	X	X		X		X	X
Screening Laboratories [e]	X						
Safety Chemistries [f]	X	X			X		X
Haematology [g]	X	X			X		X
Serum Pregnancy	X						
Urine Pregnancy [h]		X					X
Urine Drug Screen [h]	X	X					
Alcohol Breath Test	X	X					
Urinalysis	X						
C-SSRS	X[l]	X					
Inclusion//Exclusion Criteria Assessment	X	X					
Clinical Unit Admission		X					
Management of Opioid Dosing (OT patients) [i]		X	X		X		
Anaesthesia Suite Admission [j]				X		X	
Fentanyl and Buprenorphine Infusion				X		X	
Ventilation Monitoring [j]				X		X	
Pharmacokinetics [k]				X		X	
Sedation VAS				X		X	

EOS=end of study; ET=early termination; BMI = Body Mass Index; CHDR=Centre for Human Drug Research; C-SSRS= Columbia Suicide Severity Rating Scale; LUMC=Leiden University Medical Centre; PACU =Post-Anaesthesia Care Unit; OT = opioid tolerant

a. Opioid-tolerant patients (Part B) are admitted to CHDR at least 48 hours before transfer to LUMC in Period 1.

b. After scheduled assessments are completed, patients will be transferred to the PACU for overnight monitoring. Patients will return to CHDR until doing Period 2.

c. Comprehensive medical and psychiatric history including use of nicotine and tobacco, drugs of abuse (lifetime and past 30 days) and alcohol.

d. Complete physical examinations at Screening and EOS/ET; brief at interim time points.

e. Blood specimens will be collected following at least a 4-hour fast. Screening laboratories to include albumin, haemoglobin A1C, calcium, cholesterol, triglycerides, TSH, protein, amylase, lipase, PT/INR, urine analysis (Leukocytes, blood, nitrite, protein, urobilinogen, bilirubin, pH, specific gravity, ketones, glucose), HIV1 and HIV2 antigen and antibodies, hepatitis B surface antigen, hepatitis B antibodies and hepatitis C antibodies.

f. Blood specimens will be collected following at least a 4-hour fast. Safety chemistries to include sodium, potassium, chloride, CO_2, glucose, blood urea nitrogen, creatinine, alanine aminotransferase, aspartate aminotransferase, alkaline phosphatase and bilirubin.

g. Haematology to include haemoglobin (including MCV, MCH, MCHC, haematocrit, red cell count, total white cell count and platelet count). Differential blood count, including: basophils, eosinophils, neutrophils, lymphocytes and monocytes.
h. Urine pregnancy tests and drug screens to be performed in the clinic via dipstick.
i. Opioid-tolerant patients receive stable opioid doses of oral oxycodone for at least 48 hours before dosing and through end of Period 2. Breakthrough pain can be managed by doses of ibuprofen and paracetamol.
j. Subjects are admitted to the anaesthesia suite for placement of facemask, continuous monitoring of ventilation and haemodynamics and intravenous infusion of opioid drugs.
k. In Part B, blood samples will be collected from OT patients for fentanyl only in Period 1 and for buprenorphine and fentanyl in Period 2.
l. The 'Baseline/screening' version of the C-SSRS will be performed at the Screening visit. The 'Since last Visit' version of the C-SSRS will be used for all other visits.

TABLE 3: SCHEDULE OF ASSESSMENTS – PART A PERIOD ASSESSMENTS AT LUMC

Time [i]	Pre-	0	5	10	15	20	30	60	90	120	122	125	130	135	140	150	180	182	185	190	195	200	210	240	242	245	250	255	260	270	300	302	305	310	315	320	330	360	375	420	480	540	900[k]
LUMC Admission [a]	X																																										
Ondansetron Dosing [b]	X																																										
Ventilation Monitoring [c]	X	X	X	X	X	X	X	X	X	X	X	X	X	X	X	X	X	X	X	X	X	X	X	X	X	X	X	X	X	X	X	X	X	X	X	X	X	X					
Adverse Events [d]	X	X	X	X	X	X	X	X	X	X	X	X	X	X	X	X	X	X	X	X	X	X	X	X	X	X	X	X	X	X	X	X	X	X	X	X	X	X	X	X	X	X	X
Concomitant Medications [d]	X	X	X	X	X	X	X	X	X	X	X	X	X	X	X	X	X	X	X	X	X	X	X	X	X	X	X	X	X	X	X	X	X	X	X	X	X	X	X	X	X	X	X
Continuous Monitoring [f]	X	X	X	X	X	X	X	X	X	X	X	X	X	X	X	X	X	X	X	X	X	X	X	X	X	X	X	X	X	X	X	X	X	X	X	X	X	X	X	X	X	X	X
Weight	X																																										
Vital Signs [e]	X				X		X	X		X							X							X							X							X		X	X		X
12-lead Electrocardiogram	X				X		X	X		X							X							X							X							X		X	X		X
Bup 6mL PK Sample	X		X	X	X	X	X	X	X	X							X							X							X							X	X	X	X	X	
Fentanyl 2mL PK Sample [j]										X	X	X	X	X	X	X	X	X	X	X	X	X	X	X	X	X	X	X	X	X	X	X	X	X	X	X	X	X	X	X	X	X	
Buprenorphine or Placebo Infusion [g]		X	X	X	X	X	X	X	X	X	X	X	X	X	X	X	X	X	X	X	X	X	X	X	X	X	X	X	X	X	X	X	X	X	X	X	X	X					
Fentanyl Dose Escalation [j]										X							X							X							X												
Sedation VAS [h]	X									X							X							X							X							X					
Transfer to PACU																																											X

Bup=buprenorphine; LUMC=Leiden University Medical Centre; PACU= Post-Anaesthesia Care Unit; PK=pharmacokinetics

a. Subjects will be escorted from CHDR and will be admitted to LUMC on morning of Day 1.

b. After baseline ventilation stabilises (30 to 45 minutes), subjects (all healthy subjects) will receive ondansetron 4 mg intravenously (IV), Doses must be at least 15 minutes pre-dose buprenorphine/placebo, and after pre-dose vital signs. Subjects can receive ondansetron 4 mg doses for nausea up to a maximum of 8 mg during the study period.

c. Placement of ventilation mask and continuous monitoring of ventilatory parameters to occur. Minute ventilation (L/min), respiratory rate (/min), oxygen saturation (SpO_2), tidal volume (L), end-tidal P_{CO2} (kPa; PE'_{CO2}) and end-tidal P_{O2} (kPa; PE'_{O2}) will be stored on a breath-to-breath basis and on an average ventilation of 1 minute. If the study subject experiences discomfort, the ventilation mask may be removed at the discretion of the investigator between 30 minutes and 105 minutes.

d. Adverse Events and Concomitant Medications will be recorded throughout stay in LUMC. Significant procedure-related events will be recorded as procedure-related AEs.
e. Vital signs include blood pressure (systolic, diastolic pressure), heart rate, respiratory rate, temperature (tympanic) and saturation. Pre-dose vital signs to be completed pre-dose ondansetron.
f. Continuous Vitals and ECG monitoring will occur, clinical significant events will be recorded as AEs.
g. Buprenorphine or placebo infusion will start at 0 minutes with the priming dose, decrease at 15 minutes with continuous dose and stop at 360 minutes.
h. Subjects will answer a question regarding sedation before the start of buprenorphine/placebo administration, before the first fentanyl bolus and 1 hour after each bolus (120, 180, 240, 300 and 360 minutes).
i. Deviations of actual time points from the expected time points will be within 10%, calculated from the zero point (time of start drug administration) or the last relevant activity. Deviations of more than 10% will be explained in a note. Pre-dose assessments are given in indicative expected times.
j. Fentanyl dosing and fentanyl PK sample collection will occur in Period 1 and 2; these will not occur in Period 3. If a fentanyl bolus is not administered, subsequent fentanyl PK samples will not be collected.
k. 900-minute assessments may be performed at any time once the subject is stabilised and before transferring to PACU.

TABLE 4: SCHEDULE OF ASSESSMENTS – PART B PERIOD ASSESSMENTS AT LUMC

Time [j]	Pre-dose	0	5	10	15	20	30	60	90	120	122	125	130	135	140	150	180	182	185	190	195	200	210	240	242	245	250	255	260	270	300	302	305	310	315	320	330	360	375	420	480	540	900[k]
LUMC Admission [a]	X																																										
Ondansetron Dosing [b]	X																																										
Ventilation Monitoring [c]	X	X	X	X	X	X	X	X	X	X	X	X	X	X	X	X	X	X	X	X	X	X	X	X	X	X	X	X	X	X	X	X	X	X	X	X	X	X					
Adverse Events [d]	X	X	X	X	X	X	X	X	X	X	X	X	X	X	X	X	X	X	X	X	X	X	X	X	X	X	X	X	X	X	X	X	X	X	X	X	X	X	X	X	X	X	X
Concomitant Medications [d]	X	X	X	X	X	X	X	X	X	X	X	X	X	X	X	X	X	X	X	X	X	X	X	X	X	X	X	X	X	X	X	X	X	X	X	X	X	X	X	X	X	X	X
Continuous Monitoring [f]	X	X	X	X	X	X	X	X	X	X	X	X	X	X	X	X	X	X	X	X	X	X	X	X	X	X	X	X	X	X	X	X	X	X	X	X	X	X	X	X	X	X	X
Weight																																											
Vital Signs [e]	X				X		X	X		X							X							X							X							X		X	X		X
12-lead Electrocardiogram	X				X		X	X		X							X							X							X							X		X	X		X
Bup 6mL PK Arterial Sample [i]	X		X	X	X	X	X	X	X	X							X							X							X							X	X	X	X	X	
Bup 6mL PK Venous Sample [i]										X							X							X							X							X					
Fentanyl 2mL PK Sample										X	X	X	X	X	X	X	X	X	X	X	X	X	X	X	X	X	X	X	X	X	X	X	X	X	X	X	X	X	X	X	X	X	
Buprenorphine Infusion [g]		X	X	X	X	X	X	X	X	X	X	X	X	X	X	X	X	X	X	X	X	X	X	X	X	X	X	X	X	X	X	X	X	X	X	X	X	X					
Fentanyl Dose Escalation										X							X							X							X												
Sedation VAS [h]	X									X							X							X							X								X				
Transfer to PACU																																											X

Bup=buprenorphine; LUMC=Leiden University Medical Centre; PACU= Post-Anaesthesia Care Unit; OT = opioid tolerant; PK=pharmacokinetics

a. Opioid-tolerant patients will be escorted from CHDR and will be admitted to LUMC on morning of Day 1. Between study periods patients will return to CHDR and be transferred to LUMC on Day 3.

b. After baseline ventilation stabilises (30 to 45 minutes), OT patients (only when needed) will receive ondansetron 4 mg intravenously (IV), Doses must be at least 15 minutes pre-dose buprenorphine/placebo, and after pre-dose vital signs. OT patients can receive ondansetron 4 mg doses for nausea up to a maximum of 8 mg during the study period.
c. Placement of ventilation mask and continuous monitoring of ventilatory parameters to occur. Minute ventilation (L/min), respiratory rate (/min), oxygen saturation (SpO2), tidal volume (L), end-tidal P_{CO2} (kPa; PE^{i}_{CO2}) and end-tidal P_{O2} (kPa; PE^{i}_{O2}) will be stored on a breath-to-breath basis and on an average ventilation of one minute. If the study subject experiences discomfort, the ventilation mask may be removed at the discretion of the investigator between 30 minutes and 105 minutes.
d. Adverse Events and Concomitant Medications will be recorded throughout stay in LUMC. Significant procedure-related events will be recorded as procedure-related AEs.
e. Vital signs include blood pressure (systolic, diastolic pressure), heart rate, respiratory rate, temperature (tympanic) and saturation. Pre-dose vital signs to be completed pre-dose ondansetron.
f. Continuous vitals and ECG monitoring will occur, clinical significant events will be recorded as AEs.
g. Placebo will be administered in Period 1; buprenorphine will be administered in Period 2. Buprenorphine infusion will start at 0 minutes with the priming dose, decrease at 15 minutes with continuous dose and stop at 360 minutes.
h. Opioid-tolerant patients will answer a question regarding sedation before the start of buprenorphine/placebo administration, before the first fentanyl bolus and 1 hour after each bolus (120, 180, 240, 300 and 360 minutes).
i. Buprenorphine PK samples will be collected in Period 2 only.
j. Deviations of actual time points from the expected time points will be within 10%, calculated from the zero point (time of start drug administration) or the last relevant activity. Deviations of more than 10% will be explained in a note. Pre-dose assessments are given in indicative expected times.
k. 900-minute assessments may be performed at any time once subject is stabilised and before transferring to PACU.

1 BACKGROUND AND RATIONALE

1.1 Context

Prescription opioid medications, such as oxycodone, are amongst the most commonly used analgesics. Physicians worldwide prescribe opioids for patients in many different clinical settings, including patients with post-operative pain or pain due to cancer. Unfortunately, opioid administration also results in many unwanted pharmacological effects, including opioid-induced respiratory depression, which is potentially fatal. Opioids activate μ-opioid receptors at specific sites in the central nervous system (CNS) including the pre-Bötzinger complex, a respiratory rhythm generating area in the pons, causing decreased ventilatory response [17]. The result can be hypercapnia, hypoxia and irregular breathing, and at high dosages, a complete cessation of rhythmic respiratory activity can occur [17-19].

In patients with opioid use disorder (OUD), respiratory depression is a major cause of death. According to the 2016 National Survey on Drug Use and Health, there were 20.1 million people aged 12 or older in the Unites States of America (USA) who suffered from substance use disorder [1]. In 2015 alone, the harm caused by drug use translated into an estimated 28 million years of healthy life lost worldwide due to premature death and disability [4]. In the USA, the misuse of opioids accounted for approximately 25% of the number of drug-related deaths worldwide, increasing from 16,849 to 52,404 annually during the 1999-2015 period [5, 6]. In Europe, the use of prescription opioids has also increased, but this change has not been associated with a marked increase in overdose deaths [7]. Still, some regions have reported dramatic increases in deaths due to overdose, and the common thread among these reports has been widespread access to highly potent fentanyl and carfentanil [8-11]. The risk of overdose deaths is particularly high for problem drug users after periods of relative abstinence, most notably soon after prison release, but also after hospital discharge. The most probable reason is loss of tolerance to the effects of opioids [8], but the changes in tolerance have not been well-characterised.

Many patients are not aware that addiction is a disease that can be medically treated [2]. The goals of medication-assisted treatment (MAT) are to reduce substance use and risk of relapse or overdose, to reduce harm from sequelae of substance abuse and to help patients return to a healthy, functional life [3]. Buprenorphine, a partial agonist at the μ-opioid receptor (MOR) is used for the MAT of OUD. Buprenorphine has high affinity for the MOR and therapeutic plasma concentrations achieve ≥70% receptor occupancy. As a partial agonist, buprenorphine has a ceiling effect on respiratory depression such that it does not cause apnoea when administered alone and minute ventilation is not suppressed beyond 50 to 60% [12]. It is hypothesised that buprenorphine will competitively inhibit the effects of potent, short-acting MOR agonists like fentanyl and carfentanil that can result in apnoea and death. The objective of this trial is to determine if MOR blockade with buprenorphine can shift the respiratory depression response to intravenous (IV) fentanyl injection to the right, thereby reducing the potency of fentanyl in causing respiratory depression - the usual fatal precipitant associated with IV fentanyl/heroin overdose.

1.2 Clinical pharmacology information

Buprenorphine:

Buprenorphine, an analgesic opioid, is a partial agonist at the μ-opioid receptor and an antagonist at the κ (kappa) receptor. The medication is readily bioavailable by IV or intramuscular (IM) routes. Buprenorphine is oxidatively metabolised by 14-N-dealkylation to N-dealkyl-buprenorphine (also known as norbuprenorphine) via cytochrome P450 CYP3A4 and by glucuronidation of the parent molecule and the dealkylated metabolite. Norbuprenorphine is a μ agonist with weak intrinsic activity. Elimination of buprenorphine is bi- or tri-exponential, with a long terminal elimination phase of 20 to 25 hours, due in part to reabsorption of buprenorphine after intestinal hydrolysis of the conjugated derivative, and in part to the highly lipophilic nature of the molecule. Buprenorphine is essentially eliminated in the faeces by biliary excretion of the glucuronidated metabolites (80 %), the rest being eliminated in the urine.

Fentanyl:

Fentanyl is a potent opioid analgesic with a high affinity for the μ-opioid receptor. It is used as an analgesic supplement to general anaesthesia or as the sole anaesthetic. The onset of action is rapid. However, the

maximum analgesic and respiratory depressant effect may not be noted for several minutes. The usual duration of action of the analgesic effect is approximately 30 minutes after a single IV dose of up to 100 µg. The potency of analgesia is dose-related and can be adjusted based on the pain level of the surgical procedure. Fentanyl is a lipid-soluble drug and its pharmacokinetics can be described in terms of a 3-compartment model. Following IV injection, there is a short distribution phase during which high concentrations of fentanyl are achieved quickly in well-perfused tissues such as the lungs, kidneys and brain. The drug is redistributed to other tissues; it accumulates more slowly in skeletal muscle and yet more slowly in fat, from which it is gradually released into the blood. Up to 80% of fentanyl is bound to plasma proteins. Fentanyl is primarily metabolised in the liver, probably by N-dealkylation, and it is excreted mainly in the urine with less than 10% representing the unchanged drug. The terminal half-life of fentanyl is 3.7 hours.

1.3 Study rationale

1.3.1 Benefit and risk assessment

Buprenorphine:
Adverse drug reactions commonly reported are sedation, dizziness, sleep, miosis, hypoventilation, nausea, vomiting, hyperhidrosis and headache. Hallucinations and other psychotomimetic effects can occur although more rarely. Hypotension leading to syncope can occur. Buprenorphine may cause significant respiratory depression when taken in combination with benzodiazepines or other CNS depressants.

Care will be taken when treating patients with impaired respiratory function or patients who are receiving drugs that can cause respiratory depression. Experience has shown that naloxone is beneficial in reversing a reduced respiratory rate. Respiratory stimulants such as doxapram are also effective. The intensity and duration of action is affected in patients with impaired liver failure, which will be assessed during screening.

All healthy subjects will receive ondansetron 4 mg IV prior to buprenorphine infusion. A second dose of 4 mg can be administered as needed for management of nausea and vomiting. Patients administered chronic opioids can receive no more than two 4-mg IV doses of ondansetron as needed for management of nausea and vomiting.

Fentanyl:
The most commonly reported adverse drug reactions are nausea, vomiting, muscle rigidity, hypotension or hypertension, bradycardia and sedation. Other adverse reactions that have been reported are dizziness, blurred vision, nausea, vomiting, hyperhidrosis, pruritus, urticaria, laryngospasm and anaphylaxis.

Fentanyl will be given only in an environment where the airway can be controlled and by personnel who will monitor the airway. Respiratory depression is dose-related and can be reversed by an antagonist such as naloxone. Multiple doses of naloxone may be necessary because the respiratory depression will last longer than the duration of action of the opioid antagonist. Subjects will remain under appropriate surveillance. Resuscitation equipment and opioid antagonists will be readily available. Adequate spontaneous breathing must be established and maintained before discharge from the Post-Anaesthesia Care Unit (PACU).

1.3.2 Medical and regulatory background

Buprenorphine for IV or intramuscular (IM) injection is indicated for the treatment of postoperative pain in non-ambulatory patients. Additional dose formulations include transdermal, sublingual/transmucosal and oral dosage forms. Transdermal buprenorphine is indicated for management of moderate to severe chronic pain in patients requiring a continuous, around-the-clock opioid analgesic for an extended time. Additional dosage forms are primarily utilised for maintenance treatment of opioid dependence. The recommended analgesic dosage is 0.3 – 0.6 mg buprenorphine administered via IM injection or slow IV injection, repeated if necessary every 6 to 8 hours. A more accurate dose calculation is 4 µg per kg of body weight.

Fentanyl citrate for injection is an opioid agonist indicated for:

- short duration analgesia during the anaesthetic periods, premedication, induction and maintenance, and in the immediate postoperative period (recovery room) as the need arises.
- use as an opioid analgesic supplement in general or regional anaesthesia.
- administration with a neuroleptic as an anaesthetic premedication, for the induction of anaesthesia and as an adjunct in the maintenance of general and regional anaesthesia.
- use as an anaesthetic agent with oxygen in selected high-risk patients, such as those undergoing open heart surgery or certain complicated neurological or orthopaedic procedures.

It should be administered only by persons specifically trained in the use of IV anaesthetics and management of the respiratory effects of potent opioids who have resuscitative and intubation equipment and readily-available oxygen. The suggested starting dose in adults is 50 – 100 µg (0.05 to 0.1 mg). Like buprenorphine, fentanyl is available in a transdermal formulation indicated for extended management of moderate to severe chronic pain.

Opioid medications such as buprenorphine and fentanyl remain a viable option for treatment of patients with any type of pain. However, due to the potentially fatal consequences of opioid-induced respiratory depression, effective treatment with opioids remains challenging and is often limited to partial treatment. Full opioid agonists like fentanyl affect breathing and have onset and offset profiles that are primarily determined by opioid transfer to the receptor site. Complete reversal can be obtained with a single naloxone dose. The objective of this trial is to determine if MOR blockade with buprenorphine can shift the respiratory depression response to IV fentanyl injection to the right, thereby reducing the potency of fentanyl in causing respiratory depression in patients.

1.3.3 Study population

The study is designed to evaluate the exposure response relationship of fentanyl on (suppression of) ventilation when placebo is administered and in the presence of various concentrations of buprenorphine. Dose-cohort sizes of 3 to 6 subjects with 4 doses of fentanyl and 2 to 3 doses of buprenorphine have been shown to provide adequate ranges of drug concentrations to construct pharmacokinetic-pharmacodynamic (PK/PD) models of drug exposure and response [16, 20, 21].

Part A (Healthy subjects):

Approximately 18 male and female subjects between the ages of 18 and 45 years will be enrolled into the clinical study after having been checked for their eligibility in a screening examination, performed between Day -31 and Day -2 prior to the first study drug administration day. Within the 18 subjects, a minimum of 5 subjects of each sex will be included to address any potential differences due to sex.

Per 3 subjects, the buprenorphine dose will be determined based on safety and pharmacodynamic results, leading to approximately 6 dose cohorts and a minimum of 3 subjects assigned to any buprenorphine dose level. Every effort will be made to represent both sexes in each buprenorphine dose level. Because the dose-response effects of the buprenorphine-fentanyl interaction have not been previously explored, it may be necessary to include up to 6 additional subjects in 2 additional dose cohorts to fully characterise the range of buprenorphine doses and/or the fentanyl drug-drug interaction.

Part B (Opioid-tolerant patients):

Approximately 8 male and female opioid-tolerant (OT) patients between the ages of 18 and 55 years will be enrolled into the clinical study after having been checked for their eligibility in a screening examination, performed between Day -31 and admission prior to the first study drug administration day. A minimum of 3 subjects of each sex should be included in the population in order to address any potential sex differences. If healthy subjects cannot tolerate higher doses of buprenorphine required to bind receptors and antagonise fentanyl effects, efforts will be made to recruit up to 24 OT patients (split between males and females as equally as possible) to understand the buprenorphine-fentanyl interaction at higher doses of buprenorphine.

1.3.4 Study design

This study will be performed in 2 parts (Part A and Part B) at 2 clinical study sites. Dosing day procedures will be performed at Leiden University Medical Centre (LUMC), and all other study procedures will be performed at the Centre for Human Drug Research (CHDR).

Part A is a 3-period study in approximately 18 healthy subjects. The first 2 periods will have a single-blind, placebo-controlled, cross-over design; subjects will be randomised in a 1 to 1 ratio to 2 treatment sequences determined by the order in which they receive buprenorphine or placebo. Period 3 will have an open label design, where the same subjects will receive buprenorphine only. Period 3 is optional, and not all subjects are required to participate; the sponsor and investigator collectively determine if Period 3 is required for each subject. In total, about 6 subjects will participate in a third investigational period.

Part B is an open-label study in OT patients receiving ≥ 90 morphine equivalents per day. This exceeds doses listed in prescribing information stating that patients who are opioid tolerant are those receiving, for 1 week or longer, at least 60 mg oral morphine per day. Opioid-tolerant patients will receive placebo and fentanyl challenges in Period 1, in order to optimise the fentanyl dose escalation before buprenorphine and fentanyl are co-administered in Period 2.

1.3.5 Comparative drug(s) and/or placebo

0.9% normal saline for IV administration will be used as placebo matching the buprenorphine formulation.

1.3.6 Dose rationale, dose determination and stopping criteria

This investigation explores the pharmacodynamic interaction between fentanyl, a full MOR receptor agonist, and buprenorphine, a partial MOR agonist, focusing on respiratory depression. The pharmacokinetics and respiratory effects of each individual medication have been documented in a series of studies conducted in healthy subjects [12, 13, 17, 20, 21 and 26]. Dose-response relationships for analgesia were observed with fentanyl in the range of 0 to 9 µg/kg and for buprenorphine at 0 to 7 µg/kg (fentanyl and buprenorphine were equipotent relative to morphine). Fentanyl caused dose-dependent reduction of minute ventilation with respiratory instability at doses of 3 ug/kg and greater. In one subject, prolonged periods of apnoea were observed at the highest dose tested (0.5 mg in a 70-kg subject) and this person was managed with supplemental oxygen and assisted ventilation. Buprenorphine similarly caused dose-dependent reduction of minute ventilation, but in contrast to fentanyl, a plateau in the respiratory depression (about 50% of baseline) occurred at dosages ≥ 3 µg/kg. Importantly, no respiratory instability, periodic breathing or apnoea occurred, even at the highest dose tested (0.6 mg in a 70-kg subject).

All procedures will be conducted in an anaesthesia suite with access to continuous monitoring of ventilation and arterial blood oxygenation, naloxone for potential reversal of opioid effects, and mechanical ventilatory support. For Part A conducted in healthy subjects without opioid tolerance, buprenorphine dosing is flexible and infusion rates will be selected to target approximately 25 to 50% respiratory depression. Buprenorphine will be administered as a primed-continuous infusion with an initial bolus administered over 15 minutes and infusion continued to complete 360 minutes of administration. The initial infusion rate will be 10-fold the hourly infusion rate to speed attainment of steady-state buprenorphine concentrations at the site of action. The initial bolus could range from 0.0125 to 0.5 mg/70 kg, with the infusion continued at 0.005 to 0.2 mg/70 kg/h (Table 5 and Table 6). The planned infusion rates are consistent with prior studies of buprenorphine administration that evaluated naloxone reversal of buprenorphine effects with continuous infusion of buprenorphine at rates of 0.1 and 0.2 mg/70 kg/h for one hour [21, 22]. The most common expected adverse events (AEs) are likely to be nausea, vomiting, sedation and a decrease in minute ventilation. Gastrointestinal side effects will be managed with prior infusion of 4 mg of ondansetron.

Table 5: Proposed range of buprenorphine doses for healthy subjects and opioid-tolerant patients

Healthy Subjects		Opioid-tolerant Patients	
**Bolus mg/70kg**	**Infusion mg/70kg/hr**	**Bolus mg/70kg**	**Infusion mg/70kg/hr**
0.0125	0.005		
0.025	0.01		
0.05	0.02		
0.125	0.05		
0.25	0.1	0.25	0.1
0.5	0.2	0.5	0.2
		1.25	0.5
		1.875	0.75

Fentanyl will be administered as a bolus over 90 seconds and will be given as a dose escalation. The fentanyl doses will increase from 0.075 to 0.15, 0.25 and 0.35 mg/70 kg. Brief periods of apnoea could be expected starting at 0.25 mg/70 kg (approximately 3.5 µg/kg) [13, 21]. Dose escalations will be limited if a procedure-related AE occurs. A procedure-related AE is defined by loss of respiratory activity for 60 seconds or longer, despite active stimulation of the subject, end-tidal partial pressure of carbon dioxide greater than 67.5 mmHg, O_2 saturation less than 85% for at least 2 minutes, or any other situation or condition that may interfere with the health of the participant. If investigators stimulate the subjects to breathe or give supplemental oxygen as needed to prevent an AE, the subject will not proceed to the next fentanyl dose and the study session will terminate early.

For Part B conducted in OT patients, the buprenorphine dosing will again be flexible. These patients develop opioid tolerance to the desired effects of these drugs [23, 25]. The initial target low and high doses of buprenorphine are expected to achieve plasma drug concentrations of 1 and 5 ng/mL, which result in 50% and >80% receptor occupancy, as measured by positron emission spectrometry with [^{11}C]-carfentanil radioligand [14, 15]. The potential range of buprenorphine doses is shown in Table 5, and doses can be adjusted as needed up to a maximum infusion rate of 0.75 mg/70 kg/h based on experimental observations. Buprenorphine concentrations need to be > 1 ng/mL to limit withdrawal side effects and ≥ 2 ng/mL to limit drug liking and craving in OUD patients undergoing medication-assisted treatment (MAT) [14, 24]. In an opioid-tolerant population, the higher buprenorphine concentrations are well tolerated, and the most frequently reported AEs are likely to be headache, sedation, dizziness, nausea and vomiting [24].

The dosages and plasma concentrations of fentanyl that lead to respiratory depression in OT patients have not been established. Certainly, this population develops relative tolerance to the effects of fentanyl but this very potent MOR agonist can still cause death from overdose when administered via IV injection [8-11]. In this study, fentanyl administration will proceed through a dose escalation, beginning at doses in the upper end of the range tolerated by healthy subjects and proceeding cautiously upward based on clinical response (Table 6). Opioid-tolerant patients will be monitored in an anaesthesia suite and stopping criteria will be instituted to turn off opioid infusions and limit dose escalation if a procedure-related AE occurs.

Table 6: Proposed fentanyl doses for healthy subjects and opioid-tolerant patients

Healthy Subjects		Opioid-tolerant Patients	
**ug/kg**	**mg/70 kg**	**ug/kg**	**mg/70 kg**
1.1	0.075		
2.1	0.15		
3.6	0.25	3.6	0.25
5.0	0.35	5.0	0.35
		7.1	0.5
		10.0	0.7

Healthy Subjects		Opioid-tolerant Patients	
		14.3	1.0
		21.4	1.5
		28.6	2.0

If 2 healthy subjects or 2 OT patients receiving the same buprenorphine dose are discontinued due to the same drug-related AE, including procedure-related AEs, then additional subjects will not be included into that buprenorphine dose cohort and higher buprenorphine doses will not be explored in that subject group.

Skeletal muscle rigidity of the truncal area of the body can occur following administration of potent opioids including fentanyl. The effect is centrally mediated (i.e., most probably within the spinal cord) and does not affect ventilatory drive. Risk factors included (1) extremes of age, (2) high dose of rapidly injected (within seconds) opiates, (3) critical illness (metabolic, neurologic disease), and (4) use of drugs that modify dopamine levels (e.g., in Parkinson disease). If rigidity occurs, it will be treated with (1) IV naloxone 100-200 µg (slowly given) or (2) low-dose neuromuscular blocking agent such as rocuronium or succinylcholine.

1.3.7 Treatment duration

Healthy subjects' (Part A) participation is a maximum of 13 weeks; including a 31-day screening period, up to 3 treatment periods (3 days each) and an End of Study/Early Termination Visit (EOS/ET). Each treatment period and the EOS/ET visit will be separated with a washout period of 10 to 17 days.

Opioid-tolerant patients' (Part B) participation is approximately 8 weeks, including a 31-day screening period, 2 treatment periods separated by at least 40 hours and an EOS/ET visit 10 to 17 days after the last treatment period.

Extensions of the protocol visit windows (between screening and Visit 1 and between visits) are allowed, but will be pre-approved by the medical monitor, the Principal Investigator and the co-investigator from the LUMC.

2 STUDY OBJECTIVES

2.1 Primary objective

- To determine if buprenorphine action at the MOR receptor can inhibit the respiratory depression response to IV fentanyl injection in healthy subjects and opioid-tolerant (OT) patients;
- To determine if therapeutic concentrations achieved with administration of buprenorphine in OT patients protect against respiratory depression associated with high concentrations of fentanyl.

2.2 Secondary objective

- To assess safety as determined by AE reporting.

2.3 Exploratory objectives

- To assess safety as determined by concomitant medications, laboratory test results, vital signs, physical examination findings, ECG parameters and Columbia-Suicide Severity Rating Scale (C-SSRS) responses;
- To evaluate pharmacokinetics of buprenorphine during primed-continuous IV infusion;
- To evaluate pharmacokinetics of fentanyl with repeated IV bolus injections;
- To explore changes in ventilation parameters during the IV administration of buprenorphine and fentanyl; and
- To build a model describing the PK/PD interaction between buprenorphine and fentanyl concentrations and their effect on ventilatory parameters.

3 STUDY DESIGN

This study will be performed in 2 parts (Part A and Part B) at 2 clinical study sites. Dosing day procedures will be performed at LUMC, and all other study procedures will be performed at CHDR.

Part A consists of up to 3 dosing periods in healthy subjects. The first two periods will have a single-blind, placebo-controlled, cross-over design; subjects will be assigned in a 1 to 1 ratio to 2 treatment sequences determined by the order in which they receive buprenorphine or placebo in the 2 treatment periods. The same set of fentanyl doses will be administered in both periods. Period 3 will have an open label design, where the same subjects will receive buprenorphine only. Period 3 is optional, and not all subjects are required to participate; the sponsor and investigator collectively determine if Period 3 is required for each subject. In total, about 6 subjects will participate in a third investigational period. Part B is an open label study in OT patients. Opioid-tolerant patients will receive placebo and fentanyl challenges in Period 1, in order to optimise the fentanyl dose escalation before buprenorphine and fentanyl are co-administered in Period 2.

3.1 Overall study design and plan

Healthy subjects' (Part A) participation is a maximum of 13 weeks, divided as follows:

- Screening: Day -31 until Day -2 before the first drug administration;
- Two single-blind, placebo-controlled, cross-over investigational periods of 3 days
- One optional, open-label, buprenorphine-only investigational period of 3 days
- Washout period of 10 to 17 days between periods
- EOS/ET visit: 10 to 17 days after last dose of buprenorphine or placebo

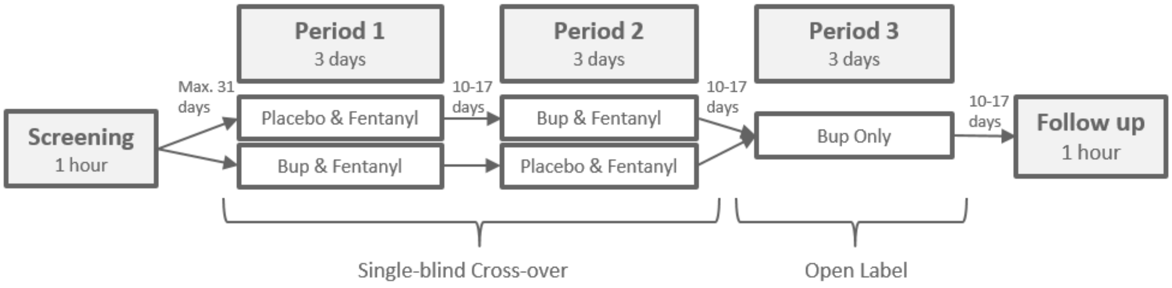

Figure 1: Graphical overview of study design of Part A

Opioid-tolerant patients' (Part B) participation is approximately 8 weeks, divided as follows:

- Screening: Day -31 until Day -3 before the first drug administration
- 2 - 5 day stay at CHDR (From admission to Day -1)
- 1 day LUMC stay with investigational period 1
- Return to CHDR for 1 day for a washout period of at least 40 hours
- 1 day LUMC stay with investigational period 2
- EOS/ET visit: 10 to 17 days after last dose of buprenorphine

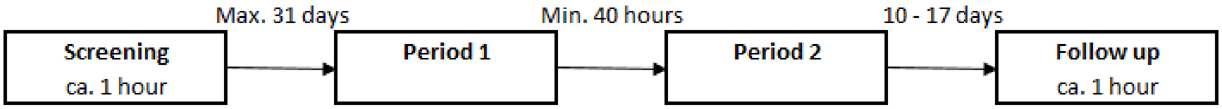

Figure 2: Graphical overview of study design of Part B

All healthy subjects (Part A) and OT patients (Part B) will be screened at CHDR up to 31 days prior to study drug administration. Subjects who sign informed consent and meet all entry criteria may be enrolled.

Healthy subjects in Part A will be studied in 2 or 3 periods, with a 10-17 day interval between the periods. All subjects will receive ondansetron before dosing with buprenorphine or placebo, in order to minimise the nausea that may be caused by opioids. Subjects will receive the same doses of fentanyl challenges in Periods 1 and 2; fentanyl will not be administered in Period 3. Buprenorphine doses will be set per dosing cohort. All subjects will be admitted to the CHDR the evening before each study period, and will be escorted to LUMC the following morning. After study periods are completed, they will be transferred to the PACU for overnight monitoring.

All OT patients in Part B will be studied in 2 periods, with at least 40 hours washout period in between. All OT patients will be transitioned to oral oxycodone at least 48 hours before Period 1, and will be admitted to the CHDR 2 - 5 days before Period 1 in order to ensure washout of the patients' regular opioids and a stable dose with an adequate bridging schedule has been reached. Tolerance to opioid effects is poorly characterised in subjects receiving long-term opioids, therefore OT patients in Part B will receive placebo plus fentanyl challenges in Period 1, in order to optimise the fentanyl dose escalation before the buprenorphine and fentanyl are co-administered in Period 2. Due to the short half-life of fentanyl, OT patients will return to CHDR and will continue onto Period 2 after a washout of at least 40 hours. Opioid-tolerant patients will be escorted to LUMC on the morning of Day 1 and Day 3. In the event that a washout period significantly longer than 40 hours is required between Periods 1 and 2, OT patients may stay at CHDR for a longer period until Period 2 begins.

Details regarding the technique for studying ventilation are presented in Section 7.1.2.

The EOS/ET visit will be completed 10-17 days after the final study period dosing.

Extensions of the protocol visit windows (between screening and Visit 1 and between visits) are allowed, but will be pre-approved by the medical monitor, the Principal Investigator and the co-investigator from the LUMC.

3.1.1 Screening (Location: CHDR)

After informed consent is obtained, all screening procedures designated in the Schedule of Assessments (Table 1 and Table 2) will be performed between Day -31 and admission at CHDR. The entire screening process will last approximately 1 hour. This includes obtaining demographics, medical history, physical examination (including height/weight), recording of vital signs, 12-lead electrocardiogram (ECG), C-SSRS, safety laboratory (biochemistry, haematology, serum pregnancy [for females only], urinalysis dipstick and urine drug screen [UDS]) and alcohol breath test. A drug use history of lifetime and the past 30 days will be captured for OT patients in Part B only. Study eligibility must be met per inclusion/exclusion criteria prior to enrolment.

Subjects in Part A of the study who do not meet the eligibility criteria may not be rescreened.

Rescreening of OT patients in Part B of the study is allowed under the following circumstances, all of which require approval of the Principal Investigator and the medical monitor:

- Opioid-tolerant patients who met all eligibility criteria but had an intercurrent illness (e.g., upper respiratory infection with fever) in the 5 days before the first study drug dose that was properly evaluated and which resolved fully;

- Opioid-tolerant patients who met all eligibility criteria but transiently (for personal reasons) are unable to commit to all study procedures;
- Opioid-tolerant patients who met all eligibility criteria but are not randomised for administrative reasons (e.g., study drug is not available at the study site);
- Opioid-tolerant patients who were screened under a prior version of the protocol and did not meet any exclusion criterion, with the exception of a criterion that was updated in a subsequent version of the protocol.

No repeat of tests is necessary for screening assessments that meet the eligibility criteria that are still within the window.

3.1.2 Treatment and observation period (Location: CHDR/LUMC)

Subjects in Part A will attend the clinic on 2 or 3 separate periods, with a 10-17 day washout between periods. For each investigational period, the subjects will be admitted to CHDR the evening before each study period and will be transferred to the anaesthesia suite at the LUMC the following morning. Opioid tolerant patients in Part B will be admitted to the clinic once, at least 48 hours prior to first study drug administration and will be transferred to the anaesthesia suite at the LUMC on Day 1 and Day 3 where they will stay for both study drug administration days with overnight monitoring on the PACU, with a return visit to CHDR for the washout period. In the event that a washout period significantly longer than 40 hours is required, OT patients may stay at CHDR for a longer period until Period 2 begins.

Subjects and OT patients will be escorted by car from the CHDR to the LUMC by qualified CHDR personnel. Upon arrival at the LUMC, qualified personnel of the LUMC will escort the subjects and OT patients from the car to the anaesthesia suite.

Buprenorphine or placebo, and fentanyl boluses by dose escalation (Table 5 and Table 6), will be administered on Day 1. After the dose administration is completed, they will be transferred to the PACU for overnight monitoring.

Safety, pharmacokinetic and pharmacodynamic assessments will be performed at time points specified in the Schedule of Assessments (Table 1 - 4). Subjects/OT patients will remain in-house until all study procedures are complete and adequate spontaneous breathing is established and maintained.

Dose and Duration of Treatment:

Study drug administration of buprenorphine or placebo will start after a 30 to 45-minute baseline ventilation period. Buprenorphine/placebo will be administered for 6 hours. Fentanyl boluses will be given by dose escalation +2HR, +3HR, +4HR and +5HR after starting administration of buprenorphine/placebo. Dose administration of buprenorphine/placebo will be done via a calibrated syringe pump. Fentanyl boluses will be administered by a qualified member of nursing (or medical) staff.

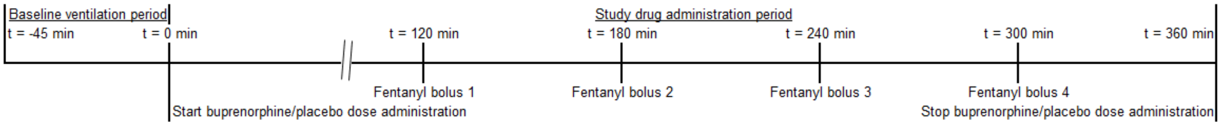

Figure 3: Graphical overview of the study drug administration periods in Part A and Part B. In Part A, buprenorphine and placebo are randomised. In Part B, placebo dosing will occur in Period 1, and buprenorphine dose administration will occur in Period 2.

Part A: Healthy subjects

All healthy subjects will be dosed with 4 mg ondansetron at least 15 minutes before dosing with buprenorphine or placebo. Buprenorphine dosing is flexible and infusion rates will be selected to target approximately 25 to 50% respiratory depression. The potential buprenorphine continuous infusion rates, based on published reports providing a dose-response for buprenorphine effects on analgesia and ventilation [12, 13, 27], include 0.005, 0.01, 0.02, 0.05, 0.1 mg and 0.2 mg/70 kg/h.

The initial dose cohort of 3 subjects will receive the starting dose of buprenorphine listed below and subsequent doses will be selected to explore the full range of effects on ventilation. The starting dose of buprenorphine is expected to yield significant receptor occupancy and produce differential effects on respiratory depression relative to placebo. Doses for subsequent dose cohorts will be selected from the potential doses listed based on subject tolerability and respiratory effects from earlier dose cohorts.

At a minimum, the buprenorphine PK samples will be analysed after the first dose cohort, to confirm the target buprenorphine concentration of 0.5 ng/mL is achieved.

The safety and tolerability data will be reviewed, at a minimum, after each cohort of 3 subjects. If 2 subjects in dose-cohort 1 are discontinued due to the same drug related AE, including procedure-related AEs, then additional subjects will not be included into that buprenorphine dose cohort and higher buprenorphine doses will not be explored. The anticipated effects of buprenorphine infusion are an approximate 50% decrease from baseline minute ventilation with additional respiratory depression < 25% with fentanyl administration.

If anticipated effects on minute ventilation or required PK exposures are not observed, the next higher buprenorphine dose will be administered in the following dose cohort. Otherwise, lower doses will be administered in subsequent dose cohorts to more fully describe the exposure-response relationship for buprenorphine. Table 5 presents the range of anticipated buprenorphine doses.

Starting buprenorphine dose: target concentration 0.5 ng/mL

- 0.125 mg/70 kg bolus
- 0.05 mg/70 kg/h for 360 minutes

Healthy subjects will be dosed with 90-second fentanyl bolus injections as follows:

- Bolus 1: 0.075 mg/70 kg
- Bolus 2: 0.15 mg/70 kg (hold if apnoea observed in earlier steps)
- Bolus 3: 0.25 mg/70 kg (hold if apnoea observed in earlier steps)
- Bolus 4: 0.35 mg/70 kg (hold if apnoea observed in earlier steps)

Part B: Opioid-tolerant patients

Opioid-tolerant patients in Part B will undergo a washout of their own opioids during which these will be replaced with oral oxycodone, and continue at stable doses of oxycodone from at least 48 hours before Period 1 to the end of Period 2. The last pre-study dose of oxycodone will be administered no less than 15 hours before expected study drug administration. Placebo and fentanyl boluses will be administered during the first study period to permit dose escalation to full respiratory effects of fentanyl before assessing the interaction with buprenorphine during the second study period. The low and high doses of buprenorphine have been shown to achieve 50% and > 80% receptor occupancy measured by positron emission tomography with [^{11}C]-carfentanil radioligand [14, 15]. Buprenorphine doses can be adjusted as needed up to a maximum infusion rate of 0.75

mg/70 kg/h based on experimental observations in Part A, indicating that higher doses of buprenorphine are required to shift the respiratory depression response to IV fentanyl injection to the right.

Low Dose: target concentration 1.0 ng/mL

- 0.25 mg/70 kg bolus

0.1 mg/70 kg/h for 360 minutes

High Dose: target concentration 5.0 ng/mL

- 1.25 mg/70 kg bolus
- 0.5 mg/70 kg/h for 360 minutes

Chronic opioid administration via prescription opioids can elicit marked tolerance to opioid effects, therefore fentanyl bolus dose escalation will be performed on an individual basis. If the initial fentanyl boluses have no visible impact on ventilation, then the escalation will push beyond the doses listed below. The potential doses include 0.25, 0.35, 0.5, 0.7, 1.0, 1.5 and 2.0 mg/70 kg. Each dose escalation of fentanyl will be based on observations during the prior bolus. If respiratory effects of fentanyl are observed with the administered dose, the next bolus will be administered as planned (Table 6). If no visible impact on respiration is observed, the escalation will skip to the next higher fentanyl dose. For example, if no effects are evident with the initial 0.25 mg/70 kg bolus, Bolus 2 will be increased to 0.5 mg/70 kg. If no effects are observed at this dose, then Bolus 3 will be increased to 1.0 mg 70 kg. If minute ventilation does not fall below 2/3 of baseline at the maximum dose of fentanyl, then this patient will not be continued into the second period and will be replaced.

- Bolus 1: 0.25 mg/70 kg
- Bolus 2: 0.35 mg/70 kg (hold if apnoea observed in earlier steps)
- Bolus 3: 0.5 mg/70 kg (hold if apnoea observed in earlier steps)
- Bolus 4: 0.7 mg/70 kg (hold if apnoea observed in earlier steps)

Details regarding the technique for studying ventilation, are presented in Section 7.1.2.

3.1.3 End of Study/Early Termination Visit (Location: CHDR)

Subjects and OT patients will return to the CHDR 10-17 days following the last dose of buprenorphine or placebo for an EOS/ET visit. At this visit, the following procedures will be completed: physical examination, review of AEs and concomitant medication, collect blood and urine samples for safety laboratory tests including a urine pregnancy test for females, obtain 12-lead ECG measurement and assess supine blood pressure, pulse rate and respiratory rate.

4 STUDY POPULATION

4.1 Subject population

Part A (Healthy subjects):
Subjects will be recruited via media advertisement or from the subjects' database of the CHDR, Leiden, The Netherlands.

Part B (Opioid-tolerant patients):
Opioid-tolerant patients will be recruited via media advertisement. Recruitment efforts will also focus on OT patients using prescription opioids.

4.2 Inclusion criteria

Subjects must meet all of the following inclusion criteria.

Part A (Healthy subjects):

1. Signed the informed consent form (ICF) and able to comply with the study requirements and restrictions listed therein;
2. Male and female subjects, age 18 to 45 years, inclusive;
3. Women of childbearing potential (defined as all women who are not surgically sterile or postmenopausal for at least 1 year prior to informed consent) must have a negative serum pregnancy test prior to enrolment and must agree to use a medically acceptable means of contraception from screening through at least 3 months after the last dose of study drug;
4. Body Mass Index (BMI) 18 to 30 kg/m^2, inclusive;
5. Healthy as defined by the Investigator, based on a medical evaluation that includes the subject's medical and surgical history, physical examination, vital signs, 12-lead ECG, haematology, blood chemistry, and urinalysis;
6. No history of substance use disorder;
7. No current use of any CNS depressants prescribed or otherwise.

Part B (Opioid-tolerant patients):

1. Signed the ICF and able to comply with the requirements and restrictions listed therein;
2. Males or females age 18 to 55 years, inclusive;
3. Women of childbearing potential (defined as all women who are not surgically sterile or postmenopausal for at least 1 year prior to informed consent) must have a negative serum pregnancy test prior to enrolment and must agree to use a medically acceptable means of contraception from screening through at least 3 months after the last dose of study drug;
4. BMI 18 to 32 kg/m^2, inclusive;
5. Opioid-tolerant patients administered opioids at daily doses ≥ 90 mg oral morphine equivalents (See Appendix A);
6. Stable as defined by the Investigator, based on a medical evaluation that includes the subject's medical and surgical history, physical examination, vital signs, 12-lead ECG, haematology, blood chemistry, and urinalysis;
7. No use of any CNS depressants, besides opioids, prescribed or otherwise in a period equal to 5 half-lives of the product before first study drug administration unless assessed as safe by the principal investigator.

4.3 Exclusion criteria

Subjects must not meet any of the following exclusion criteria.

Part A (Healthy subjects):

1. History of risk factors of Torsades de Pointes (e.g. heart failure, hypokalaemia, family history of Long QT Syndrome) or an ECG demonstrating a Fridericia's corrected QT interval (QTcF) > 450 msec in males and QTcF > 470 msec in females at screening;
2. Currently meet the criteria for diagnosis of substance use disorder according to the Diagnostic and Statistical Manual of Mental Disorders (DSM)-5 criteria on any substance;
3. Any other active medical condition, organ disease or concurrent medication or treatment that may either compromise subject safety or interfere with study endpoints (includes sleep apnoea, other significant respiratory illness, history or risk of difficult intubation, limited cervical spine mobility or limited oral excursion);
4. Current smokers and those who have smoked within the last 6 months;
5. Current users of e-cigarettes and nicotine replacement products and those who have used these products within the last 6 months;
6. Consume, on average, > 20 units/week of alcohol in men and > 13 units/week of alcohol in women (1 unit = 1 glass (250 mL) beer, 125 mL glass of wine or 25 mL of 40% spirit);
7. Previous treatment with any prescribed medications (including all type of vaccines) or over-the-counter (OTC) medications (including homeopathic preparations, vitamins, and minerals) within 14 days or 5 half-lives (whichever is longer) prior to first study treatment administration;
8. Previous or current treatment with opioid agonist, partial agonist, or antagonist treatment within 30 days prior to the first study drug administration;
9. Require ongoing prescription or OTC medications that are clinically relevant CYP P450 3A4 or CYP P450 2C8 inducers or inhibitors (e.g., rifampicin, azole antifungals [e.g., ketoconazole], macrolide antibiotics [e.g., erythromycin]);
10. Significant traumatic injury, major surgery, or open biopsy within the prior 4 weeks of informed consent;
11. History of suicidal ideation within 30 days prior to informed consent or history of a suicide attempt in the 6 months prior to informed consent;
12. Measured systolic blood pressure greater than 160 or less than 95 mmHg or diastolic pressure greater than 95 mm Hg prior to Day 1;
13. History or presence of allergic response to buprenorphine or fentanyl;
14. Subjects who have demonstrated allergic reactions (e.g., food, drug, atopic reactions or asthmatic episodes) which, in the opinion of the Investigator and sponsor, interfere with their ability to participate in the trial;
15. Estimated glomerular filtration rate < 60 mL/min as estimated by the Chronic Kidney Disease Epidemiology Collaboration (CKD-EPI) equation;
16. Anaemia at screening or donation of > 250 mL of blood or plasma within the last 3 months;
17. Positive serology tests for HIV, hepatitis B (HBsAg), or hepatitis C (HepC);
18. Aspartate transaminase (AST) or alanine transaminase (ALT) levels > 1.5 times the upper limit of normal at screening;
19. Treatment with another investigational drug within 3 months prior to dosing or having participated in more than 4 investigational drug studies within 1 year prior to screening;
20. Site staff or subjects affiliated with, or a family member of, site staff directly involved in the study.

Part B (Opioid-tolerant patients):

1. Clinically significant risk factors of Torsades de Pointes (e.g., heart failure, hypokalaemia, family history of Long QT Syndrome) or an ECG demonstrating a Fridericia's corrected QT interval (QTcF) > 450 msec in males and QTcF > 470 msec in females at screening;
2. Currently meet the criteria for diagnosis of moderate or severe substance use disorder according to the DSM-5 criteria on any substances other than opioids, caffeine, or nicotine;

3. Any active medical condition, organ disease or concurrent medication or treatment that may either compromise subject safety or interfere with study endpoints (includes sleep apnoea, other significant respiratory illness, history or risk of difficult intubation, limited cervical spine mobility or limited oral excursion);
4. Not able to abstain from smoking cigarettes during each dosing day;
5. Consume, on average, > 27 units/week of alcohol in men and > 20 units/week of alcohol in women (1 unit = 1 glass (250 mL) beer, 125 mL glass of wine or 25 mL of 40% spirit);
6. Use of buprenorphine within 10 days of the first study drug administration;
7. Require ongoing prescription or OTC medications that are clinically relevant CYP P450 3A4 or CYP P450 2C8 inducers or inhibitors (e.g., rifampicin, azole antifungals [e.g., ketoconazole], macrolide antibiotics [e.g., erythromycin]);
8. Significant traumatic injury, major surgery, or open biopsy within the prior 4 weeks of informed consent;
9. History of suicidal ideation within 30 days prior to informed consent or history of a suicide attempt in the 6 months prior to informed consent;
10. Measured systolic blood pressure greater than 160 or less than 95 mmHg or diastolic pressure greater than 95 mmHg prior to Day 1;
11. History or presence of allergic response to buprenorphine or fentanyl;
12. Opioid-tolerant patients who have demonstrated allergic reactions (e.g., food, drug, atopic reactions or asthmatic episodes) which, in the opinion of the Investigator and sponsor, interfere with their ability to participate in the trial.
13. Estimated glomerular filtration rate < 60 mL/min as estimated by the CKD-EPI equation;
14. Anaemia at screening or donation of > 250 mL of blood or plasma within the last 3 months;
15. Positive serology tests for HIV, acute hepatitis B, or acute hepatitis C (patients with asymptomatic hepatitis B or C infection may be enrolled);
16. AST or ALT levels >3.0 times the upper limit of normal at screening;
17. Treatment with another investigational drug within 3 months prior to dosing or having participated in more than 4 investigational drug studies within 1 year prior to screening;
18. Site staff or subjects affiliated with, or a family member of, site staff directly involved in the study.

4.4 Concomitant medications

All medications (prescription and OTC) taken within 30 days of study screening will be recorded with indication, route of administration, daily dose, and start and stop dates of administration. All subjects will be questioned about concomitant treatment at each clinic visit.

4.4.1 Allowed concomitant medications

Part A (Healthy subjects):

Subjects may be provided rescue medication for AEs such as pain, vomiting and allergic reactions, as necessary. Paracetamol can be given up to 4 g/day and ibuprofen 1.2 g/day. Exceptions will only be made if the rationale is clearly documented by the Investigator.

Part B (Opioid-tolerant patients):

Decisions about concomitant medication of OT patients requiring medication for treated disease(s) will be made jointly by the Investigators and sponsor. OT patients may be provided rescue medication for AEs such as vomiting and allergic reactions. Breakthrough pain can be managed by doses of ibuprofen (1.2 g/day) and paracetamol (4 g/day).

4.4.2 Prohibited concomitant medications

Part A (Healthy subjects):

No prescription medications and OTC medications will be permitted within 14 days prior to study drug administrations, or less than 5 half-lives, whichever is longer, and during the course of the study. In addition, no vitamin, mineral, herbal, and dietary supplements will be permitted within 7 days prior to study drug administrations, or less than 5 half-lives, whichever is longer, and during the course of the study. A history or current treatment with an opioid agonist, partial antagonist or antagonist within 30 days prior to screening will not be permitted. Rescue medications cannot include CNS depressants and clinically relevant CYP P450 3A4 or CYP P450 2C8 inducers or inhibitors (e.g., rifampicin, azole antifungals [e.g., ketoconazole], macrolide antibiotics [e.g., erythromycin]).

Part B (Opioid-tolerant patients):
Use of buprenorphine within 10 days of the first study drug administration will be prohibited. Prohibited concomitant medication are CNS depressants (besides opioids) and clinically relevant CYP P450 3A4 or CYP P450 2C8 inducers or inhibitors (e.g., rifampicin, azole antifungals [e.g., ketoconazole], macrolide antibiotics [e.g., erythromycin]). Any of the prohibited medications must be discontinued 14 days or 5 half-lives, whichever is longer, prior to study drug administration and cannot be restarted until 48 hours after the final study drug administration, unless assessed as safe to be administered throughout the study by the principal investigator. Subjects who are not able to abstain from smoking on the dose administration days without supplementary nicotine, will be provided adequate nicotine supplements (e.g. nicotine-patch, nicotine-containing chewing gum) that do not interfere with the procedures conducted on the study days.

4.4.3 Bridging medication

Part B (Opioid-tolerant patients):
For OT patients, a case-by-case bridging schedule with oral oxycodone will be prescribed from at least 48 hours until not less than 15 hours prior to study drug administration. The oral oxycodone should not interfere with procedures conducted on the study days. The case-by-case bridging schedules will be made by CHDR in collaboration with the department of anaesthesiology of LUMC.

Between Period 1 and 2, the patient will return to their oxycodone dosing schedule. After Period 2 has finished, the patient will return to their original medication schedules.

4.5 Lifestyle restrictions

- For the screening and the EOS/ET visit, subjects will be required to fast for at least 4 hours. Subjects will be required to fast from 24:00 hours on Day -1 (no food and no fluid including water);
- Any nutrients known to modulate CYP enzymes activity (e.g., grapefruit or Seville orange containing products or quinine-containing drinks [tonic water or bitter lemon]) will not be permitted from 7 days before dosing until discharge from the PACU;
- Poppy seed or foods containing poppy seeds are not permitted from 3 days before dosing and until discharge from the PACU;
- Alcohol will not be allowed from at least 24 hours before each scheduled visit (screening, dosing and whilst in the study unit until discharge from the PACU). Subjects may undergo an alcohol breath test at the discretion of the Investigator;
- Subjects/OT patients will not be allowed to have excessive caffeine consumption, defined as > 800 mg per day from 7 days prior to the first dose of the study drug until discharge from the PACU after Period 2. Caffeine quantities defined as: one cup of coffee contains 100 mg of caffeine; one cup of tea, or one glass of cola, or potion of chocolate (dark:100 g, milk 200 g) contains approximately 40 mg of caffeine; one bottle of Red Bull contains approximately 80 mg of caffeine;
- Strenuous physical activity (e.g., heavy lifting, weight or fitness training) is not allowed from 48 hours prior to each study day until discharge from the PACU. Light ambulatory activities (e.g. walking at normal pace) will be permitted, with the level of activities kept as similar as possible on all days in the study unit.

4.5.1 Contraception requirements

All women of childbearing potential and all males must use, with their partner, an approved method of highly effective contraception from the time of consent until 3 months after the last dose of study medication.

Female subjects who are not of childbearing potential do not need to use any methods of contraception.

A female is considered of childbearing potential unless post-menopausal or permanently sterile. Permanent sterilisation methods include hysterectomy, bilateral salpingectomy and bilateral oophorectomy. A post-menopausal state is defined as no menses for 12 months without an alternative medical cause and confirmed by a follicle stimulating hormone (FSH) result of ≥ 40 IU/L.

For the purposes of the study, effective contraception is defined as follows:

- Combined (oestrogen and progestogen-containing) hormonal contraception associated with inhibition of ovulation including oral, intravaginal and transdermal preparations;
- Progestogen-only hormonal contraception associated with inhibition of ovulation including oral, injectable, implantable or intrauterine systems for administration;
- Intrauterine device;
- Surgical sterilisation (for example, vasectomy or bilateral tubal ligation).

Males: Effective male contraception includes a vasectomy with negative semen analysis at follow up, or the use of condoms with spermicide.

Alternatively, true abstinence is acceptable when it is in line with the subject's preferred and usual lifestyle. If a subject is usually not sexually active but becomes active, they, with their partner, must comply with the contraceptive requirements detailed above.

4.6 Study drug discontinuation and withdrawal

4.6.1 Study drug interruption or discontinuation

Before study medication is administered, changes in the subject's health status (including laboratory results, if applicable) since the previous visit must be checked. The Investigator must temporally interrupt or permanently discontinue the study drug if continued administration of the study drug is believed to be contrary to the best interests of the subject. The interruption or premature discontinuation of study drug might be triggered by an AE, a diagnostic or therapeutic procedure, an abnormal assessment (e.g., ECG or laboratory abnormalities), or for administrative reasons, in particular, withdrawal of the subject's consent.

A procedure-related AE is defined by loss of respiratory activity for 60 seconds or longer, despite active stimulation of the subject, end-tidal partial pressure of carbon dioxide greater than 67.5 mmHg, O_2 saturation less than 85% for at least 2 minutes, or any other situation or condition that may interfere with the health of the participant. If investigators stimulate the subjects to breathe or give supplemental oxygen as needed to prevent an AE, the subject will not proceed to the next fentanyl dose and the study session will terminate early. If 2 subjects in dose-cohort 1 are discontinued due to the same drug related AE, including procedure-related AEs, then additional subjects will not be included into that buprenorphine dose cohort and higher buprenorphine doses will not be explored. The reason for study drug interruption or premature discontinuation must be documented.

4.6.2 Subject withdrawal

Subjects have the right to withdraw from the study at any time for any reason. If a subject indicates that he or she wants to discontinue the experiment or in case of an AE, all infusions will be discontinued for that session and the individual will be treated according to established ventilatory support and opioid reversal protocols.

Should a subject decide to withdraw from the study, all efforts should be made to complete and report the observations, particularly the EOS/ET examinations, as thoroughly as possible.

4.6.3 Replacement policy

Subjects withdrawing for any reasons may be replaced. Decisions will be made jointly by the Investigator and sponsor.

5 INVESTIGATIONAL MEDICINAL PRODUCT

5.1 Investigational drug and matching placebo

- Buprenorphine (Temgesic®) 0.3 mg/mL or placebo 0.9% normal saline for IV injection.
- Fentanyl 0.05 mg/mL for IV injection.

An overview of the intended dose levels is given in Table 5 and Table 6.

Non-investigational drug:

- Ondansetron 2 mg/mL for IV injection
- Oxycodone tablets (for Part B only)

5.2 Study drug packaging and labelling

The buprenorphine and fentanyl will be sourced from the local study pharmacy. Upon arrival at the pharmacy, the investigational products should be checked for damage, and proper identity, quantity, integrity of seals and temperature conditions verified; report any deviations or product complaints upon discovery. The dispensing of the study drug will be performed by the pharmacy. Study drug will be dispensed for each subject according to the randomisation list. Study drug packaging will be overseen by the LUMC pharmacy and will bear a label with the identification required by local law, the protocol number, drug identification and dosage.

Efforts should be made to source study drug for administration from a single source and batch. The study pharmacist will record the following information in the case report form: branded or generic, manufacturer and batch number.

5.3 Drug accountability

Drug accountability will be maintained by the LUMC pharmacy and assessed by maintaining adequate study drug dispensing records.

The Investigator is responsible for ensuring that dosing is administered in compliance with the protocol. Delegation of this task must be clearly documented and approved by the Investigator. All study drug administration will occur under medical supervision.

In case investigational or non-investigational drugs will not be administered to a subject or patient for whatever reason, the drug will be returned to the LUMC pharmacy and documented according standard operating procedure (SOP) CGEDRADM.

5.4 Treatment assignment and blinding

5.4.1 Randomisation and treatment assignment

<u>Part A (Healthy subjects):</u>
Subjects will be randomised to 1 of the 2 treatment sequences (buprenorphine vs. placebo) in a 1 to 1 ratio for Periods 1 and 2. Subjects must be randomised in a consecutive order starting with the lowest number.

The randomisation code will be generated using SAS version 9.4 (or a more recent version if available) by a study-independent CHDR statistician.

A consecutive assignment approach will be used to assign the eligible subjects to the 6 dose cohorts. For example, the first 3 consecutive eligible subjects will be assigned to dose-cohort 1, and the second 3 consecutive eligible subjects will be assigned to dose-cohort 2.

The dose-response for the buprenorphine-fentanyl interaction has not been previously explored; therefore, it may be necessary to include up to 6 additional enrolled subjects to fully characterise the range of buprenorphine

doses and/or the fentanyl-buprenorphine drug-drug interaction. The subjects will be assigned to an additional 2 dose cohorts, in the same manner as above.

Subjects will be numbered in a consecutive order. Dose-cohort 1 will be numbered starting at 101, dose-cohort 2 will be numbered starting at 104, cohort 3 will be numbered starting at 107, etc. Replacement subjects will be numbered +1000; e.g., Subject 105 will have Subject 1105 as a replacement.

Part B (Opioid-tolerant patients):
Opioid-tolerant patients will be numbered starting at 201. Replacement subjects will be numbered +1000; e.g., Subject 205 will have Subject 1205 as a replacement.

5.4.2 Blinding

The first two periods of Part A of this study will be performed in a single-blind fashion. Only the subjects will remain blinded to the treatment sequence (order of dose administration buprenorphine/placebo), while investigators and sponsor personnel will be aware of subject's treatment sequence assignment after each subject is randomised to a sequence of the treatments. The investigational drug and its matching placebo are indistinguishable and will be packaged in the same way by the LUMC pharmacy.

Part A Period 3 and Part B will be performed as an open-label study; therefore, blinding is not required.

6 STUDY ENDPOINTS

6.1 Primary endpoint

Minute ventilation (L/min), respiratory rate (/min), oxygen saturation (SpO_2), tidal volume (L), end-tidal P_{CO2} (kPa; PE^{i}_{CO2}) and end-tidal P_{O2} (kPa; PE^{i}_{O2}) will be measured for each breath during the baseline period and during infusion of study drugs.

- Peak ventilatory depression (change in minute ventilation) will be calculated based on a 1-minute average of the ventilation data of each individual subject/patient. For buprenorphine or placebo, absolute changes and percentage changes will be calculated from the baseline value. For fentanyl, absolute changes and percentage changes for each bolus will be calculated from the baseline value and from the pre-fentanyl Baseline value immediately before the first fentanyl bolus.

The baseline value is the minute ventilation value when a stable ventilation pattern is established for at least 2 minutes prior to the infusion of buprenorphine/placebo. The pre-fentanyl baseline value is defined as the minute ventilation value averaged between the timepoint when a stable ventilation pattern is established for at least 2 minutes after the start of buprenorphine/placebo infusion and the timepoint before the first fentanyl bolus.

6.2 Secondary endpoints

For Part A (Healthy subjects):

- Number (percentage) of subjects who experience apnoea for each fentanyl dose during the placebo treatment vs. the buprenorphine treatment. Apnoea is defined as a 20-second pause in respiration. If apnoea is observed at any fentanyl dose for a subject, that subject will be classified as 'experienced apnoea' for that dose, and any higher fentanyl dose planned in the study (the next higher fentanyl dose or doses will be withheld); and
- Number (percentage) of subjects who require stimulation for breathing for each fentanyl dose during the placebo treatment vs. the buprenorphine treatment. If a subject required stimulation for breathing at a fentanyl dose and the next higher fentanyl dose or doses was withhold, the subject will also be classified as requiring breath stimulation for the withheld higher fentanyl dose/doses.

For Part B (Opioid-tolerant patients):

- Whether the subject experiences apnoea during buprenorphine treatment at the fentanyl dose at which the subject had apnoea during the placebo treatment; and

Fentanyl dose corresponding to the occurrence of apnoea during placebo and buprenorphine infusion periods (if applicable).

6.3 Exploratory endpoints

6.3.1 Safety and tolerability

- Treatment-emergent (serious) adverse events ([S]AEs)
- Concomitant medications
- Clinical laboratory tests (absolute values and changes from baseline)
  - Haematology
  - Chemistry
  - Urinalysis
- Vital signs (absolute values and changes from baseline)
  - Pulse rate (beats per minute)
  - Systolic blood pressure (mmHg)

- Diastolic blood pressure (mmHg)
- Respiratory rate (breaths per minute)
- Temperature (°C)

- ECG parameters
  - Heart rate (HR) (beats per minute), PR, QRS, QT, QTcF, changes in rhythm
- Physical examination

Columbia-Suicide Severity Rating Scale (C-SSRS)

6.3.2 Pharmacokinetic

The following endpoints will be determined for buprenorphine following each infusion. They will be derived by non-compartmental analysis of the plasma concentration-time data:

- The maximum plasma concentration at the end of the bolus (C_{max});
- The area under the plasma concentration-time curve from zero to t of the last measured concentration above the limit of quantification ($AUC_{0\text{-}last}$);
- The average concentration during the fentanyl dose escalation C_{avg} [2-6 h], which is calculated as AUC[2-6 h]/4 h.

The following endpoints will be determined for fentanyl following each bolus (as appropriate). They will be derived by non-compartmental analysis of the plasma concentration-time data:

- The area under the plasma concentration-time curve for each dosing interval ($AUC_{0\text{-}tau}$);
- The maximum plasma concentration for each dosing interval (C_{max});
- The time to reach maximum plasma concentration for each dosing interval (t_{max});
- Other parameters, including apparent volume of distribution (Vz/F), apparent clearance (CL/F), and other parameters as appropriate, as well as dose adjusted parameters, may be determined.

6.3.3 Pharmacodynamic

Minute ventilation (L/min), respiratory rate (/min), tidal volume (L), oxygen saturation (SpO2, end-tidal P_{CO2} (kPa; PE^{i}_{CO2}) and end-tidal P_{O2} (kPa; PE^{i}_{O2}) are measured for each breath during the baseline period and during infusion of study drugs. The following parameters will be calculated:

- Peak changes in other ventilation parameters will be calculated for buprenorphine or placebo; absolute changes and percentage changes will be calculated from the baseline value. For fentanyl, absolute changes and percentage changes for each bolus will be calculated from the baseline value and from the pre-fentanyl baseline value immediately before the first fentanyl bolus.
- Area under the curve in ventilation parameters will be calculated based on a 1-minute average of the ventilation data of each individual subject/patient. For buprenorphine or placebo, changes will be calculated from the baseline value. For fentanyl, changes for each bolus will be calculated from the baseline value and from the pre-fentanyl baseline value immediately before the first fentanyl bolus.
- When possible, time to peak effect (min) and time to end of effect (i.e., return to baseline in minutes) will be calculated for each for the initial buprenorphine/placebo period and each fentanyl bolus.
- EC_{50} and E_{max} for buprenorphine and fentanyl effects on minute ventilation as determined by pharmacokinetic-pharmacodynamic (PK/PD) models.

- Sedation Visual Analogue Scale (VAS) administered before the first fentanyl bolus and at the conclusion of each bolus period will be evaluated.

7 STUDY ASSESSMENTS

See Table 1 – 4 for the time points of the assessments.

7.1 Pharmacodynamic assessments and questionnaires

7.1.1 Concomitant medications

Concomitant medications initiated, stopped, up-titrated or down-titrated for an AE will be recorded.

7.1.2 Ventilation Monitoring

To study ventilation, the dynamic end-tidal forcing (DEF) technique will be used [12, 13]. This technique enables the Investigator to force end-tidal PCO_2 and end-tidal PO_2 to follow a specific pattern in time. End-tidal PCO_2 and PO_2 will be clamped to approximately 7 and 14.5 kPa, respectively, until minute ventilation (VE) reaches 20 to 24 L/min. Subjects breathe through a face mask and receive fresh gas (45 L/min) with oxygen, carbon dioxide and nitrogen adjusted to obtain the desired end-tidal concentrations. The inspired and expired gas flows are measured using a pneumotachograph and the oxygen and carbon dioxide concentrations are measured using a gas monitor; a pulse oximeter continuously measures the oxygen saturation of arterial haemoglobin with a finger probe. Minute ventilation, oxygen saturation (SpO_2), end-tidal P_{CO2} (PE^{i}_{CO2}) and end-tidal P_{O2} (PE^{i}_{O2}) will be stored on a breath-to-breath basis and on an average ventilation of 1 minute during baseline period and during the full period of infusion of buprenorphine/placebo. Because study subjects could experience discomfort maintaining rapid ventilation of 20 to 24 L/min for a sustained period of time, the Investigator has the discretion to remove the ventilation face mask between 30 minutes and 105 minutes during the buprenorphine/placebo infusion period. The face mask must be replaced in time for the subject ventilation to stabilise at the target VE before starting the fentanyl boluses.

After baseline ventilation stabilises (30 to 45 minutes), subjects (all healthy subjects and OT patients as needed) will receive ondansetron 4 mg IV and a primed-continuous IV buprenorphine (or placebo) infusion will be initiated at doses expected to achieve target concentrations resulting in approximately 25% to 50% suppression of baseline minute ventilation. Buprenorphine infusion will continue for 6 hours (360 minutes); total and fentanyl boluses will begin at 120 minutes and will be increased at 180, 240 and 300 minutes to complete a 4-step IV bolus protocol. A second dose of ondansetron 4 mg IV can be administered as needed for management of nausea and vomiting. Infusions of buprenorphine and fentanyl conclude at 360 minutes, and study subjects will be monitored for a minimum of 3 hours before transfer to the PACU.

If a subject indicates that he or she wants to discontinue the experiment or in case of an AE, all infusions will be discontinued for that period and the individual will be treated according to established ventilatory support and opioid reversal protocols. A procedure-related AE is defined by loss of respiratory activity for 60 seconds or longer, despite active stimulation of the subject; end-tidal partial pressure of carbon dioxide greater than 67.5 mmHg; O_2 saturation less than 85% for at least 2 minutes; or any other situation or condition that may interfere with the health of the participant. If investigators stimulate the subjects to breathe or give supplemental oxygen as needed to prevent an AE, the subject will not proceed to the next fentanyl dose and the period will terminate early. Other study drug interruption or discontinuation criteria are listed in Section 4.6.1.

Inhaled gas concentrations are steered and ventilation is measured on a breath-to-breath basis using the DEF technique. This technique allows the manipulation of inspired gas concentrations to steer the end-tidal concentrations of CO_2 and O_2 independent of the ventilatory response or the gas concentrations in mixed venous blood. By varying the inspired CO_2 concentration, the end-tidal PCO_2 is maintained at a fixed elevated level. The target of the end-tidal PCO_2 is not fixed between subjects but determined separately in each subject. The target is such that ventilation level is increased to 20 L/min (± 10%). Throughout the study, the end-tidal PO_2 will be maintained at 110 mmHg, (i.e., a normoxic value such that oxygen saturation will be > 96%).

The DEF system being used consists of hardware and software. The hardware consists of 3 computer-controlled mass flow controllers for oxygen, nitrogen and carbon dioxide; the software is locally manufactured and has 2 components, RRDP and ACQ, developed by [redacted]

7.1.3 Sedation Visual Analog Scale

A VAS question for sedation is in Appendix D (English version). The subject/patient must complete the question on paper during Period 1 and Period 2 before buprenorphine/placebo administration, before the first fentanyl bolus and 1 hour after each bolus (120, 180, 240, 300 and 360 minutes).

The Sedation VAS responses will be recorded on paper source and will then be entered into the subject's case report form (CRF).

7.2 Safety and tolerability assessments

The definitions, reporting and follow-up of AEs, SAEs and potential pregnancies are described in Section 8.

7.2.1 Vital signs

Evaluations of the vital signs, including blood pressure (systolic and diastolic), pulse rate, respiratory rate, temperature (tympanic) and saturation, will be performed throughout the study. Vital signs will be taken after 5 minutes in the supine position. Blood pressure should be measured with the subject comfortably seated, with legs uncrossed, and the back and arm supported. An appropriate-sized cuff should be placed on the upper arm. The arm should be supported at heart level (mid-sternum). Clothing that covers the arm should be removed. For each subject, the same arm will be used for each of that subject's measurements throughout the study. Temperature will be measured after the vital sign assessment.

Automated oscillometric blood pressures and pulse rate will be measured using a Dash 3000, Dash 4000, Dynamap 400 or Dynamap ProCare 400 when measurements are performed at CHDR. When blood pressure and pulse rate are measured at the LUMC a Philips SureSigns VS3 will be used.

7.2.2 Weight and height

Height (cm) will be recorded at screening only. For Part A, weight (kg) will be recorded at screening, during Day -1 of each period at CHDR and on Day 1 of each period at LUMC, and the EOS/ET Visit. For Part B, weight (kg) will be recorded at screening, during Day -1 of Period 1 and on Day 2 between Period 1 and 2 at CHDR, and the EOS/ET Visit.

BMI will be calculated at Screening and EOS/ET visit.

BMI (kg/m^2) = weight (kg)/(height [cm]/100)^2

7.2.3 Physical examination

A physical examination of all body systems will be performed at the Screening Visit and the EOS/ET Visit. At other visits (Period 1 and 2), a symptom-directed physical examination will be performed.

A physical examination of all body systems includes a review of the following systems: head/neck/thyroid, eyes/ears/nose/throat (EENT), respiratory, cardiovascular, lymph nodes, abdomen, skin, musculoskeletal and neurological. Breast, anorectal and genital examinations will be performed when medically indicated. After screening, any clinically significant abnormal findings in the physical will be reported as AEs.

Clinically relevant findings that are present prior to study drug initiation must be recorded with the subject's medical history. Clinically relevant findings found after study drug initiation and meeting the definition of an AE (new AE or worsening of previously existing condition) must be recorded.

7.2.4 Standard 12-lead electrocardiograms

Standard 12-lead ECGs will be performed according to the Schedule of Assessments (Table 1 – 4). Additional standard 12-lead ECGs will be performed at any other time if clinically indicated. The performance of all ECGs will adhere to the following guidelines:

- The subject will be instructed to rest in the supine position for at least 5 minutes before having an ECG performed.
- The ECG will be performed before any procedures that may affect heart rate (HR), such as blood draws.

The Investigator will assess the ECG recording as 'normal', 'abnormal - not clinically significant', or 'abnormal - clinically significant' and include a description of the abnormality as required. The ECG parameters assessed will include HR, PR, QRS, QT and QTcF (calculated using Fredericia's method). ECGs at the CHDR will be obtained as electronic course, using Marquette 800/2000/5500 or Dash 3000 and stored using the MUSE Cardiology Information System. ECGs at the LUMC will be obtained using Mortara ELI 380, which the paper source will be the printout of the ECG.

7.2.5 Columbia Suicide Severity Rating Scale (C-SSRS)

The C-SSRS is a clinical rating of suicidal behaviour and ideation. The C-SSRS consists of a maximum of 20 items. The Baseline/Screening version of the C-SSRS will be performed at screening (Day -31 to admission). The Since Last Visit version of the C-SSRS will be performed at Day -1 of Period 1 and 2 (Part A) or at admission before Period 1 (Part B). Reports of suicidal ideation with intent to act (endorse item 4 or 5) and reports of actual, aborted, or interrupted suicide attempts or a behaviour preparatory for making an attempt indicate subjects at high risk for suicide. If such reports are identified, the Investigator is to appropriately manage the subject in accordance with standard of care.

An example of the C-SSRS is presented in Appendix B and Appendix C.

7.2.6 Continuous monitoring

On days of study drug administration (see Table 1 - 4) continuous monitoring of the vital signs will be performed. Vital signs will include the blood pressure (systolic and diastolic blood pressure, mean arterial pressure [MAP]), HR, respiratory frequency and SpO_2. Continuous ECG monitoring will be performed during the baseline ventilation period and the dose administration period of the buprenorphine/placebo.

Recording of the vital signs will start before the baseline ventilation period and will continue after transfer to the PACU. The following measure will be recorded as an AE:

- Heart rate < 30 bpm;
- Systolic blood pressure > 160 mmHg;
- Diastolic blood pressure > 110 mmHg;
- MAP < 55 mmHg;
- Arrhythmias;
- Ventricular extra systoles > 6/minute or triplets;
- Apnoea > 60 seconds.

7.2.7 Laboratory assessments

Laboratory parameters

Blood and urine samples will be analysed at a local laboratory. At the screening visit and EOS/ET visit, blood specimens will be collected for safety laboratory tests following at least a 4-hour fast.

Blood and urine samples for clinical laboratory assessments will be collected as shown in Table 1 - 4. Laboratory test results that are abnormal and considered clinically significant will be reported as AEs (see Section 8.1).

The safety laboratory test panels are shown in Table 7. Unscheduled safety blood sampling may include arterial blood gas collection if deemed necessary. Total blood volumes to be collected are in Section 7.5.

Table 7: Safety laboratory test panels

Lab	Tests	Collection & Analysis
Haematology	Haemoglobin [including mean corpuscular volume (MCV), mean corpuscular haemoglobin (MCH), mean corpuscular haemoglobin concentration (MCHC)], haematocrit, red cell count (RBC), total white cell count (WBC) and platelet count. Differential blood count, including: basophils, eosinophils, neutrophils, lymphocytes and monocytes.	2 mL of venous blood in a BD Vacutainer® K_2EDTA tube. Samples will be analysed by the Clinical Chemistry Laboratory (AKCL) of the LUMC
Chemistry and electrolytes	Sodium, potassium, chloride, bicarbonate, calcium, total protein, albumin, cholesterol, triglycerides, blood urea nitrogen, creatinine, total bilirubin[1], alkaline phosphatase, AST, ALT, amylase, lipase and TSH	3.5 mL of venous blood in a BD Vacutainer® SST Gel and Clot Activator tube. Samples will be analysed by the AKCL of the LUMC
Glucose	Glucose[2]	2 mL of venous blood in a BD Vacutainer® Sodium Fluoride tube. Samples will be analysed by the AKCL of the LUMC
HbA1c	HbA1c	4 mL venous blood in an EDTA tube. Samples will be analysed by the AKCL of the LUMC
Coagulation	PT and INR	2.7 mL venous blood in a citrate (9NC) tube. Samples will be analysed by the AKCL of the LUMC
Serology	HIV1 and HIV2 antigen and antibodies, hepatitis B surface antigen, hepatitis B antibodies and hepatitis C antibodies	5 mL of venous blood in a BD Vacutainer® SST Gel and Clot Activator tube. Samples will be analysed by the Microbiology Laboratory (CKML) of the LUMC
Alcohol	Alcohol Breath Test	The hand-held Alco-Sensor IV meter (Honac, Apeldoorn, The Netherlands) will be used to measure the breath ethanol concentrations.
Urinalysis	Leukocytes, blood, nitrite, protein, urobilinogen, bilirubin, pH, specific gravity, ketones, glucose. If there is a clinically significant positive result, urine will be sent to the AKCL for microscopy and/or culture.	A midstream, clean-catch urine specimen will be analysed by dipstick (Multistix® 10 SG, Siemens Healthcare Diagnostics, Frimley, UK).
Pregnancy[3]	hCG. If there is a clinically significant, positive result, urine will be sent to the AKCL for confirmation.	A urine specimen will be analysed at CHDR by test kit

Lab	Tests	Collection & Analysis
		(InstAlert, Innovacon, San Diego, USA).
Urine drug screen	Cocaine, amphetamines, MDMA, methamphetamines, benzodiazepines and cannabinoids.<br>Part A only: opiates (morphine)	A urine specimen will be analysed at CHDR by test kit (InstAlert, Innovacon, San Diego, USA).

EDTA=Ethylenediaminetetraacetic acid; MDMA=3,4-methylenedioxymethamphetamine; SST=Serum Separator Tube.

[1]Conjugated bilirubin will be reported only when total bilirubin is outside the reference range. [2]After 4-hours fasting. [3] Serum pregnancy test for women of childbearing potential will be performed at screening and urine pregnancy test will be performed at upon admission to CHDR for each period and if pregnancy is suspected during the study.

7.3 Pharmacokinetic assessments

For the evaluation of plasma concentrations of buprenorphine and fentanyl, blood samples will be collected from all subjects and OT patients. Blood samples will be obtained from an arterial line placed in the left or right radial artery (opposite to the arm through which the drug is infused). If a fentanyl bolus is not administered, subsequent fentanyl PK samples will not be collected.

Indivior has previously collected PK data for OT patients using venous plasma collection. In order to use PK data from this study with previously developed PK/PD models, additional venous PK samples for buprenorphine will be collected in Part B only. These additional samples are not required in Part A, as buprenorphine doses used are lower for healthy subjects.

In Part A, blood samples will be collected from healthy subjects for arterial buprenorphine (6 mL) in all 3 periods and fentanyl (2 mL) in Periods 1 and 2 only, in order to maintain the blind. In Part B, blood samples will be collected from OT patients for fentanyl only in Period 1 and for buprenorphine (arterial and venous) and fentanyl in Period 2. Blood samples will be obtained at time points specified in Table 8.

Table 8: Buprenorphine and fentanyl PK sampling time points

	**PK sampling time points (minutes after start dose administration)**
**Buprenorphine Arterial** (17 samples)	t = 0 (pre-dose), 5, 10, 15, 20, 30, 60, 90, 120 180, 240, 300, 360, 375, 420, 480 and 540
**Fentanyl Arterial** (33 samples)	t = 120 (pre-bolus), 122, 125, 130, 135, 140, 150, 180 (pre-bolus), 182, 185, 190, 195, 200, 210, 240 (pre-bolus), 242, 245, 250, 255, 260, 270, 300 (pre-bolus), 302, 305, 310, 315, 320, 330, 360, 375, 420, 480 and 540<br>If a fentanyl bolus is not administered, subsequent fentanyl PK samples will not be collected.
**PART B ONLY: Buprenorphine Venous** (5 samples)	t = 120, 180, 240, 300, 360

Actual sampling times may change upon agreement of the clinical pharmacologist and Investigator, but the number of samples will remain the same. All efforts will be made to obtain the PK samples at the exact nominal time relative to dosing. Acceptable windows for sampling times are described in Section 7.4. The exact time of the sample collection will be noted.

Refer to the laboratory manual for sample collection and preparation procedure.

7.3.1 Labelling

Pre-printed, waterproof labels will be used to identify the tubes used during sample collection and for storage of separated plasma. Each label will contain the following information:

- CHDR Protocol number
- Subject Number
- Period number (date)
- Protocol (delta) time
- Activity: Sample type (blood) & purpose (chemistry)

7.3.2 Shipping Procedures

CHDR will arrange shipment of the samples. Samples must be shipped to Keystone Bioanalytical at time intervals agreed with the sponsor. Details can be found in the Lab Manual.

7.4 Sequence of assessments and time windows

When the following assessments are scheduled to be performed at the same time-point, the order will be as follows: ECG, vital signs, blood sampling for safety, physical examination. When a PK assessment is scheduled for the same nominal time as another scheduled assessment, the PK sample will take precedence.

The deviations of actual time points from the expected time points will be within 10%, calculated from the zero point (time of start drug administration) or the last relevant activity. Deviations of more than 10% will be explained in a note. Pre-dose assessments are given in indicative expected times.

7.5 Total blood volume

Total blood volume: Part A with 2 periods

Sample	Samples Taken		Sample Volume*		Volume	
Haematology	4	x	2	mL	8	mL
Chemistry	4	x	3.5	mL	14	mL
HbAlc	1	x	4	mL	4	mL
Glucose	4	x	2	mL	8	mL
Coagulation	1	x	2.7	mL	2.7	mL
Serology	1	x	5	mL	5	mL
PK buprenorphine	34	x	6	mL	204	mL
PK fentanyl	66	x	2	mL	132	mL
	**TOTAL blood volume /Subject**				**377.7**	**mL**

* exclusive discarded volume

Total blood volume: Part A with 3 periods

Sample	Samples Taken		Sample Volume*		Volume	
Haematology	5	x	2	mL	10	mL
Chemistry	5	x	3.5	mL	17.5	mL
HbAlc	1	x	4	mL	4	mL
Glucose	5	x	2	mL	10	mL
Coagulation	1	x	2.7	mL	2.7	mL
Serology	1	x	5	mL	5	mL
PK buprenorphine	51	x	6	mL	306	mL
PK fentanyl	66	x	2	mL	132	mL
	**TOTAL blood volume /Subject**				**487.2**	**mL**

* exclusive discarded volume

Total blood volume: Part B

Sample	Samples Taken		Sample Volume*		Volume	
Haematology	3	x	2	mL	6	mL
Chemistry	3	x	3.5	mL	10.5	mL
HbAlc	1	x	4	mL	4	mL
Glucose	3	x	2	mL	6	mL
Coagulation	1	x	2.7	mL	2.7	mL
Serology	1	x	5	mL	5	mL
PK buprenorphine (venous)	5	x	6	mL	30	mL
PK buprenorphine (arterial)	17	x	6	mL	102	mL
PK fentanyl	66	x	2	mL	132	mL
	**TOTAL blood volume /Subject**				**298.2**	**mL**

* exclusive discarded volume

8 SAFETY REPORTING

8.1 Definitions of adverse events

An AE is any untoward medical occurrence in a subject associated with the use of an investigational medicinal product (IMP) regardless of the presence of a causal relationship to the IMP. An AE can therefore be any unfavourable and unintended sign (including an abnormal laboratory or vital sign finding), symptom, or disease (new or exacerbated) temporally associated with an IMP, whether or not it is related to the IMP. Adverse events will be recorded from the time of the first screening visit through the end of the EOS/ET visit.

A procedure-related AE is defined by loss of respiratory activity for 60 seconds or longer, despite active stimulation of the subject, end-tidal partial pressure of carbon dioxide greater than 67.5 mmHg, O_2 saturation less than 85% for at least 2 minutes, or any other situation or condition that may interfere with the health of the participant. If Investigator(s) stimulate a subject to breathe or give supplemental oxygen as needed to prevent an AE, the subject will not proceed to the next fentanyl dose and the period will terminate early.

8.1.1 Intensity of adverse events

The intensity of clinical AEs is graded using a three-point scale as defined below:

- Mild: transient or mild discomfort; no limitation of usual activities; no medical intervention required;
- Moderate: mild to moderate limitation in activity; some limitation of usual activities; no or minimal medical intervention or therapy is required;
- Severe: marked limitation in activity; some assistance is usually required; medical intervention or therapy is required; hospitalisation is probable.

8.1.2 Relationship to study drug

For each AE, the relationship to drug must be provided as judged by the Investigator:

- Related - the cause of the AE is related to the IMP and cannot be reasonably explained by other factors (e.g., the subject's clinical state, concomitant therapy, and/or other interventions).
- Not related - data are available to identify a clear alternative cause for the AE other than the IMP.

8.1.3 Action

Eventual actions taken will be recorded.

8.1.4 Serious adverse events

The Investigator or designee is responsible for identifying, documenting and reporting events that meet the definition of an SAE.

An SAE is any event that meets any of the following criteria:

- Death
- Life-threatening
- Inpatient hospitalisation or prolongation of existing hospitalisation
- Persistent or significant disability/incapacity
- Congenital anomaly/birth defect in the offspring of a subject who received buprenorphine
- Other: Important medical events that may not result in death, be life-threatening or require hospitalisation, may be considered an SAE when, based upon appropriate medical judgment, they

may jeopardise the subject and may require medical or surgical intervention to prevent one of the outcomes listed in this definition. Examples of such events are:

- Intensive treatment in an emergency room or at home for allergic bronchospasm
- Blood dyscrasias or convulsions that do not result in inpatient hospitalisation
- Apnoea will be considered an important medical event if intervention(s) beyond usual study procedures/interventions (e.g. administration of naloxone, intensive care measures) are required to manage respiratory depression

- Potential Hy's Law cases indicative of medication-induced hepatocellular injury, defined as:
  - ALT ≥ 3x upper limit of normal (ULN) and total bilirubin of ≥ 2x ULN (or INR > 1.5 if measured)

  Note: INR threshold does not apply to subjects receiving anticoagulants

  - ALT or AST > 3x ULN with systemic symptoms (fatigue, nausea, vomiting, right upper quadrant pain or tenderness, fever, rash and/or eosinophilia [> 5%])

An AE is considered "life threatening" if the subject was at immediate risk of death from the event as it occurred; i.e., it does not include a reaction that if it had occurred in a more serious form might have caused death. For example, IMP-induced hepatitis that resolved without evidence of hepatic failure would not be considered life threatening even though IMP-induced hepatitis can be fatal.

Adverse events requiring prolongation of existing hospitalisation should be considered SAEs. Hospitalisation for elective surgery or routine clinical procedures that are not the result of AE (e.g., elective surgery for a pre-existing condition that has not worsened) should not be considered AEs or SAEs. If anything untoward is reported during the procedure, that occurrence must be reported as an AE (either 'serious' or 'non-serious') according to the usual criteria.

In general, hospitalisation signifies that the subject has been detained (usually involving at least an overnight stay) at the hospital or emergency ward for observation and/or treatment that would not have been appropriate in the physician's office or other outpatient setting. Complications that occur during hospitalisation are AEs. If a complication prolongs hospitalisation or fulfils any other serious criteria, the event is serious. When in doubt as to whether 'hospitalisation' occurred or was necessary, the AE should be considered serious.

8.1.5 Suspected unexpected serious adverse reactions

A suspected unexpected serious adverse reaction (SUSAR) is an SAE related to the IMP administered in any dose and that, in its nature or severity, is not consistent with the applicable product information (e.g., Investigator's Brochure for an unauthorised investigational product or summary of product characteristics for a marketed product).

8.1.6 Reporting of serious adverse events

SAEs and SUSARs will be reported according to the following procedure:

Once the Investigator (or designee) determines that an event meets the protocol definition of an SAE, the Investigator (or designee) will notify Indivior **within 24 hours** of becoming aware of the event by completing the appropriate eCRF(s). Any follow-up information on a previously reported SAE will also be reported to Indivior within 24 hours by updating the appropriate eCRF.

Where additional information is needed or expected, the Investigator will not wait to receive all information before reporting the event to Indivior. The Investigator must provide an assessment of causality at the time of the initial report as described in Section 8.1.2 of the protocol.

In the event that the eCRF is not available, a paper SAE Reporting Form should be completed and submitted to Indivior Pharmacovigilance (PV) via email or fax:

[redacted]

When the system is again available, the information recorded on the paper SAE Reporting Form should be entered into the eCRF.

Prompt receipt of notifications of SAEs from Investigators to Indivior or designated representative is essential in ensuring that legal obligations and ethical responsibilities regarding the safety of subjects are met.

Indivior PV will determine if an SAE meets the definition of a SUSAR. Indivior or designated representative will comply with country-specific regulatory requirements pertaining to safety reporting to competent authorities (CA)s, ethic committees (EC)s and investigators. Please refer to the safety management plan for more details.

8.1.7 Follow-up of adverse events

All AEs will be followed until they have abated, returned to baseline status or until a stable situation has been reached. Depending on the event, follow up may require additional tests or medical procedures as indicated, and/or referral to the general physician or a medical specialist.

8.2 Temporary halt for reasons of subject safety

In accordance with Section 10, Subsection 4 of the Medical Research Involving Human Subjects Act (WMO), the Investigator will inform the subjects and the EC if anything occurs, on the basis of which it appears that the disadvantages of participation may be significantly greater than were foreseen in the research proposal. The study will be suspended pending further review by the EC, except insofar as suspension would jeopardise the subjects' health. The Investigator will ensure that all subjects are kept informed.

8.3 Pregnancy

If a subject becomes pregnant when on study drug, the subject will be discontinued from the study and complete EOS procedures. If the female partner of a male subject becomes pregnant while participating in the study, permanent discontinuations of study drug should be considered. The Investigator must counsel the subject and discuss the risks of continuing with the pregnancy and the possible effects on the foetus. Monitoring of the subject should continue until the outcome of the pregnancy is known.

8.3.1 Reporting of pregnancy

Subjects will be counselled to inform the Investigator of any pregnancy that occurs during the treatment phase and for 90 days after the last dose of study drug. Pregnancy of a study subject or the partner of a study subject without associated unexpected or adverse sequelae is not a reportable AE, but it must be reported to Indivior PV using the Clinical Trial Pregnancy Reporting Form within 24 hours of the Investigator or designee first being aware of the pregnancy. Any pregnancy complication or elective termination for medical reasons must be reported as an AE.

The pregnancy must be followed up to determine outcome (including premature termination) and status of mother and infant. Any termination of pregnancy will be reported, regardless of foetal status (presence or absence of anomalies) or indication for procedure.

9 STATISTICAL METHODOLOGY AND ANALYSES

Statistical analysis will be performed separately for Part A (healthy subjects) and Part B (OT patients).

Continuous variables will be summarised using descriptive statistics such as means, standard deviations (SD), medians, minimums and maximums. Categorical variables will be reported as frequency counts (including number missing) and the percentage of subjects in corresponding categories. Individual subject data will be presented by subject in data listings. Data listings will include all data collected from the initial screening visit to the end of study for all subjects enrolled.

Baseline is defined as the last non-missing value prior to the buprenorphine or placebo infusion. The baseline for PD endpoints are defined differently in the corresponding section.

Demographic and baseline characteristic data will be summarised by treatment sequence; safety and PD data will be summarized by the treatments received, including placebo + fentanyl, buprenorphine + fentanyl and buprenorphine only for Part A and placebo + fentanyl and buprenorphine + fentanyl for Part B.

9.1 Statistical analysis plan

A Statistical Analysis Plan (SAP) will be written and finalised before the database lock. The SAP will provide full details of the statistical analyses, the data displays and the algorithms to be used for data derivations.

All safety and statistical programming will be conducted with SAS 9.4 for Windows or newer (SAS Institute Inc., Cary, NC, USA). Pharmacokinetic variable programming will be conducted as outlined in the SAP.

A separate Pharmacometric Assessment Plan (PAP) will be written to describe modelling related to PK/PD relationships. It will be written and finalised before the database lock.

9.2 Power calculation

Not applicable.

9.3 Missing, unused and spurious data

All missing or incomplete safety and PD data, including dates and times, are treated as such, and will not be imputed.

For graphical and summary purposes, PD and safety values below the limit of quantification will be set to half (½) of the limit of quantification. For analysis purposes, no undetermined values will be replaced.

If single data points for plasma drug concentrations are missing, the AUC parameters will be derived by interpolating with regard to the 2 neighbouring non-missing concentrations.

For calculation of PK parameters, all plasma drug concentration values below the quantification limit (BLQ) occurring prior to C_{max} will be replaced by 0, except for embedded BLQ values (between 2 measurable time points), which will be treated as “missing”. All BLQ values after C_{max} will be treated as “missing”.

The handling of missing, unused and spurious data will be documented in the study report.

9.4 Analysis sets

Data of all subjects participating in the study will be included in the analyses if the data can meaningfully contribute to the objectives of the study.

9.4.1 Safety set

The safety population will be defined as all subjects who received at least 1 dose of buprenorphine or placebo.

9.4.2 Pharmacokinetic analysis set
The PK analysis population is defined as all subjects who received at least 1 dose of buprenorphine or fentanyl and have at least one measurable drug concentration in samples collected.

9.4.3 Pharmacodynamic analysis set
The analysis population for pharmacodynamics is defined as all subjects who received at least 1 dose of buprenorphine or fentanyl and have at least one post-baseline assessment of the parameter being analysed.

9.5 Subject disposition
Subject disposition will be listed by subject.

The following number/percentage of subjects will be summarised:

- subjects screened
- subjects enrolled
- subjects completed
- subjects discontinued by period along with the reasons for study discontinuation
- subjects included in safety population
- subjects included in the PK/PD analysis population

A subject who completed the study is defined as a subject who completed the last visit assessments at Period 2 and the EOS assessments, if the subject didn't participate in Period 3, or who completed the last visit assessments at Period 3 and the EOS assessments, if the subject participated in Period 3.

9.6 Baseline parameters and concomitant medications

9.6.1 Demographics and baseline variables
Demographic and baseline characteristics (e.g., sex, race, age, weight, height,) will be summarised using descriptive statistics. Additionally, for Part B (OT patients), disease characteristics (e.g., history/severity of drug use, drug use at screening, etc.) will be summarised.

The results of the sedation VAS questionnaire at screening will only be listed.

9.6.2 Medical history
A detailed medical history will be obtained during the Screening period. This will include information regarding the subject's complete history of relevant medical conditions, diagnoses, procedures, treatments and any other noteworthy medical information. Any updates to medical history information made available during the course of the study will also be captured.

9.6.3 Concomitant Medications
Previous and concomitant medications will be coded by using the World Health Organisation Drug Dictionary (WHODD).

9.6.4 Treatment compliance/exposure
Exposure to buprenorphine/placebo will be described in terms of duration of treatment, and the average infusion dosage (mg/70 kg/h) for buprenorphine will be summarised.

The numbers of subjects exposed to fentanyl bolus will be summarised by received fentanyl dose levels (mg/70 kg) within the placebo infusion and buprenorphine infusion periods separately, and by the combination of received fentanyl and buprenorphine dose levels (when applicable) within the buprenorphine infusion period.

9.7 Safety and tolerability endpoints

The safety set will be used to perform all safety analyses. Safety results will be summarised separately for Part A (healthy subjects) and Part B (OT patients).

Change from baseline will be calculated for all continuous safety parameters.

9.7.1 Adverse events

The AE coding dictionary for this study will be Medical Dictionary for Regulatory Activities (MedDRA). The investigators determine the intensity of AEs and the relationship of AEs to study therapy. The AE terms are coded and grouped by system organ class (SOC) using the latest version of the Medical Dictionary for Regulatory Activities (MedDRA).

Treatment-emergent AE (TEAE) is defined as an AE observed after starting administration of the buprenorphine/placebo. If subject experiences an event both prior to starting administration of buprenorphine/placebo and ongoing during the treatment, the event will be considered a TEAE (of the treatment) only if it has worsened in severity (i.e., it is reported with the date of worsening as the new start date/time) after starting administration of the buprenorphine/placebo.

All TEAEs collected during the investigational period will be presented by SOC and preferred term, each in descending order of frequency, unless otherwise specified. Treatment-emergent AEs, treatment-related TEAEs and serious TEAEs will be summarised by intensity, SOC and preferred term, by treatment (buprenorphine/placebo) and received buprenorphine dose levels.

All AEs and TEAEs leading to study discontinuation will be displayed in listings.

9.7.2 Vital signs

At each time point, absolute values and change from baseline of supine blood pressure and pulse rate will be summarised by treatment (buprenorphine/placebo) and received buprenorphine dose levels, with n, mean, SD, standard error (SE), median, Min and Max values.

9.7.3 ECG

At each time point, absolute values and change from baseline of ECG numeric variables will be summarised by treatment (buprenorphine/placebo) and received buprenorphine dose levels, with n, mean, SD, SE, median, Min and Max values.

9.7.4 Clinical laboratory tests

At each time point, absolute values and change from baseline of clinical laboratory variables will be summarised with n, mean, SD, SE, median, Min and Max values. All laboratory data (including re-check values if present) will be listed chronologically.

9.8 Pharmacokinetic and pharmacodynamic endpoints

9.8.1 Pharmacokinetics

Individual plasma drug concentrations will be plotted versus actual sampling time per individual using both a linear and log y-axis. Additionally, concentration versus time curves will be plotted per treatment group as a spaghetti plot with the group median added. Individual subject listings will also be provided. Concentrations will be summarised by received buprenorphine and fentanyl dose levels, with for each time point of measurement the number of observations, mean, SD, median, Min and Max.

Individual PK parameters will be determined based on the concentration versus actual sampling time curves: The PK parameters will be statistically summarised by received buprenorphine and fentanyl dose levels.

9.8.2 Pharmacodynamics

The primary endpoint is the peak ventilatory depression (absolute values and percentage changes). For buprenorphine or placebo, the peak ventilatory depression from baseline value will be summarised by placebo and received buprenorphine dose levels. For fentanyl, the peak ventilatory depression from the pre-fentanyl baseline immediately before the first fentanyl bolus will be summarised by received fentanyl dose levels.

The baseline value is the minute ventilation value when a stable ventilation pattern is established for at least 2 minutes prior to the infusion of buprenorphine/placebo. The pre-fentanyl baseline value is defined as the minute ventilation value averaged between the timepoint when a stable ventilation pattern is established for at least 2 minutes after start of buprenorphine/placebo infusion and the timepoint before the first fentanyl bolus.

Part A (Healthy subjects) and Part B (OT patients) have different secondary endpoints.

For Part A, the number (percentage) of subjects who experience apnoea and the number (percentage) of subjects who require stimulation for breathing will be summarised for individual fentanyl dose levels by placebo versus buprenorphine infusion period and by received buprenorphine dose levels.

For Part B, the number (percentage) of subjects who experience apnoea during buprenorphine treatment at the fentanyl dose, at which the subject had apnoea during the placebo treatment will be summarised for the OT patients

The PD parameters will be listed by treatment (buprenorphine/placebo) and received buprenorphine and fentanyl dose levels, subject, visit and time. Individual graphs by time will be generated, with both treatments (buprenorphine/placebo) in one graph.

All repeatedly measured PD endpoints will be summarised (n, mean, SD, standard error of the mean (SEM), median, Min and Max values) by treatment (placebo/buprenorphine) and received buprenorphine and fentanyl dose levels and time, and will also be presented graphically as mean over time, with standard deviation as error bars. All single measured PD endpoints will be summarised (mean, SD, SEM, median, Min and Max values) by treatment (buprenorphine/placebo) and received buprenorphine dose levels and will also be presented graphically as mean in a bar graph, with standard deviation as error bars.

Further exploratory statistical analysis may be attempted with statistical testing, e.g. Fisher's exact test on number of subjects with apnoea.

9.8.3 Inferential methods

The study is exploratory, no statistical hypothesis tests will be performed, and there will be no multiplicity adjustments.

9.8.4 Pharmacokinetic/Pharmacodynamic modelling

In short, in the first stage, compartmental models will be fitted to the buprenorphine and fentanyl concentration data. In the second stage, the empirical Bayesian estimated concentration profiles serve as input of a model consisting of a hypothetical effect site where buprenorphine and fentanyl compete for binding to receptors. The relationship between drug binding and ventilatory depression will be related to the association/disassociation and efficacy parameters of the 2 drugs. Finally, the validity of the models will be assessed with various goodness-of-fit diagnostics.

The buprenorphine – fentanyl PD interaction model will be based on the one described by Olofsen et al. [16]:

$$d[BR]/dt = k_{on,B}.\ [B].\ [R] - k_{off,B}.\ [BR]$$

$$d[FR]/dt = k_{on,F}.\ [F].\ [R] - k_{off,F}.\ [FR]$$

where B, F and R denote buprenorphine, fentanyl and receptors, respectively; [] denotes effect-site concentration; and k_{on} and k_{off} denote association and disassociation rate constants, respectively. Because $k_{off,F}$ is large [13], the concentration of receptors bound to fentanyl can be written as:

$$[FR] = (1 - [BR]).\ ([F]/C_{50,F}]^{\gamma}) / (1 + ([F]/C_{50,F}]^{\gamma})),$$

where $C_{50,F} = k_{off,F} / k_{on,F}$, and γ an additional shape parameter. The relationship between the bound receptor concentrations and ventilation will be based on:

$$V = V_0.\ (1 - \alpha_B.\ [BR] - \alpha_F.\ [FR]),$$

where α_B and α_F denote potency parameters. This equation holds for the separate effects of buprenorphine and fentanyl as described by Yassen et al. [13], holds for an agonist and an antagonist (with one of the αs = 0), agonists (with the αs close to 1), and may be at least a good starting point for an agonist and a partial agonist, with one α close to 1 and one α significantly lower than one. For the drugs administered separately, Yassen et al. found $\alpha_B = 0.56$, and $\alpha_F = 0.91$ [13].

The effect-site concentrations can be computed using compartmental models for drug distribution, including distribution to an effect site characterised by equilibration delay half-lives.

Model parameters (population medians and interindividual variability) will be estimated with NONMEM (software for non-linear mixed effects modelling; ICON plc, Gaithersburg, MD).

9.9 Exploratory analyses and deviations

Exploratory data-driven analyses can be performed with the caveat that any statistical inference will not have any confirmatory value.

Deviations from the original SAP or PAP will be documented in the clinical study report.

9.10 Interim analyses

No formal interim analysis will be performed.

10 GOOD CLINICAL PRACTICE, ETHICS AND ADMINISTRATIVE PROCEDURES

10.1 Good clinical practice

10.1.1 Ethics and good clinical practice

The Investigator will ensure that this study is conducted in full compliance with the protocol, the principles of the Declaration of Helsinki [28], International Conference on Harmonisation (ICH) Good Clinical Practise (GCP) guidelines [29], and with the laws and regulations of the country in which the clinical research is conducted.

10.1.2 Ethics committee

The Investigator will submit this protocol and any related documents to an EC and the CA. Approval from the EC and the statement of no objection from the CA must be obtained before starting the study, and should be documented in a dated letter/email to the Investigator, clearly identifying the trial, the documents reviewed and the date of approval. A list of EC members must be provided, including the functions of these members. If study staff were present, it must be clear that none of these persons voted.

Modifications made to the protocol after receipt of the EC approval must also be submitted as amendments by the Investigator to the EC in accordance with local procedures and regulations.

10.1.3 Informed consent

It is the responsibility of the Investigator to obtain written informed consent from each individual participating in this study after adequate explanation of the aims, methods, objectives and potential hazards of the study. The Investigator must also explain to the subjects that they are completely free to refuse to enter the study or to withdraw from it at any time for any reason.

The Informed Consent and Subject Information will be provided to the subject in Dutch.

10.1.4 Insurance

CHDR has a liability insurance which is in accordance with Article 7, Subsection 6 of the WMO.

CHDR has an insurance, which is in accordance with the legal requirements in The Netherlands (Article 7 WMO and the Measure regarding Compulsory Insurance for Clinical Research in Humans of 23 June 2003). This insurance provides cover for damage to research subjects through injury or death caused by the study.

- € 650,000.- (i.e., six hundred and fifty thousand Euro) for death or injury for each subject who participates in the Research;
- € 5,000,000.- (i.e., five million Euro) for death or injury for all subjects who participate in the Research;
- € 7,500,000.- (i.e., seven million and five hundred thousand Euro) for the total damage incurred by the organisation for all damage disclosed by scientific research for the sponsor as 'verrichter' in the meaning of said Act in each year of insurance coverage.

The insurance applies to the damage that becomes apparent during the study or within 4 years after the end of the study.

10.2 Study funding

Indivior UK Ltd is the sponsor of the study and is funding the study. All financial details are provided in the separate contract(s) between the CHDR and the sponsor.

10.3 Data handling and record keeping

10.3.1 Source data collection

The Investigator is responsible for the quality of the data recorded in the CRFs. The data recorded should be a complete and accurate account of the subject's record collected during the study. Source documents will be electronic or paper, as described below.

All source documents pertaining to this study will be maintained by the Investigator and made available for direct inspection by the authorised study personnel outlined in the ICF.

The following source data will be recorded on paper, and entered into the eCRF:

- Identification and ICF
- Pregnancy results and pregnancy forms
- Urine analysis
- UDS
- C-SSRS
- ECGs (from LUMC)
- Drug administration
- Sedation VAS

The following source data will be recorded in the electronic source, PromaSys, then entered into the eCRF:

- Medical history
- Demographics
- AEs and concomitant medication
- Vital signs
- Height/weight

The following source data will be recorded in the electronic source, Promasys: and will be electronically transferred to Electronic Data Capture (EDC):

- Drug use history
- Visit dates
- Demographics
- Physical examination
- Contraceptives
- Inclusion / Exclusion
- Alcohol breath test
- Safety blood chemistry, haematology, coagulation and serology
- Treatment allocation (part A only)
- PK draw times
- ECGs (From CHDR)

The ventilation source for breath-to-breath and 1-minute summaries will be recorded using RESREG/ACQ software and will be electronically transferred to EDC.

10.3.2 Database management

Electronic Data Capture via eCRFs will be used for this study. The EDC system will include electronic CRFs designed to capture study information and has an audit trail to log all subsequent changes to the data. The data will be subjected to data consistency and validation checks, and the discrepancies generated will be used to raise queries after reference to the source workbooks. Data queries requiring clarification will be generated and addressed by the clinical sites. Only authorized personnel will make corrections to the eCRFs, and all corrections will be documented in an audit trail. The data will be handled confidentially and if possible anonymously. After

the database has been declared complete and accurate, the EDC database will be locked. Any changes to the database after that time can only be made by joint written agreement between the Investigator, sponsor and the statistician.

The eCRFs (including queries and audit trails) will be retained by Indivior. An electronic copy of the eCRF in a PDF format will be sent to Indivior from CHDR to maintain for their records.

10.4 Access to source data and documents

All study data will be handled confidentially. The Investigator will retain the originals of all source documents generated at CHDR for a period of 2 years after the report of the study has been finalised, after which all study-related documents will be archived (at a minimum) on micro-film which will be kept according to GCP regulations. After 2 years the sponsor will be notified that the source documents can be retained with the sponsor or destroyed.

The Investigator will permit trial-related monitoring, audits, EC review and CA inspections, providing direct access to source data and documents.

10.5 Protocol deviations

A protocol deviation is any noncompliance with the clinical study protocol or ICH/GCP requirements. The noncompliance may be either on the part of the subject, the Investigator, or the study site staff and may be identified based on conditions related to the categories below:

- Protocol Inclusion/Exclusion criteria
- Forbidden concomitant medications
- Missing evaluations for relevant endpoints
- Other protocol deviations occurring during study conduct

It is the responsibility of the Investigator and study site staff to use continuous vigilance to identify and report deviations to Indivior. Important deviations require immediate notification to the sponsor. Important protocol deviations must be sent to the EC, as required. The Investigator and study site staff are responsible for knowing and adhering to the EC's requirements.

In the event of a deviation from the protocol due to an emergency or accident, the Investigator or designated individual must contact the sponsor at the earliest possible time by telephone. This will allow an early joint decision regarding the subject's continuation in the study. This decision will be documented by the Investigator and the sponsor, and reviewed by the monitor.

10.6 Quality control and quality assurance

This study will be conducted according to applicable SOPs. Quality assurance will be performed under the responsibility of CHDR's Quality Assurance manager.

10.6.1 Monitoring

An initiation visit will be performed before the first subject is included. Monitoring visits and contacts will occur at regular intervals thereafter, according to a frequency defined in the study-specific monitoring plan. A close-out visit will be performed after study closure.

10.7 Protocol amendments

Any change to a protocol has to be considered as an amendment.

10.7.1 Substantial amendment

Significant changes that affect subject safety and/or the scientific value of a trial require a substantial amendment. Examples of significant changes are given in European Union (EU) guidelines on the request to

the CA for authorisation of a clinical trial on a medicinal product for human use, the notification of substantial amendments and the declaration of the end of the trial (CT-1, 2010/C 82/01). The need for submitting a substantial amendment is the responsibility of the sponsor. Substantial amendments are to be approved by the appropriate EC and the CA will need to provide a 'no grounds for non-acceptance' notification prior to the implementation of the substantial amendment.

10.7.2 Non-substantial amendment

Non-substantial amendments do not affect subject safety or the scientific integrity of the trial. Non-substantial amendments will be approved (signed) by the Investigator(s) and will be recorded and filed by the Investigator/sponsor. Non-substantial amendments will be submitted to the EC for information only. The CA will only be notified by changes in EudraCT form and ABR form (if applicable) at toetsingonline. The implementation of a non-substantial amendment can be done immediately.

The EU guideline CT-1 2010/C 82/01 stipulates the importance of preventing over-reporting. Therefore, the following changes are by definition non-substantial in this study:

- change in amount and timing of the samples (maximum of 2 samples without a > 50 ml increase in the amount of blood taken and not exceed 500 ml of blood in total);
- changes in assay-type and/or institution where an assay will be performed, provided that validated assays will be used;
- editorial changes to documents in the submission dossier including the volunteer information sheets and the protocol. An editorial change is defined as a modification in the documents of typographical errors and other modifications that in no way alter the meaning or content of the document;
- determination of additional parameters in already collected materials, which are in agreement with the study objectives and do not provide prognostic or genetic information;
- other statistical analyses than described in the protocol;
- change in clinical staff, including the Principal Investigator, when this concerns regular staff members of CHDR who comply with internal regulations for training and authorisation; and
- change in dosing schedule in an ascending dose trial, provided the expected exposure of the subjects does not exceed the pre-set values indicated in this protocol.

10.7.3 Urgent amendment

An urgent amendment might become necessary to preserve the safety of the subjects included in the study. The requirements for approval should in no way prevent any immediate action being taken by the investigators or the sponsor in the best interests of the subjects. Therefore, if deemed necessary, an Investigator can implement an immediate change to the protocol for safety reasons. This means that, exceptionally, the implementation of urgent amendments will occur before submission to and approval by the EC(s) and CA.

10.8 End of study report

CHDR will notify the EC and the CA of the end of the study within a period of 90 days. The end of the study is defined as the last subject's last visit. In case the study is ended prematurely, CHDR will notify the EC and the CA within 15 days, including the reasons for the premature termination.

CHDR will notify the EC immediately of a temporary halt of the study, including the reason of such an action.

Within one year after the end of the study, the Investigator and/or sponsor will submit a final study report with the results of the study, including any publications/abstracts of the study, to the EC and the CA. The Principal Investigator and the sponsor's representative will be the signatories for the study report.

10.9 Public disclosure and publication policy

The results of the study will be published. The list of authors of any formal publication or presentation of study results may include, as appropriate, representatives of CHDR, LUMC or the sponsor and will be determined by mutual agreement.

11 REFERENCES

1. National Survey On Drug Use And Health. Key Substance Use and Mental Health Indicators in the United States: Results from the 2016 National Survey on Drug Use and Report, https://www.samhsa.gov/sites/default/files/topics/data_outcomes_quality/nsduh-ppt-09-2017.pdf, September 2017; accessed 18 May 2018.
2. National Institutes of Health, Drug Facts: Understanding Drug Use and Addiction. https://d14rmgtrwzf5a.cloudfront.net/sites/default/files/df_understanding_drug_use_final_08_2016.pdf, August 2016; accessed 18 May 2018.
3. Facing Addiction in America: The Surgeon General's Report on Alcohol, Drugs, and Health. Substance Abuse and Mental Health Services Administration (US); Office of the Surgeon General (US). Washington (DC): US Department of Health and Human Services; 2016 Nov. https://addiction.surgeongeneral.gov/sites/default/files/surgeon-generals-report.pdf; accessed 18 May 2018.
4. United Nations Office on Drugs and Crime. World Drug Report 2017, May 2017; ISBN: 978-92-1-148291-1, eISBN: 978-92-1-060623-3;
5. National Institute on Drug Abuse, https://www.drugabuse.gov/related-topics/trends-statistics/overdose-death-rates, September 2017; accessed 18 May 2018.
6. Rudd R, Seth P, David F, Scholl L. Increases in Drug and Opioid-Involved Overdose Deaths — United States, 2010–2015. MMWR Morb Mortal Wkly Rep 2016; 65:1445-52.
7. van Amsterdam J, Phillips L, Henderson G, Bell J, Bowden-Jones O, Hammersley R, Ramsey J, Taylor P, Dale-Perera A, Melichar J, van den Brink W, Nutt D. Ranking the harm of non-medically used prescription opioids in the UK. Regul Toxicol Pharmacol. 2015; 73:999-1004;
8. Bird SM, McAuley A, Perry S, Hunter C. Effectiveness of Scotland's National Naloxone Programme for reducing opioid-related deaths: a before (2006-10) versus after (2011-13) comparison. Addiction. 2016; 111:883-89;1
9. Middleton J, McGrail S, Stringer K. Drug related deaths in England and Wales. BMJ. 2016; 355:i5259;
10. Schwartz RP, Gryczynski J, O'Grady KE, Sharfstein JM, Warren G, Olsen Y, Mitchell SG, Jaffe JH. Opioid agonist treatments and heroin overdose deaths in Baltimore, Maryland, 1995-2009. Am J Public Health. 2013; 103:917-922;
11. Sinicina I, Sachs H, Keil W. Post-mortem review of fentanyl-related overdose deaths among identified drug users in Southern Bavaria, Germany, 2005-2014. Drug Alcohol Depend. 2017; 180:286-291;
12. Dahan A, Yassen A, Bijl H, Romberg R, Sarton E, Teppema L, Olofsen E, Danhof M. Comparison of the respiratory effects of intravenous buprenorphine and fentanyl in humans and rats. Br J Anaesth. 2005; 94:825-834;
13. Yassen A, Olofsen E, Romberg R, Sarton E, Teppema L, Danhof M, Dahan A. Mechanism-based PK/PD modeling of the respiratory depressant effect of buprenorphine and fentanyl in healthy volunteers. Clin Pharmacol Ther. 2007; 81:50-58;
14. Greenwald MK, Johanson CE, Moody DE, Woods JH, Kilbourn MR, Koeppe RA, Schuster CR, Zubieta JK. Effects of buprenorphine maintenance dose on mu-opioid receptor availability, plasma concentrations, and antagonist blockade in heroin-dependent volunteers. Neuropsychopharmacology. 2003; 28:2000-2009;
15. Greenwald MK, Comer SD, Fiellin DA. Buprenorphine maintenance and mu-opioid receptor availability in the treatment of opioid use disorder: implications for clinical use and policy. Drug Alcohol Depend. 2014; 144:1-11;
16. Olofsen E, van Dorp E, Teppema L, Aarts L, Smith TW, Dahan A, Sarton E. Naloxone reversal of morphine- and morphine-6-glucuronide-induced respiratory depression in healthy volunteers: a

mechanism-based pharmacokinetic-pharmacodynamic modeling study. Anesthesiology. 2010; 112:1417-27;

17. Boom M, Niesters M, Sarton E, Aarts L, Smith TW, Dahan A. Non-analgesic effects of opioids: opioid-induced respiratory depression. Curr Pharm Des. 2012; 18:5994-6004;
18. Pattinson KT. Opioids and the control of respiration. Br J Anaesth. 2008; 100:747-758;
19. van der Schrier R, Roozekrans M, Olofsen E, Aarts L, van Velzen M, de Jong M, Dahan A, Niesters M. Influence of Ethanol on Oxycodone-induced Respiratory Depression: A Dose-escalating Study in Young and Elderly Individuals. Anesthesiology. 2017; 126:534-542;
20. Yassen A, Olofsen E, van Dorp E, Sarton E, Teppema L, Danhof M, Dahan A. Mechanism-based pharmacokinetic-pharmacodynamic modelling of the reversal of buprenorphine-induced respiratory depression by naloxone: a study in healthy volunteers. Clin Pharmacokinet. 2007; 46:965-80;
21. Boom M, Olofsen E, Neukirchen M, Fussen R, Hay J, Groeneveld GJ, Aarts L, Sarton E, Dahan A. Fentanyl utility function: a risk-benefit composite of pain relief and breathing responses. Anesthesiology. 2013; 119:663-674;
22. Jensen ML, Foster DJ, Upton RN, Kristensen K, Hansen SH, Jensen NH, Nielsen BN, Skram U, Villesen HH, Christrup L. Population pharmacokinetics of buprenorphine following a two-stage intravenous infusion in healthy volunteers. Eur J Clin Pharmacol. 2007; 63:1153-1159;
23. Williams JT, Ingram SL, Henderson G, Chavkin C, von Zastrow M, Schulz S, Koch T, Evans CJ, Christie MJ. Regulation of μ-opioid receptors: desensitization, phosphorylation, internalization, and tolerance. Pharmacol Rev. 2013; 65:223-254;
24. Nasser AF, Greenwald MK, Vince B, Fudala PJ, Twumasi-Ankrah P, Liu Y, Jones JP 3rd, Heidbreder C. Sustained-Release Buprenorphine (RBP-6000) Blocks the Effects of Opioid Challenge With Hydromorphone in Subjects With Opioid Use Disorder. J Clin Psychopharmacol. 2016; 36:18-26;
25. Sittl R, Nuijten M, Nautrup BP. Changes in the prescribed daily doses of transdermal fentanyl and transdermal buprenorphine during treatment of patients with cancer and noncancer pain in Germany: results of a retrospective cohort study. Clin Ther. 2005; 27:1022-1031;
26. Sarton E, Teppema L, Dahan A. Naloxone reversal of opioid-induced respiratory depression with special emphasis on the partial agonist/antagonist buprenorphine. Adv Exp Med Biol. 2008; 605:486-491;
27. Dahan A, Yassen A, Romberg R, Sarton E, Teppema L, Olofsen E, Danhof M. Buprenorphine induces ceiling in respiratory depression but not in analgesia. Br J Anaesth. 2006; 96:627-632.
28. The Declaration of Helsinki, https://www.wma net/policies-post/wma-declaration-of-helsinki-ethical-principles-for-medical-research-involving-human-subjects/, October 2013; accessed 18 May 2018.
29. ICH Harmonised Triparitite Guideline, Guideline for Good Clinical Practice, http://www.ich.org/products/guidelines/efficacy/efficacy-single/article/integrated-addendum-good-clinical-practice.html, November 2016; accessed 18 May 2018.

APPENDIX A: MORPHINE EQUIVALENT DOSING FOR ORAL OPIOID DRUGS

Opioid Oral Morphine Milligram Equivalent (MME) Conversion Factors[1,2]

Type of Opioid (strength units)	MME Conversion Factor
Buprenorphine film/tablet[3] (mg)	30
Buprenorphine patch[4] (mcg/hr)	12.6
Buprenorphine film (mcg)	0.03
Butorphanol (mg)	7
Codeine (mg)	0.15
Dihydrocodeine (mg)	0.25
Fentanyl buccal or SL tablets, or lozenge/troche[5] (mcg)	0.13
Fentanyl film or oral spray[6] (mcg)	0.18
Fentanyl nasal spray[7] (mcg)	0.16
Fentanyl patch[8] (mcg)	7.2
Hydrocodone (mg)	1
Hydromorphone (mg)	4
Levorphanol tartrate (mg)	11
Meperidine hydrochloride (mg)	0.1
Methadone[9] (mg)	3
>0, <= 20	4
>20, <=40	8
>40, <=60	10
>60	12
Morphine (mg)	1
Opium (mg)	1
Oxycodone (mg)	1.5
Oxymorphone (mg)	3
Pentazocine (mg)	0.37
Tapentadol [10] (mg)	0.4
Tramadol (mg)	0.1

[1] The MME conversion factor is intended only for analytic purposes where prescription data is used to calculate daily MME. It is to be used in the formula: Strength per Unit X (Number of Units/ Days Supply) X MME conversion factor = MME/Day. This value does not constitute clinical guidance or recommendations for converting patients from one form of opioid analgesic to another. Please consult the manufacturer's full prescribing information for such guidance. Use of this file for the purposes of any clinical decision-making warrants caution.

[2] National Center for Injury Prevention and Control. CDC compilation of benzodiazepines, muscle relaxants, stimulants, zolpidem, and opioid analgesics with oral morphine milligram equivalent conversion factors, 2016 version. Atlanta, GA: Centers for Disease Control and Prevention; 2016. Available at https://www.cdc.gov/drugoverdose/media/. For more information, send an email to Mbohm@cdc.gov.

[3] Buprenorphine formulations with a FDA approved indication for Medication Assisted Treatment (MAT) are excluded from Medicare's Overutilization Monitoring System's opioid overutilization reporting.

[4]The MME conversion factor for buprenorphine patches is based on the assumption that one milligram of parenteral buprenorphine is equivalent to 75 milligrams of oral morphine and that one patch delivers the dispensed micrograms per hour over a 24 hour day. Example: 5 ug/hr buprenorphine patch X 24 hrs = 120 ug/day buprenorphine = 0.12 mg/day = 9 mg/day oral MME. In other words, the conversion factor not accounting for days of use would be 9/5 or 1.8.
However, since the buprenorphine patch remains in place for 7 days, we have multiplied the conversion factor by 7 (1.8 X 7 = 12.6). In this example, MME/day for four 5 μg/hr buprenorphine patches dispensed for use over 28 days would work out as follows: Example: 5 ug/hr buprenorphine patch X (4 patches/28 days) X 12.6 = 9 MME/day. Please note that because this allowance has been made based on the typical dosage of one buprenorphine patch per 7 days, you should first change all Days Supply in your prescription data to follow this standard, i.e., Days Supply for buprenorphine patches= # of patches x 7.
[5]The MME conversion factor for fentanyl buccal tablets, sublingual tablets, and lozenges/troche is 0.13. This conversion factor should be multiplied by the number of micrograms in a given tablet or lozenge/troche.
[6]The MME conversion factor for fentanyl film and oral spray is 0.18. This reflects a 40% greater bioavailability for films compared to lozenges/tablets and 38% greater bioavailability for oral sprays compared to lozenges/tablets.
[7]The MME conversion factor for fentanyl nasal spray is 0.16, which reflects a 20% greater bioavailability for sprays compared to lozenges/tablets.
[8] The MME conversion factor for fentanyl patches is based on the assumption that one milligram of parenteral fentanyl is equivalent to 100 milligrams of oral morphine and that one patch delivers the dispensed micrograms per hour over a 24 hour day. Example: 25 ug/hr fentanyl patch X 24 hrs = 600 ug/day fentanyl = 60 mg/day oral morphine milligram equivalent.
In other words, the conversion factor not accounting for days of use would be 60/25 or 2.4.
However, since the fentanyl patch remains in place for 3 days, we have multiplied the conversion factor by 3 (2.4 X 3 = 7.2). In this example, MME/day for ten 25 μg/hr fentanyl patches dispensed for use over 30 days would work out as follows:
Example: 25 ug/hr fentanyl patch X (10 patches/30 days) X 7.2 = 60 MME/day. Please note that because this allowance has been made based on the typical dosage of one fentanyl patch per 3 days, you should first change all Days Supply in your prescription data to follow this standard, i.e., Days Supply for fentanyl patches= # of patches X 3.
[9] The CDC MME conversion factor to calculate morphine milligram equivalents is 3. CMS uses this conversion factor when analyzing Medicare population opioid use. CMS uses the graduated methadone MME conversion factors to calculate MME within the Overutilization Monitoring System (OMS) for identifying and reporting potential opioid overutilizers. https://www.cdc.gov/drugoverdose/pdf/calculating_total_daily_dose-a.pdf.
[10] Tapentadol is a mu receptor agonist and norepinephrine reuptake inhibitor. Oral MMEs are based on degree of mu-receptor agonist activity, but it is unknown if this drug is associated with overdose in the same dose-dependent manner as observed with medications that are solely mu receptor agonists

APPENDIX B: COLUMBIA SUICIDE SEVERITY RATING SCALE (C-SSRS) - SCREENING/BASELINE

SUBJECT NUMBER:

VISIT: Screening DATE: d d m m m y y y y TIME: 24hour clock

SUÏCIDALE IDEATIE		
Stel vraag 1 en 2. Indien beide vragen negatief worden beantwoord: ga naar het onderdeel 'Suïcidaal Gedrag'. Indien vraag 2 met 'ja' wordt beantwoord, stel dan vraag 3, 4 en 5. Indien het antwoord op vraag 1 en/of vraag 2 'ja' is, vul het onderdeel 'intensiteit van ideatie' in.	**Leven: tijd dat hij/zij zich het meest suïcidaal voelde**	**Afgelopen __ maanden**
**1. Wens om dood te zijn**<br>Deelnemer koestert gedachten over de wens om dood te zijn, of niet langer meer te leven, of de wens om in slaap te vallen en niet meer wakker te worden.<br>***Hebt u gewenst dat u dood was of dat u kon gaan slapen en niet meer wakker zou worden?***<br>Zo ja, omschrijven:	**Ja** ☐ **Nee** ☐	**Ja** ☐ **Nee** ☐
**2. Niet-specifieke actieve suïcidale gedachten**<br>Algemene niet-specifieke gedachten over het willen beëindigen van je leven/zelfmoord te plegen (bijvoorbeeld: *'Ik heb aan zelfmoord gedacht'*) zonder gedachten aan manieren om zichzelf te doden/geassocieerde methoden, voornemen of plan tijdens de beoordelingsperiode.<br>***Hebt u daadwerkelijk wel eens gedacht aan zelfmoord?***<br>Zo ja, omschrijven:	**Ja** ☐ **Nee** ☐	**Ja** ☐ **Nee** ☐
**3. Actieve suïcidale ideatie met ongeacht welke methode (zonder plan) zonder intentie om te handelen**<br>De deelnemer koestert gedachten over suïcide en heeft tijdens de beoordelingsperiode aan ten minste één methode gedacht. Dit is anders dan een specifiek plan waarin de details voor wat betreft de tijd, plaats of methode zijn uitgewerkt (bijvoorbeeld de gedachte aan een methode voor zelfmoord, maar geen specifiek plan). Hieronder valt iemand die zou zeggen, *'Ik heb er over gedacht om een overdosis te nemen, maar nooit een specifiek plan gemaakt waar, wanneer of hoe ik dit eigenlijk zou doen ... en ik zou het nooit doorzetten'*<br>***Hebt u nagedacht over hoe u dit misschien zou doen?***<br>Zo ja, omschrijven:	**Ja** ☐ **Nee** ☐	**Ja** ☐ **Nee** ☐
**4. Actieve suïcidale ideatie met enige intentie om te handelen, zonder specifiek plan**<br>Actieve suïcidale gedachten over zelfmoord en deelnemer geeft aan enige intentie te hebben naar zulke gedachten te handelen, in tegenstelling tot *'Ik heb die gedachten maar zou er zeker nooit iets mee doen'*.<br>***Hebt u deze gedachten gehad en enige intentie gehad om hier naar te handelen?***<br>Zo ja, omschrijven:	**Ja** ☐ **Nee** ☐	**Ja** ☐ **Nee** ☐
**5. Actieve suïcidale ideatie met specifiek plan en intentie**<br>Gedachten over zelfmoord met geheel of gedeeltelijk tot in detail uitgewerkt plan en de deelnemer heeft enige intentie dit uit te voeren.<br>***Hebt u de details van hoe u zichzelf gaat doden al geheel of gedeeltelijk uitgewerkt? Bent u van plan dit ook uit te voeren?***<br>Zo ja, omschrijven:	**Ja** ☐ **Nee** ☐	**Ja** ☐ **Nee** ☐
INTENSITEIT VAN IDEATIE		
De volgende aspecten moeten worden beoordeeld voor de meest ernstige soort ideatie (dat wil zeggen, 1-5 van bovenaf, waarbij 1 het minst ernstig is en 5 het meest ernstig). Vraag naar de tijd dat hij/zich het meest suïcidaal voelde.<br>Leven - ***Meest ernstige ideatie:*** ______ (*Type # (1-5)*) ______ (*Omschrijving van ideatie*)<br>Afgelopen X maanden - ***Meest ernstige ideatie:*** ______ (*Type # (1-5)*) ______ (*Omschrijving van ideatie*)	Meest ernstige	Meest ernstige
**Frequentie**<br>***Hoe vaak hebt u deze gedachten gehad?***<br>(1) Minder dan 1 keer per week (2) 1 keer per week (3) 2-5 keer per week (4) Dagelijks of vrijwel dagelijks (5) Vele keren per dag	___	___
**Duur**<br>***Wanneer u deze gedachten hebt, hoe lang houden ze dan aan?***<br>(1) Kortstondig - paar seconden of minuten<br>(2) Minder dan 1 uur/korte periode<br>(3) 1-4 uur/lange periode<br>(4) 4-8 uur/het merendeel van de dag<br>(5) Meer dan 8 uur/aanhoudend of constant	___	___
**Beheersbaarheid**<br>***Kon/kunt u, als u dat wilt, ophouden met denken aan zelfmoord of de wens om dood te zijn?***<br>(1) Gemakkelijk in staat gedachten onder controle te houden<br>(2) Kan met weinig moeite gedachten onder controle houden<br>(3) Kan met enige moeite gedachten onder controle houden<br>(4) Kan met veel moeite gedachten onder controle houden<br>(5) Niet in staat gedachten onder controle te houden<br>(0) Doet geen poging gedachten onder controle te houden	___	___
**Redenen om geen zelfmoord te plegen**<br>***Zijn er dingen - iemand of iets (bijv. familie, geloof, pijn bij overlijden) - die u ervan hebben weerhouden te willen sterven of uw gedachten over zelfmoord uit te voeren?***<br>(1) Dit soort redenen hebben u zeker weerhouden van het doen van een poging tot zelfmoord<br>(2) Dit soort redenen hebben u waarschijnlijk weerhouden<br>(3) Niet zeker of dit soort redenen u hebben weerhouden<br>(4) Dit soort redenen hebben u zeer waarschijnlijk niet weerhouden<br>(5) Dit soort redenen hebben u zeker niet weerhouden<br>(0) Niet van toepassing	___	___
**Redenen voor ideatie**<br>***Wat voor soort redenen had u om na te denken over uw wens te sterven of uzelf te doden? Was het om een einde te maken aan de pijn of aan hoe u zich voelde (met andere woorden, u kon niet meer met deze pijn leven of met hoe u zich voelde) of was het om aandacht te trekken, wraak te nemen of een reactie bij anderen uit te lokken? Of beide?***<br>(1) Uitsluitend om aandacht te trekken, wraak te nemen, of een reactie bij anderen uit te lokken.<br>(2) Voornamelijk om aandacht te trekken, wraak te nemen, of een reactie bij anderen uit te lokken<br>(3) In gelijke mate om aandacht te trekken, wraak te nemen, of een reactie bij anderen uit te lokken, als om een einde te maken aan de pijn.<br>(4) Voornamelijk om een eind te maken aan de pijn (u kon niet verder leven met de pijn of met hoe u zich voelde).<br>(5) Uitsluitend om een eind te maken aan de pijn (u kon niet verder leven met de pijn of met hoe u zich voelde).<br>(0) Niet van toepassing	___	___

© 2008 Research Foundation for Mental Hygiene, Inc. C-SSRS—Baseline/Screening (Versie 14 januari 2009) Pagina 1 van 2

SUBJECT NUMBER:

VISIT: Screening

SUÏCIDAAL GEDRAG<br>*(Kruis alles aan dat van toepassing is, voor zover het afzonderlijke voorvallen betreft; vraag naar alle typen suïcidale gedragingen)*	**Leven**	**Afgelopen __ jaar**
**Daadwerkelijke poging:**<br>Een handeling waarbij men zichzelf opzettelijk mogelijk letsel toebrengt gepleegd met tenminste enige wens te sterven *als gevolg van die handeling*. Gedrag werd deels beschouwd als methode om zelfmoord te plegen. Intentie hoeft niet 100% te zijn. Als de intentie of de wens om in verband met de handeling te overlijden ook maar ***enigszins*** aanwezig is, kan deze als een daadwerkelijke zelfmoordpoging worden beschouwd. ***Er hoeft geen sprake te zijn van letsel of verwonding***, alleen de mogelijkheid tot letsel of verwonding. Als iemand de trekker overhaalt met het pistool in de mond, maar het pistool is defect waardoor er geen letsel ontstaat, wordt dit als een poging beschouwd. Afgeleide intentie: Zelfs als iemand ontkent dat er een intentie/wens om te sterven is, kan dit klinisch worden afgeleid van het gedrag of de omstandigheden. Bijvoorbeeld, een zeer dodelijke handeling die duidelijk geen ongeluk was, zodat geen andere intentie dan het plegen van zelfmoord kan worden afgeleid (bijv. door het hoofd schieten, springen uit een raam van een hoge verdieping). Ook als iemand ontkent de intentie te hebben te sterven, maar wel meende dat wat hij/zij deed dodelijk zou kunnen zijn, kan er intentie worden afgeleid.<br>***Hebt u een poging tot zelfmoord gedaan?***<br>***Hebt u iets gedaan om uzelf te verwonden?***<br>***Hebt u iets gevaarlijks gedaan waarbij u had kunnen sterven?***<br>***Wat hebt u gedaan?***<br>***Hebt u ______ als een manier om een einde aan uw leven te maken?***<br>***Wilde u dood (al was het maar een beetje) toen u ______?***<br>***Probeerde u een einde aan uw leven te maken toen u ______?***<br>***Of dacht u dat het mogelijk was dat u zou kunnen sterven door______?***<br>***Of deed u het puur om andere redenen/zonder ENIGE intentie om uzelf te doden (zoals het wegnemen van stress, u beter te voelen, medeleven te krijgen, of verandering te brengen in de omstandigheden)?*** (Gedrag waarbij iemand zichzelf letsel toebrengt zonder suïcidale intentie).<br>Zo ja, omschrijven:	Ja ☐ Nee ☐<br><br>Totaal # pogingen<br>______	Ja ☐ Nee ☐<br><br>Totaal # pogingen<br>______
**Heeft de deelnemer niet-suïcidaal gedrag vertoond waarbij hij of zij zichzelf opzettelijk verwondde?**	Ja ☐ Nee ☐	Ja ☐ Nee ☐
**Verhinderde poging:**<br>Wanneer iemand wordt gestoord (door een externe omstandigheid) bij het beginnen van de mogelijk zelfbeschadigende handeling *(als dit niet het geval was geweest, dan zou een daadwerkelijke poging hebben plaatsgevonden)*.<br>Overdosis: Iemand heeft pillen in de hand maar wordt tegengehouden om ze in te nemen. Als er eenmaal pillen zijn ingenomen, dan geldt dit als een poging en niet als een verhinderde poging. Schieten: Iemand heeft het pistool op zichzelf gericht, maar het pistool wordt door iemand anders weggenomen, of de betrokkene wordt op een andere manier verhinderd om de trekker over te halen. Is de trekker eenmaal overgehaald, zelfs als het pistool niet afgaat, dan is dit een poging. Springen: Iemand staat klaar om te springen, maar wordt vastgegrepen en van de richel gehaald. Verhanging: Iemand heeft de strop om zijn nek maar is nog niet begonnen met de verhanging - wordt hiervan weerhouden. ***Is er een moment geweest waarop u begon iets te doen om uw leven te beëindigen, maar iets of iemand u stopte voordat u daadwerkelijk iets had gedaan?***<br>Zo ja, omschrijven:	Ja ☐ Nee ☐<br><br>Totaal # verhinderde pogingen<br>______	Ja ☐ Nee ☐<br><br>Totaal # verhinderde pogingen<br>______
**Afgebroken poging:**<br>Wanneer iemand stappen begint te ondernemen om een suïcidepoging te doen, maar zichzelf tegenhoudt voordat hij/zij daadwerkelijk enig zelfdestructief gedrag heeft vertoond. De voorbeelden zijn vergelijkbaar met de verhinderde pogingen, alleen houdt de betrokkene nu zichzelf tegen in plaats van dat hij/zij wordt tegengehouden door iemand anders.<br>***Is er een moment geweest waarop u begon iets te doen om te proberen uw leven te beëindigen, maar dat u zichzelf ervan weerhield voordat u daadwerkelijk iets had gedaan?***<br>Zo ja, omschrijven:	Ja ☐ Nee ☐<br><br>Totaal # afgebroken pogingen<br>______	Ja ☐ Nee ☐<br><br>Totaal # afgebroken pogingen<br>______
**Voorbereidende handelingen of gedrag:**<br>Handelingen of voorbereiding voor een naderende suïcidepoging. Dit kan van alles zijn dat verder gaat dan een uitspraak of een gedachte, bijv. dingen verzamelen ten behoeve van een bepaalde methode (zoals het kopen van pillen, het aanschaffen van een pistool) of het voorbereiden op de dood door zelfmoord (bijv. spullen weggeven, een afscheidsbrief schrijven).<br>***Hebt u stappen ondernomen om een zelfmoordpoging te doen of u voor te bereiden op het plegen van zelfmoord (door bijvoorbeeld het verzamelen van pillen, het aanschaffen van een pistool, het weggeven van waardevolle spullen of het schrijven van een afscheidsbrief)?***<br>Zo ja, omschrijven:	Ja ☐ Nee ☐	Ja ☐ Nee ☐
**Suïcidaal gedrag:**<br>Suïcidaal gedrag was aanwezig tijdens de beoordelingsperiode?	Ja ☐ Nee ☐	Ja ☐ Nee ☐

Alleen beantwoorden bij daadwerkelijke pogingen	Meest recente poging Datum:	Dodelijke poging Datum:	Eerste poging Datum:
**Daadwerkelijke letaliteit/Medisch letsel:**<br>0. Geen fysiek letsel of zeer gering fysiek letsel (bijvoorbeeld schrammen).<br>1. Gering fysiek letsel (bijvoorbeeld trage spraak, eerstegraads brandwonden, lichte bloeding, verstuikingen).<br>2. Gematigd fysiek letsel; medische zorg nodig (bijvoorbeeld bij bewustzijn maar versuft, enigszins reagerend, tweedegraads brandwonden, bloeding van grote ader).<br>3. Gematigd ernstig fysiek letsel; opname in *ziekenhuis* en waarschijnlijk intensieve zorg noodzakelijk (bijv. comateus met intacte reflexen, derdegraads brandwonden over minder dan 20% van het lichaam, ernstig bloedverlies maar kan herstellen, ernstige botbreuken).<br>4. Ernstig fysiek letsel; opname in *ziekenhuis* en intensieve zorg noodzakelijk (bijvoorbeeld comateus zonder reflexen, derdegraads brandwonden over meer dan 20% van het lichaam, aanzienlijk bloedverlies met instabiele vitale tekens, aanzienlijk letsel aan vitaal gebied).<br>5. Overlijden	*Voer Code in*<br>______	*Voer Code in*<br>______	*Voer Code in*<br>______
**Mogelijke letaliteit: Alleen beantwoorden indien daadwerkelijke letaliteit = 0**<br>De waarschijnlijke letaliteit van daadwerkelijke poging indien geen medisch letsel (de volgende voorbeelden leiden weliswaar niet tot medisch letsel, maar de letaliteit had zeer hoog geweest kunnen zijn: iemand doet pistool in mond en haalt trekker over, maar pistool weigert, dus geen medisch letsel; betrokkene ligt op de rails terwijl een trein nadert, maar wordt weggetrokken voordat deze onder de trein komt).<br>0 = Gedrag dat waarschijnlijk niet tot letsel leidt<br>1 = Gedrag leidt waarschijnlijk tot letsel, maar niet waarschijnlijk tot de dood<br>2 = Gedrag leidt waarschijnlijk tot de dood, ondanks de medische zorg die voorhanden is	*Voer Code in*<br>______	*Voer Code in*<br>______	*Voer Code in*<br>______

© 2008 Research Foundation for Mental Hygiene, Inc.

APPENDIX C: COLUMBIA SUICIDE SEVERITY RATING SCALE (C-SSRS) – SINCE LAST VISIT

DATE: d d m m m y y y y   TIME: 24 hour clock

Please Apply Subject Information Label Here

SUÏCIDALE IDEATIE	
Stel vraag 1 en 2. Indien beide vragen negatief worden beantwoord: ga naar het onderdeel 'Suïcidaal Gedrag'. Indien vraag 2 met 'ja' wordt beantwoord, stel dan vraag 3, 4 en 5. Indien het antwoord op vraag 1 en/of vraag 2 'ja' is, vul het onderdeel 'intensiteit van ideatie' in.	**Sinds laatste bezoek**
**1. Wens om dood te zijn**<br>Deelnemer koestert gedachten over de wens om dood te zijn, of niet langer meer te leven, of de wens om in slaap te vallen en niet meer wakker te worden.<br>***Hebt u gewenst dat u dood was of dat u kon gaan slapen en niet meer wakker zou worden?***<br>Zo ja, omschrijven:	Ja ☐ Nee ☐
**2. Niet-specifieke actieve suïcidale gedachten**<br>Algemene niet-specifieke gedachten over het willen beëindigen van je leven/zelfmoord te plegen (bijvoorbeeld: *'Ik heb aan zelfmoord gedacht'*) zonder gedachten aan manieren om zichzelf te doden/geassocieerde methoden, voornemen of plan tijdens de beoordelingsperiode.<br>***Hebt u daadwerkelijk wel eens gedacht aan zelfmoord?***<br>Zo ja, omschrijven:	Ja ☐ Nee ☐
**3. Actieve suïcidale ideatie met ongeacht welke methode (zonder plan) zonder intentie om te handelen**<br>De deelnemer koestert gedachten over suïcide en heeft tijdens de beoordelingsperiode aan ten minste één methode gedacht. Dit is anders dan een specifiek plan waarin de details voor wat betreft de tijd, plaats of methode zijn uitgewerkt (bijvoorbeeld de gedachte aan een methode voor zelfmoord, maar geen specifiek plan). Hieronder valt iemand die zou zeggen: *'Ik heb er over gedacht om een overdosis te nemen, maar nooit een specifiek plan gemaakt waar, wanneer of hoe ik dit eigenlijk zou doen... en ik zou het nooit doorzetten'.*<br>***Hebt u nagedacht over hoe u dit misschien zou doen?***<br>Zo ja, omschrijven:	Ja ☐ Nee ☐
**4. Actieve suïcidale ideatie met enige intentie om te handelen, zonder specifiek plan**<br>Actieve suïcidale gedachten over zelfmoord en deelnemer geeft aan enige intentie te hebben naar zulke gedachten te handelen, in tegenstelling tot *'Ik heb die gedachten maar zou er zeker nooit iets mee doen'.*<br>***Hebt u deze gedachten gehad en enige intentie gehad om hier naar te handelen?***<br>Zo ja, omschrijven:	Ja ☐ Nee ☐
**5. Actieve suïcidale ideatie met specifiek plan en intentie**<br>Gedachten over zelfmoord met geheel of gedeeltelijk tot in detail uitgewerkt plan en de deelnemer heeft enige intentie dit uit te voeren.<br>***Hebt u de details van hoe u zichzelf gaat doden al geheel of gedeeltelijk uitgewerkt? Bent u van plan dit ook uit te voeren?***<br>Zo ja, omschrijven:	Ja ☐ Nee ☐
INTENSITEIT VAN IDEATIE	
De volgende aspecten moeten worden beoordeeld voor de meest ernstige soort ideatie (dat wil zeggen, 1-5 van bovenaf, waarbij 1 het minst ernstig is en 5 het meest ernstig).<br>***Meest ernstige ideatie:*** ______ ***Type # (1-5)*** ______ ***Omschrijving van ideatie***	Meest ernstige
**Frequentie**<br>***Hoe vaak hebt u deze gedachten gehad?***<br>(1) Minder dan 1 keer per week (2) 1 keer per week (3) 2-5 keer per week (4) Dagelijks of vrijwel dagelijks (5) Vele keren per dag	___
**Duur**<br>***Wanneer u deze gedachten hebt, hoe lang houden ze dan aan?***<br>(1) Kortstondig - paar seconden of minuten<br>(2) Minder dan 1 uur/korte periode<br>(3) 1-4 uur/lange periode<br>(4) 4-8 uur/het merendeel van de dag<br>(5) Meer dan 8 uur/aanhoudend of constant	___
**Beheersbaarheid**<br>***Kon/kunt u, als u dat wilt, ophouden met denken aan zelfmoord of de wens om dood te zijn?***<br>(1) Gemakkelijk in staat gedachten onder controle te houden<br>(2) Kan met weinig moeite gedachten onder controle houden<br>(3) Kan met enige moeite gedachten onder controle houden<br>(4) Kan met veel moeite gedachten onder controle houden<br>(5) Niet in staat gedachten onder controle te houden<br>(0) Doet geen poging gedachten onder controle te houden	___
**Redenen om geen zelfmoord te plegen**<br>***Zijn er dingen - iemand of iets (bijv. familie, geloof, pijn bij overlijden) - die u ervan hebben weerhouden te willen sterven of uw gedachten over zelfmoord uit te voeren?***<br>(1) Dit soort redenen hebben u zeker weerhouden van het doen van een poging tot zelfmoord<br>(2) Dit soort redenen hebben u waarschijnlijk weerhouden<br>(3) Niet zeker of dit soort redenen u hebben weerhouden<br>(4) Dit soort redenen hebben u zeer waarschijnlijk niet weerhouden<br>(5) Dit soort redenen hebben u zeker niet weerhouden<br>(0) Niet van toepassing	___
**Redenen voor ideatie**<br>***Wat voor soort redenen had u om na te denken over uw wens te sterven of uzelf te doden? Was het om een einde te maken aan de pijn of aan hoe u zich voelde (met andere woorden, u kon niet meer met deze pijn leven of met hoe u zich voelde) of was het om aandacht te trekken, wraak te nemen of een reactie bij anderen uit te lokken? Of beide?***<br>(1) Uitsluitend om aandacht te trekken, wraak te nemen, of een reactie bij anderen uit te lokken<br>(2) Voornamelijk om aandacht te trekken, wraak te nemen, of een reactie bij anderen uit te lokken<br>(3) In gelijke mate om aandacht te trekken, wraak te nemen, of een reactie bij anderen uit te lokken, als om een einde te maken aan de pijn<br>(4) Voornamelijk om een eind te maken aan de pijn (u kon niet verder leven met de pijn of met hoe u zich voelde)<br>(5) Uitsluitend om een eind te maken aan de pijn (u kon niet verder leven met de pijn of met hoe u zich voelde)<br>(0) Niet van toepassing	___

© 2008 Research Foundation for Mental Hygiene, Inc. C-SSRS—Sinds laatste bezoek (Versie 14 januari 2009)

Please Apply
Subject Information
Label Here

SUÏCIDAAL GEDRAG

(Kruis alles aan dat van toepassing is, voor zover het afzonderlijke voorvallen betreft; vraag naar alle typen suïcidale gedragingen)

Sinds laatste bezoek

Daadwerkelijke poging: (Ja ☐ Nee ☐)

Een handeling waarbij men zichzelf opzettelijk mogelijk letsel toebrengt gepleegd met ten minste enige wens te sterven *als gevolg van die handeling*. Gedrag werd deels beschouwd als methode om zelfmoord te plegen. Intentie hoeft niet 100% te zijn. Als de intentie of de wens om in verband met de handeling te overlijden ook maar ***enigszins*** aanwezig is, kan deze als een daadwerkelijke zelfmoordpoging worden beschouwd. ***Er hoeft geen sprake te zijn van letsel of verwonding***, alleen de mogelijkheid tot letsel of verwonding. Als iemand de trekker overhaalt met het pistool in de mond, maar het pistool is defect waardoor er geen letsel ontstaat, wordt dit als een poging beschouwd.

Afgeleide intentie: Zelfs als iemand ontkent dat er een intentie/wens om te sterven is, kan dit klinisch worden afgeleid van het gedrag of de omstandigheden. Bijvoorbeeld, een zeer dodelijke handeling die duidelijk geen ongeluk was, zodat geen andere intentie dan het plegen van zelfmoord kan worden afgeleid (bijv. door het hoofd schieten, springen uit een raam van een hoge verdieping). Ook als iemand ontkent de intentie te hebben te sterven, maar wel meende dat wat hij/zij deed dodelijk zou kunnen zijn, kan er intentie worden afgeleid.

Hebt u een poging tot zelfmoord gedaan?
Hebt u iets gedaan om uzelf te verwonden?
Hebt u iets gevaarlijks gedaan waarbij u had kunnen sterven?
- ***Wat hebt u gedaan?***
- ***Hebt u ______ als een manier om een einde aan uw leven te maken?***
- ***Wilde u dood (al was het maar een beetje) toen u ______?***
- ***Probeerde u een einde aan uw leven te maken toen u ______?***
- ***Of dacht u dat het mogelijk was dat u zou kunnen sterven door______?***

Of deed u het puur om andere redenen/zonder ENIGE intentie om uzelf te doden (zoals het wegnemen van stress, u beter te voelen, medeleven te krijgen, of verandering te brengen in de omstandigheden)? (Gedrag waarbij iemand zichzelf letsel toebrengt zonder suïcidale intentie)

Zo ja, omschrijven:

Totaal # pogingen ______

Heeft de deelnemer niet-suïcidaal gedrag vertoond waarbij hij of zij zichzelf opzettelijk verwondde? (Ja ☐ Nee ☐)

Verhinderde poging: (Ja ☐ Nee ☐)

Wanneer iemand wordt gestoord (door een externe omstandigheid) bij het beginnen van de mogelijk zelfbeschadigende handeling *(als dit niet het geval was geweest, dan zou een daadwerkelijke poging hebben plaatsgevonden)*. Overdosis: Iemand heeft pillen in de hand maar wordt tegengehouden om ze in te nemen. Als er eenmaal pillen zijn ingenomen, dan geldt dit als een poging en niet als een verhinderde poging. Schieten: Iemand heeft het pistool op zichzelf gericht, maar het pistool wordt door iemand anders weggenomen, of de betrokkene wordt op een andere manier verhinderd om de trekker over te halen. Is de trekker eenmaal overgehaald, zelfs als het pistool niet afgaat, dan is dit een poging. Springen: Iemand staat klaar om te springen, maar wordt vastgegrepen en van de richel gehaald. Verhanging: Iemand heeft de strop om zijn nek maar is nog niet begonnen met de verhanging - wordt hiervan weerhouden.

Is er een moment geweest waarop u begon iets te doen om uw leven te beëindigen, maar iets of iemand u stopte voordat u daadwerkelijk iets had gedaan?

Zo ja, omschrijven:

Totaal # verhinderde pogingen ______

Afgebroken poging: (Ja ☐ Nee ☐)

Wanneer iemand stappen begint te ondernemen om een suïcidepoging te doen, maar zichzelf tegenhoudt voordat hij/zij daadwerkelijk enig zelfdestructief gedrag heeft vertoond. De voorbeelden zijn vergelijkbaar met de verhinderde pogingen, alleen houdt de betrokkene nu zichzelf tegen in plaats van dat hij/zij wordt tegengehouden door iemand anders.

Is er een moment geweest waarop u begon iets te doen om te proberen uw leven te beëindigen, maar dat u zichzelf ervan weerhield voordat u daadwerkelijk iets had gedaan?

Zo ja, omschrijven:

Totaal # afgebroken pogingen ______

Voorbereidende handelingen of gedrag: (Ja ☐ Nee ☐)

Handelingen of voorbereiding voor een naderende suïcidepoging. Dit kan van alles zijn dat verder gaat dan een uitspraak of een gedachte, bijv. dingen verzamelen ten behoeve van een bepaalde methode (zoals het kopen van pillen, het aanschaffen van een pistool) of het voorbereiden op de dood door zelfmoord (bijv. spullen weggeven, een afscheidsbrief schrijven).

Hebt u stappen ondernomen om een zelfmoordpoging te doen of u voor te bereiden op het plegen van zelfmoord (door bijvoorbeeld het verzamelen van pillen, het aanschaffen van een pistool, het weggeven van waardevolle spullen of het schrijven van een afscheidsbrief)?

Zo ja, omschrijven:

Suïcidaal gedrag: (Ja ☐ Nee ☐)

Suïcidaal gedrag was aanwezig tijdens de beoordelingsperiode?

Suïcide: (Ja ☐ Nee ☐)

Alleen beantwoorden bij daadwerkelijke pogingen

Dodelijke poging Datum:

Daadwerkelijke letaliteit/Medisch letsel: (*Voer Code in* ______)

0. Geen fysiek letsel of zeer gering fysiek letsel (bijvoorbeeld schrammen)
1. Gering fysiek letsel (bijvoorbeeld trage spraak, eerstegraads brandwonden, lichte bloeding, verstuikingen)
2. Gematigd fysiek letsel; medische zorg nodig (bijvoorbeeld bij bewustzijn maar versuft, enigszins reagerend, tweedegraads brandwonden, bloeding van grote ader).
3. Gematigd ernstig fysiek letsel; opname in *ziekenhuis* en waarschijnlijk intensieve zorg noodzakelijk (bijv. comateus met intacte reflexen, derdegraads brandwonden over minder dan 20% van het lichaam, ernstig bloedverlies maar kan herstellen, ernstige botbreuken)
4. Ernstig fysiek letsel; opname in *ziekenhuis* en intensieve zorg noodzakelijk (bijvoorbeeld comateus zonder reflexen, derdegraads brandwonden over meer dan 20% van het lichaam, aanzienlijk bloedverlies met instabiele vitale tekens, aanzienlijk letsel aan vitaal gebied)
5. Overlijden

Mogelijke letaliteit: Alleen beantwoorden indien daadwerkelijke letaliteit = 0 (*Voer Code in* ______)

De waarschijnlijke letaliteit van daadwerkelijke poging indien geen medisch letsel (de volgende voorbeelden leiden weliswaar niet tot medisch letsel, maar de letaliteit had zeer hoog geweest kunnen zijn: iemand doet pistool in mond en haalt trekker over, maar pistool weigert, dus geen medisch letsel; betrokkene ligt op de rails terwijl een trein nadert, maar wordt weggetrokken voordat deze onder de trein komt).

0 = Gedrag dat waarschijnlijk niet tot letsel leidt
1 = Gedrag leidt waarschijnlijk tot letsel, maar niet waarschijnlijk tot de dood
2 = Gedrag leidt waarschijnlijk tot de dood, ondanks de medische zorg die voorhanden is

© 2008 Research Foundation for Mental Hygiene, Inc. C-SSRS—Sinds laatste bezoek (Versie 14 januari 2009)

APPENDIX D: SEDATION VISUAL ANALOG SCALE

How sedated are you?

Not at all ______________________________ Almost asleep
